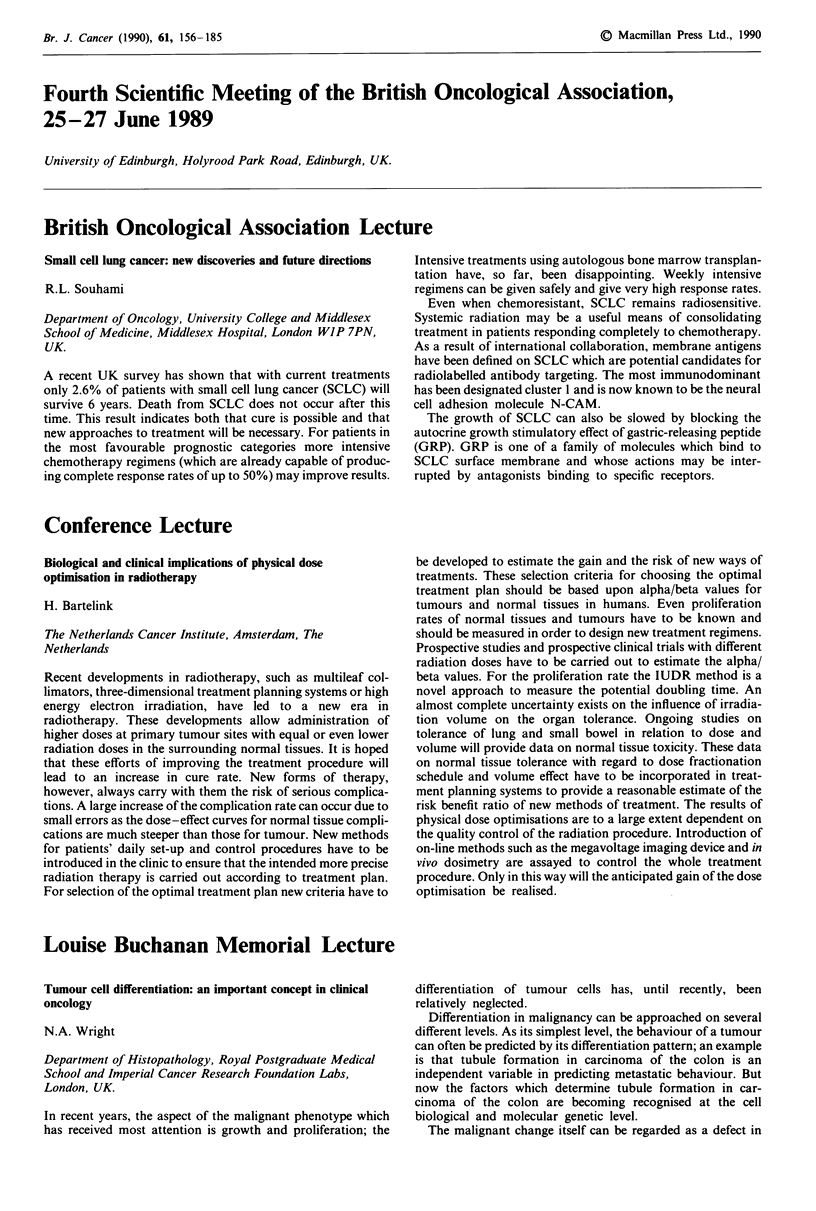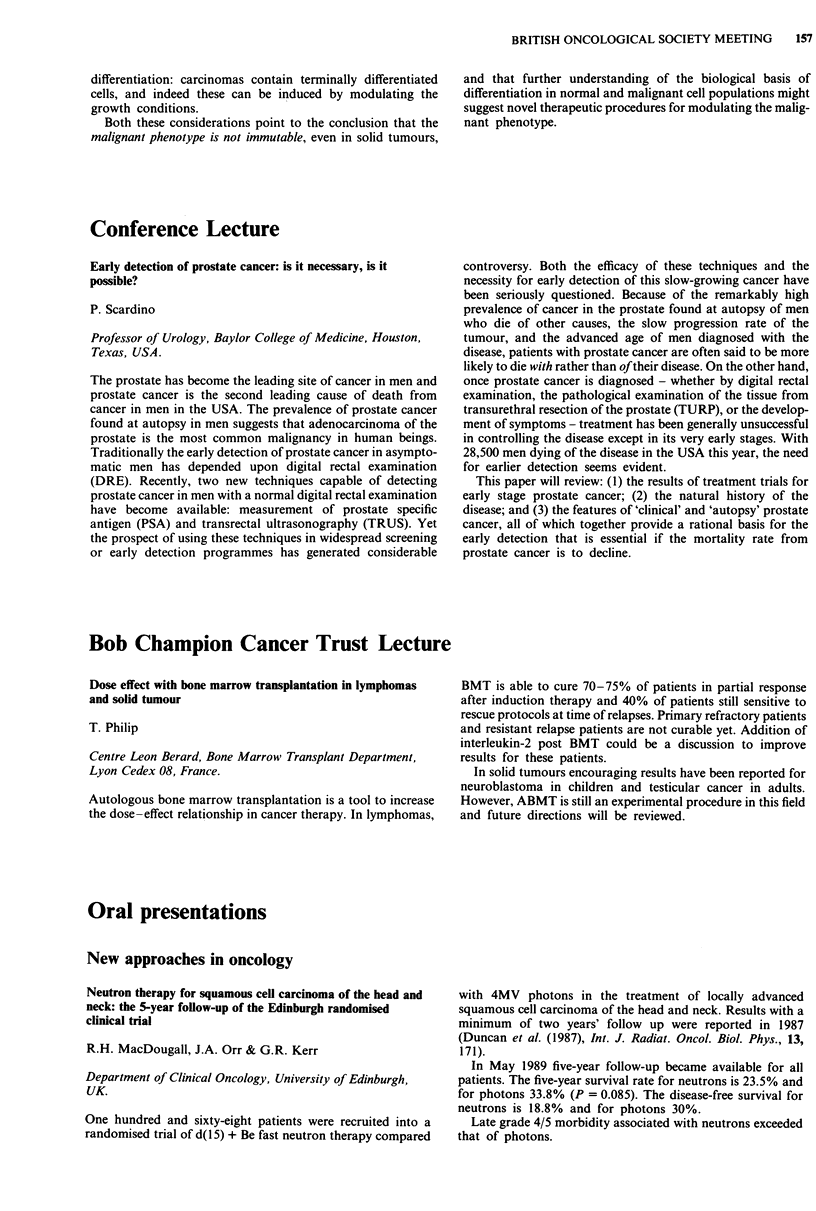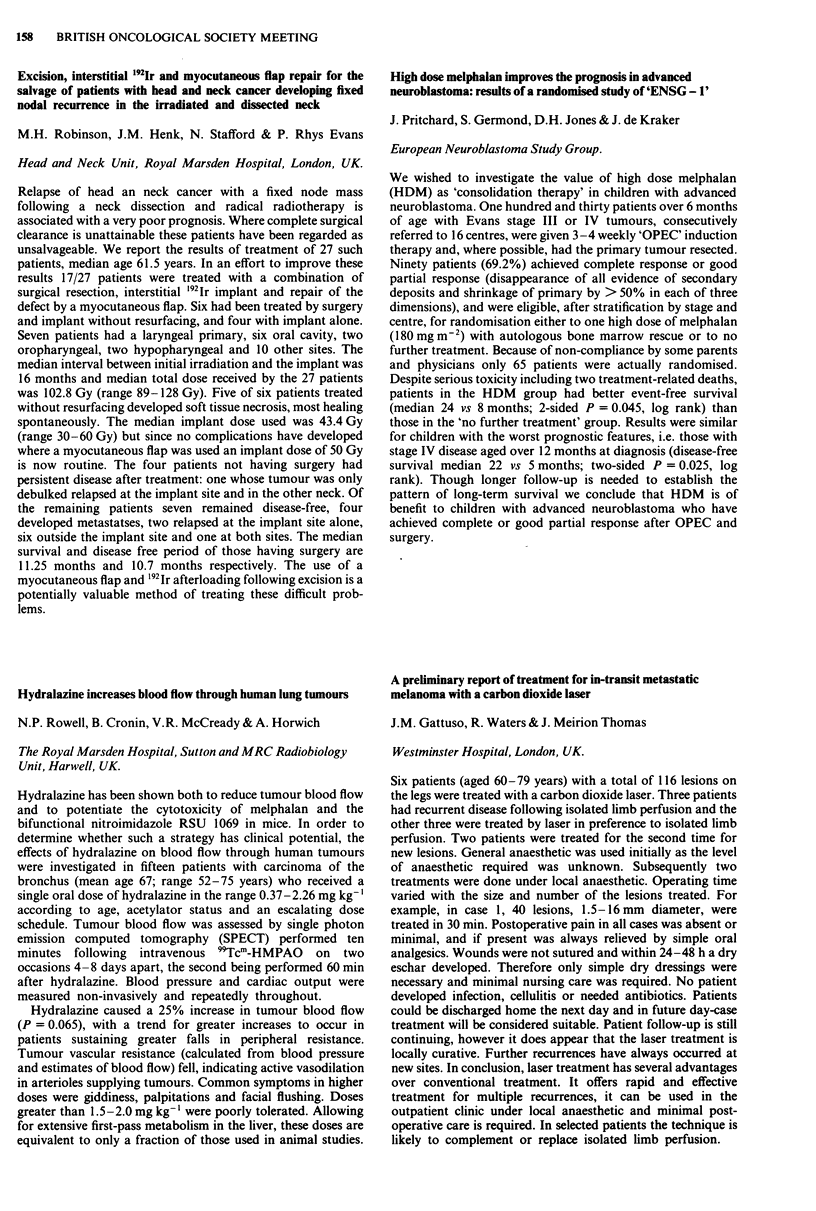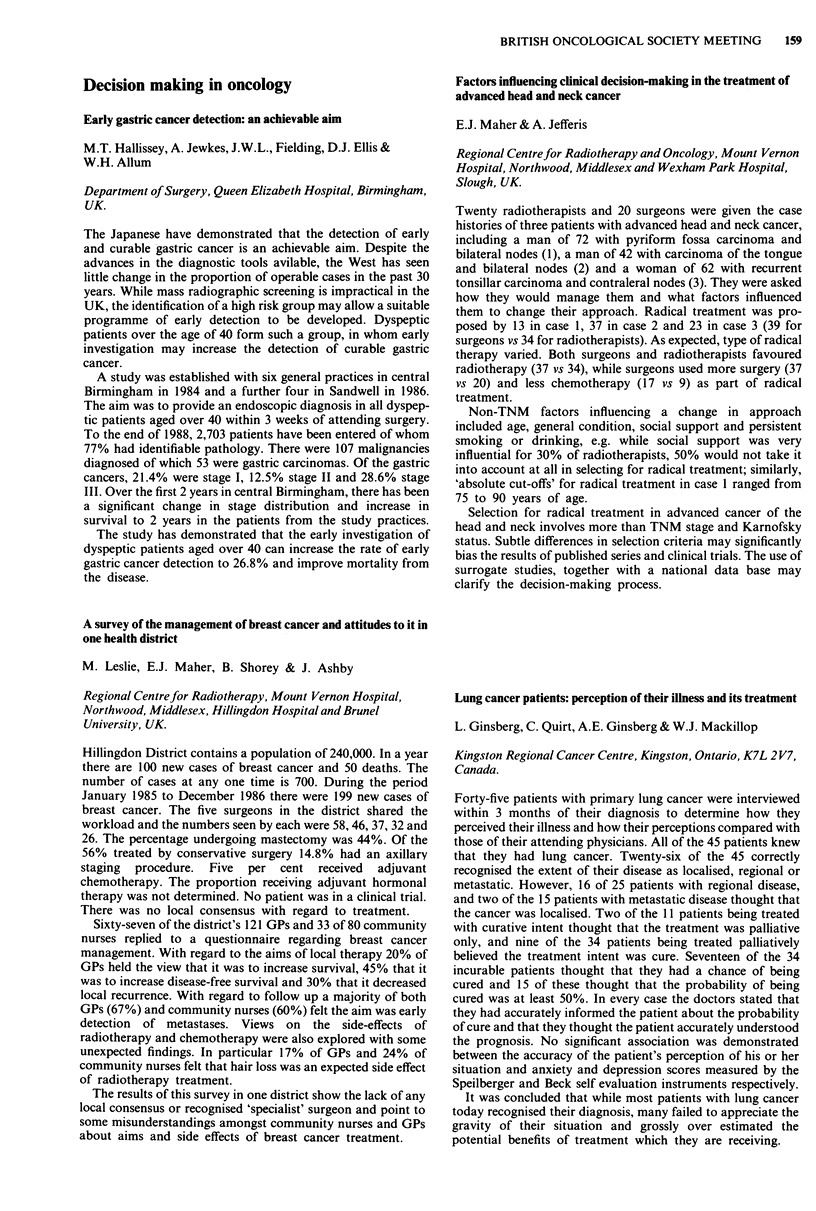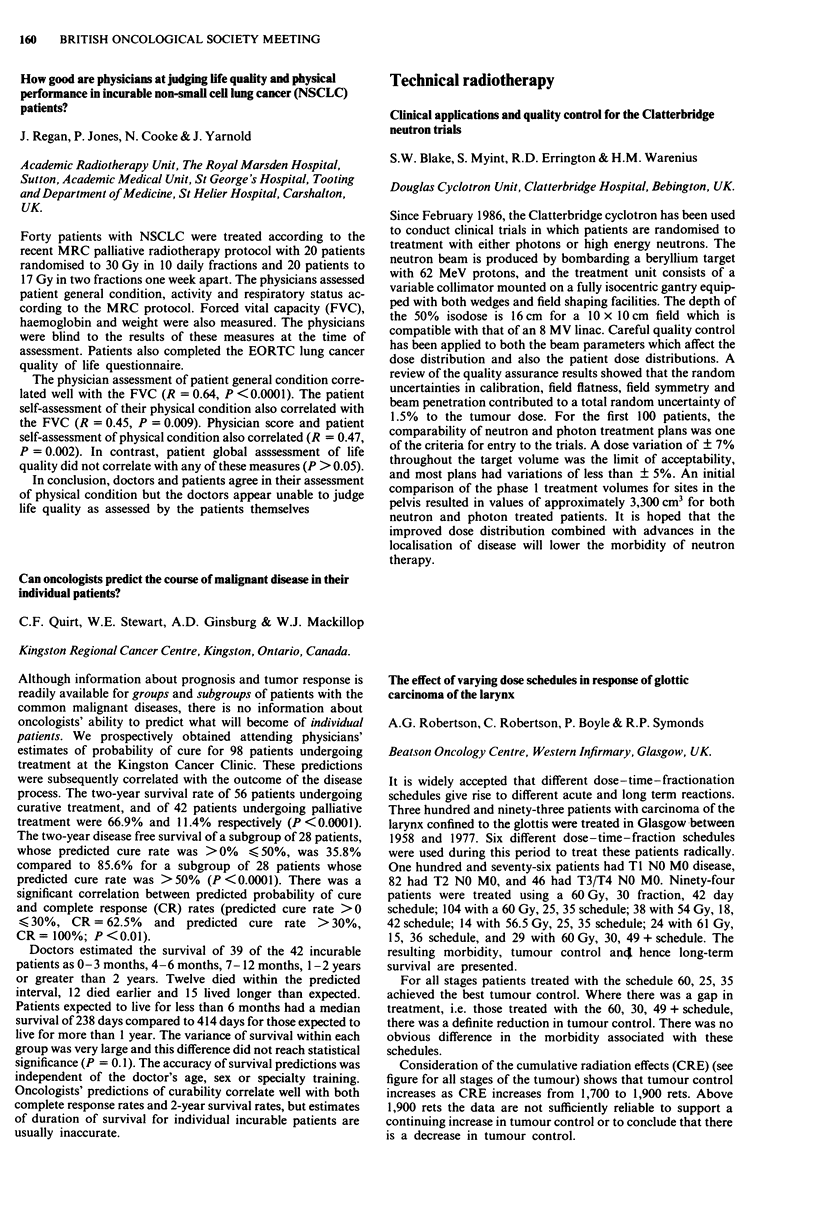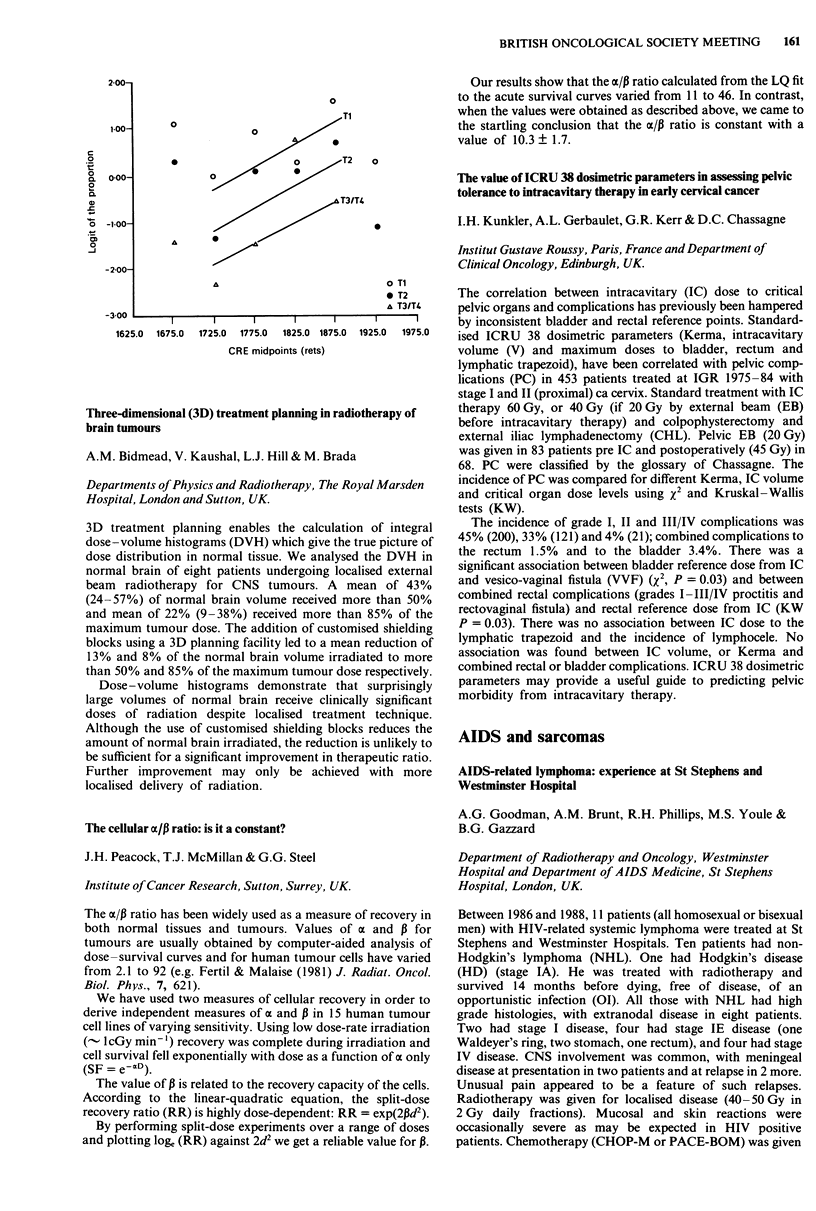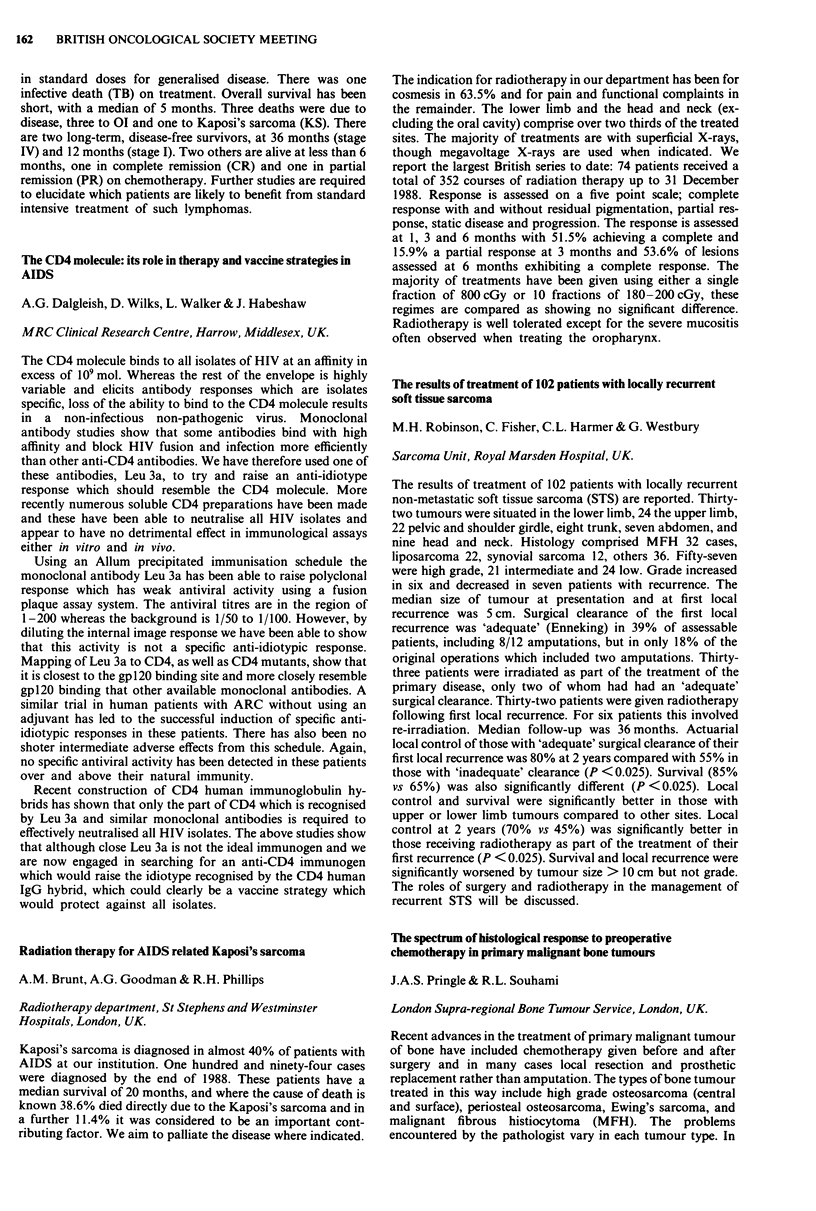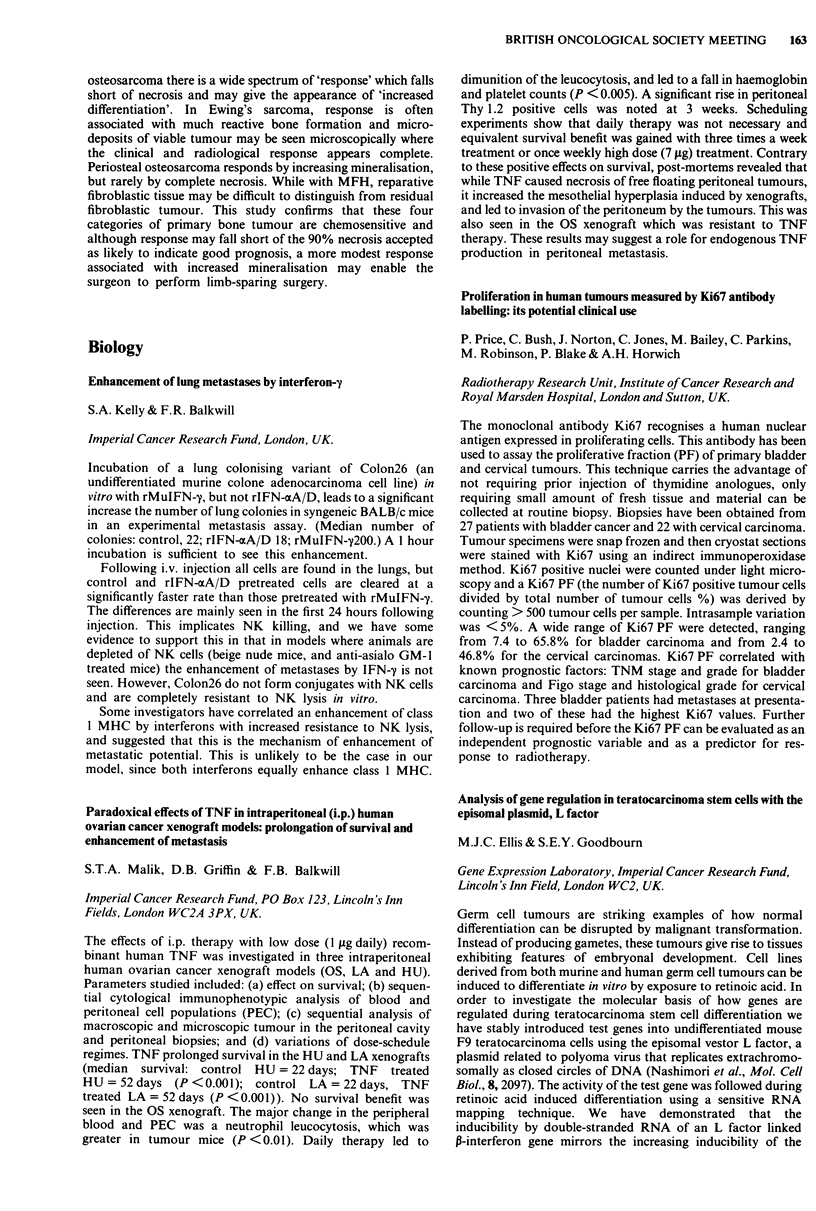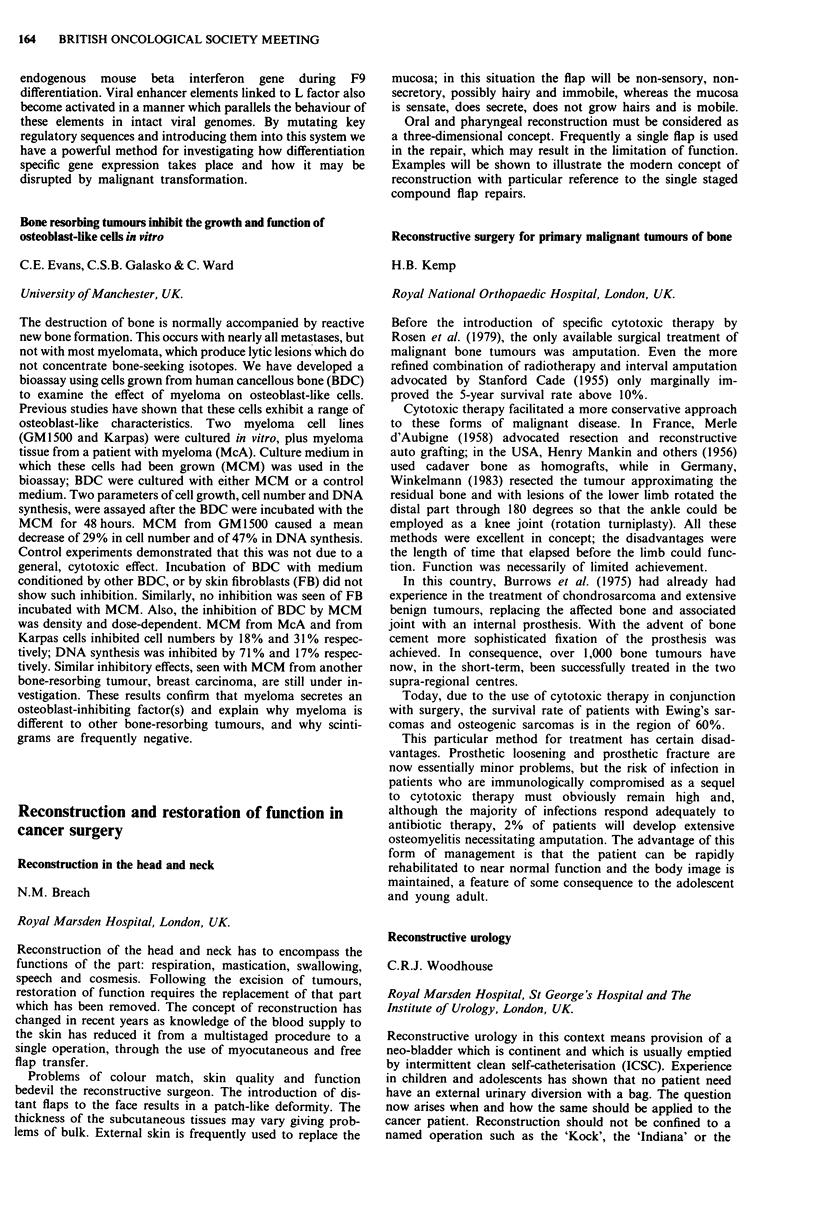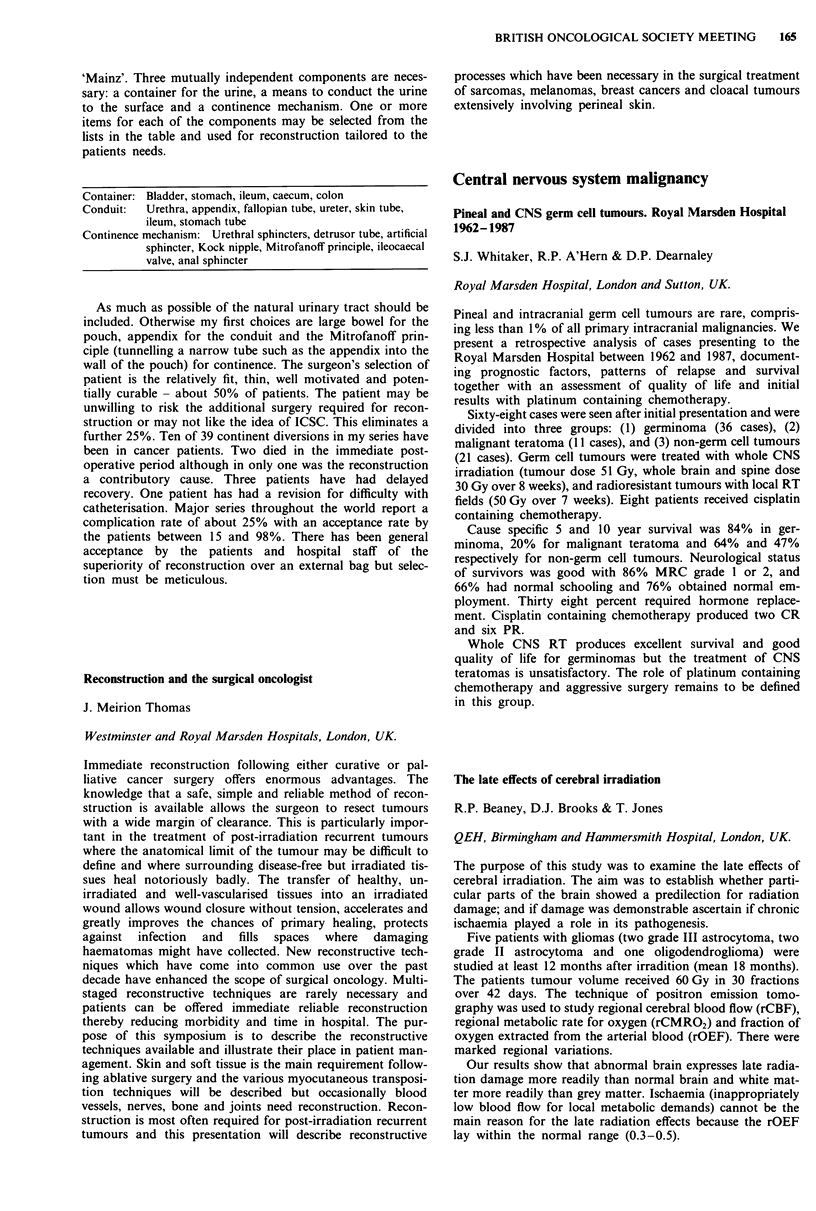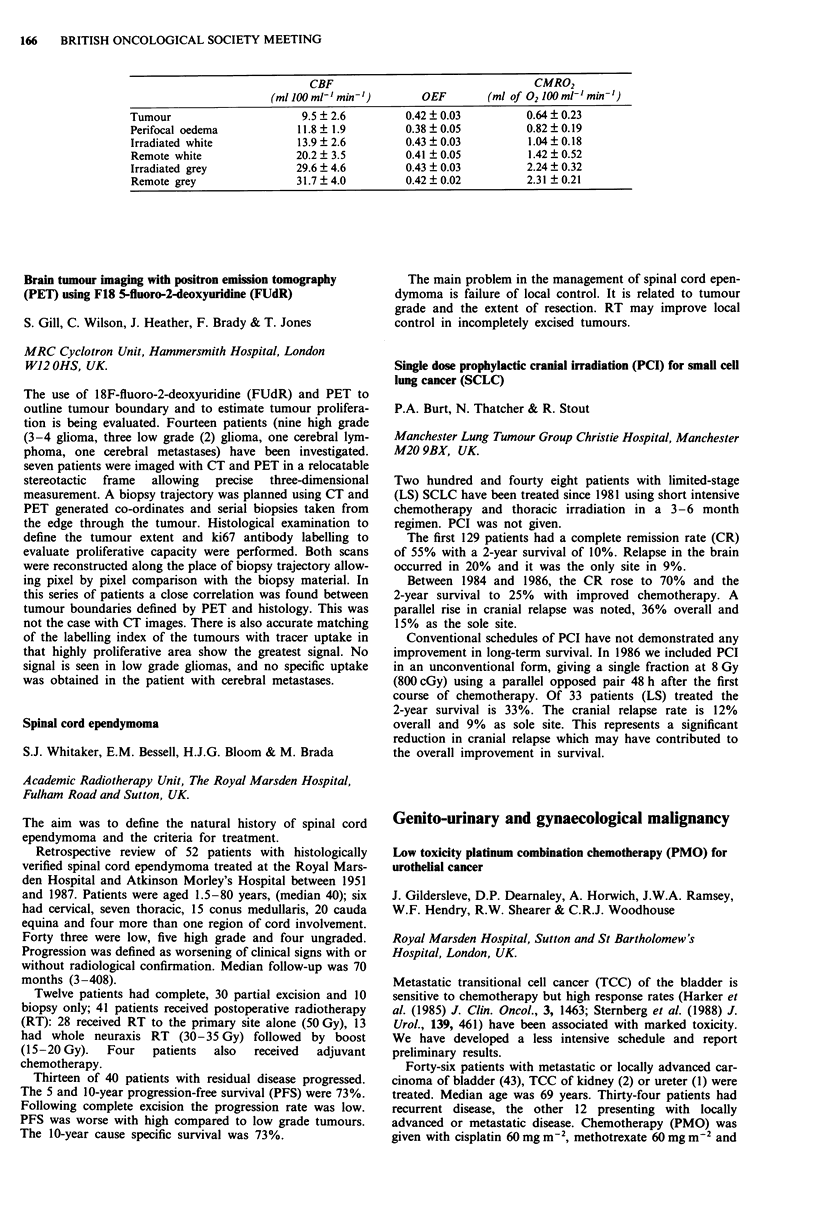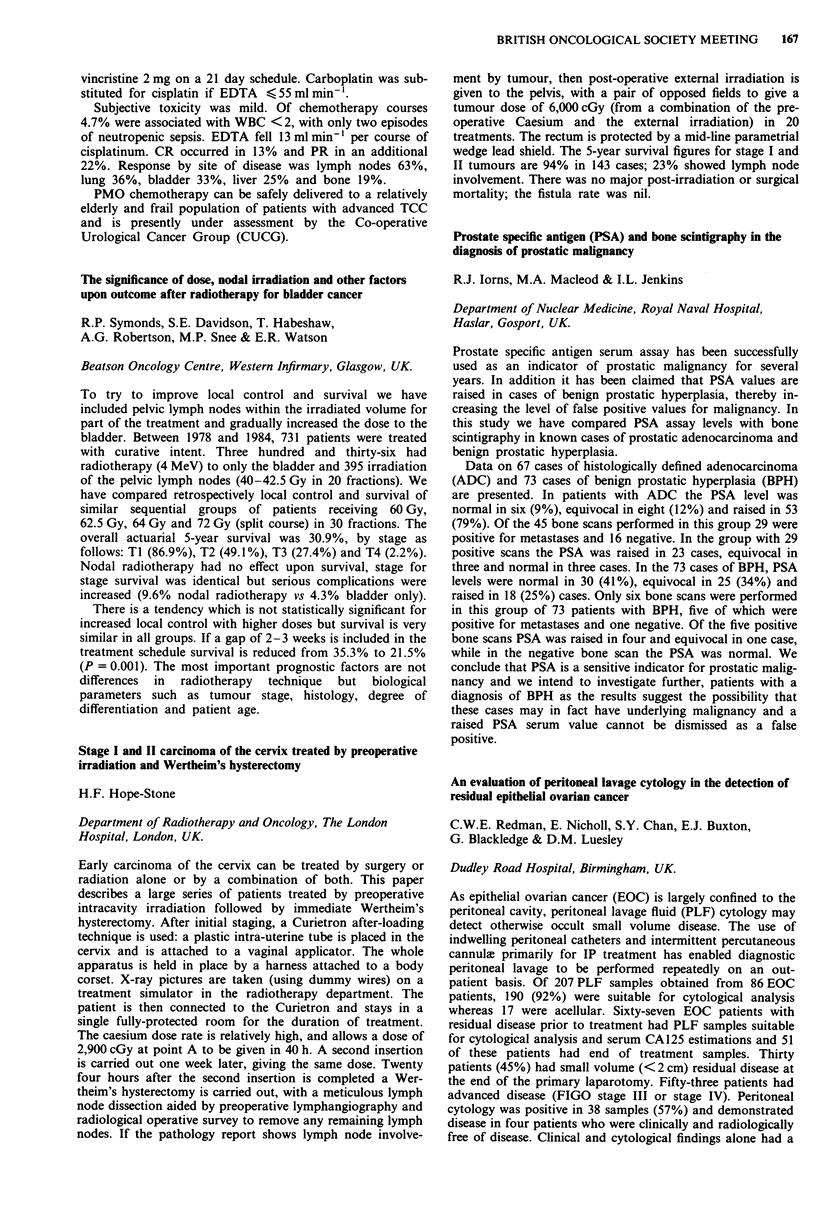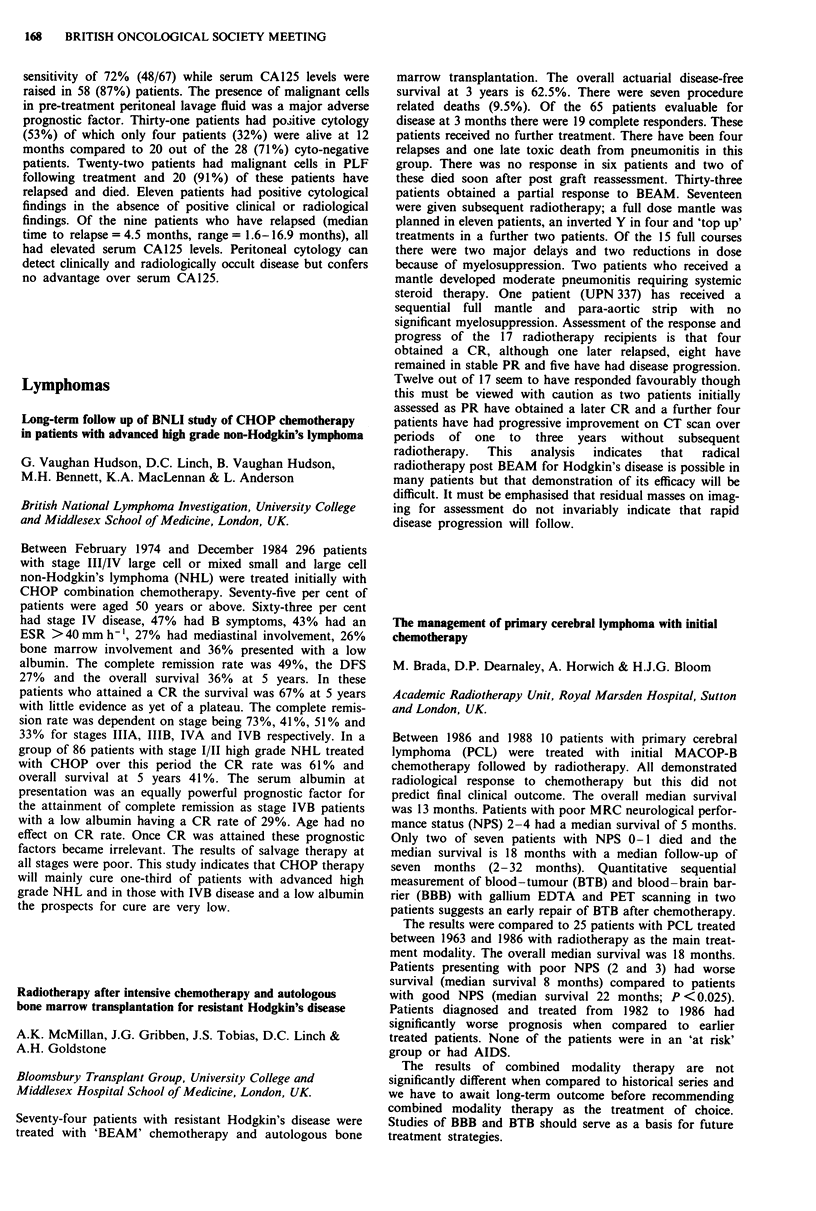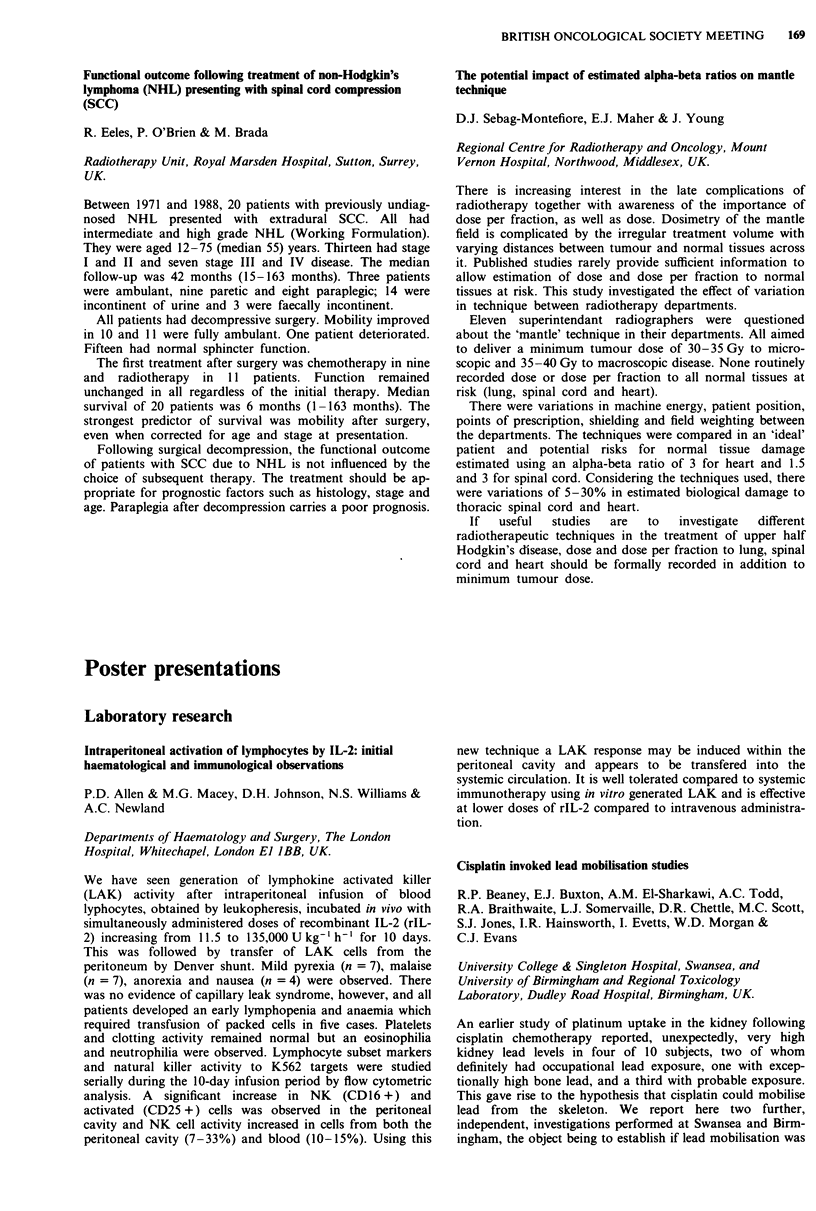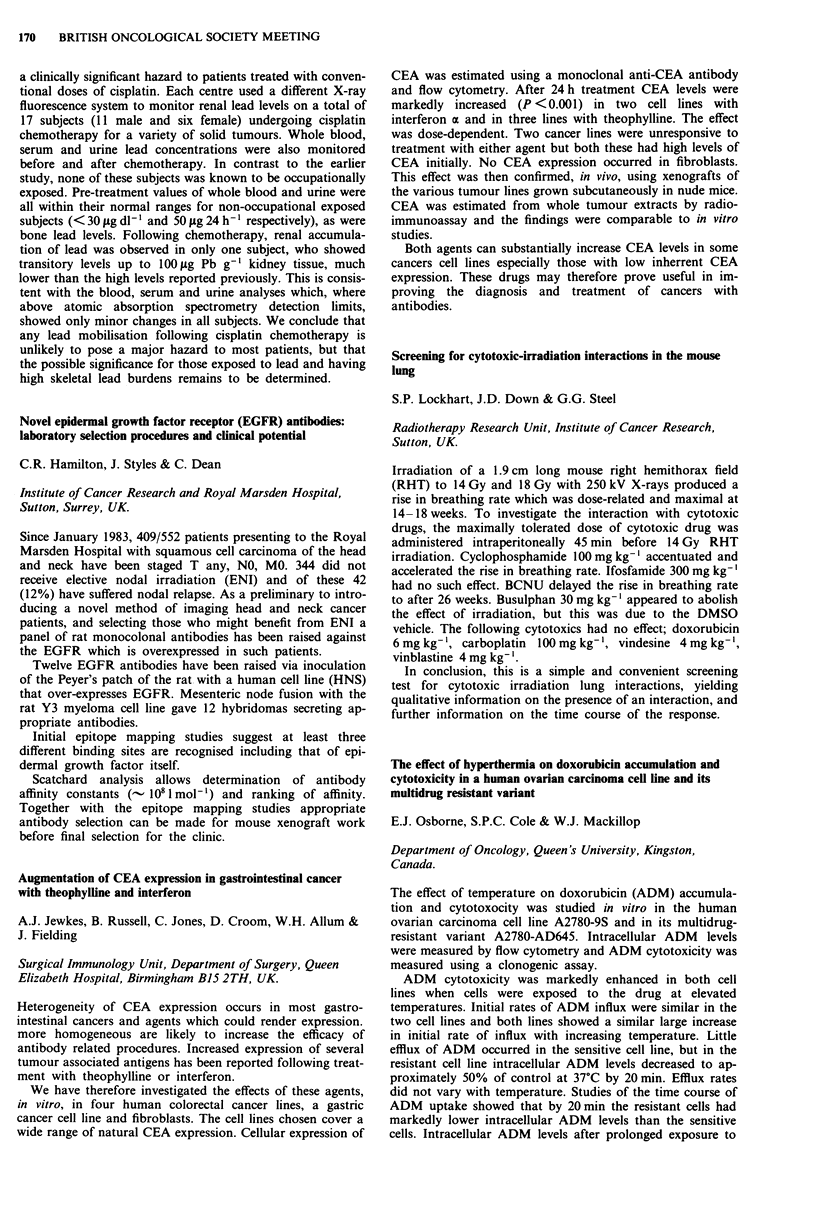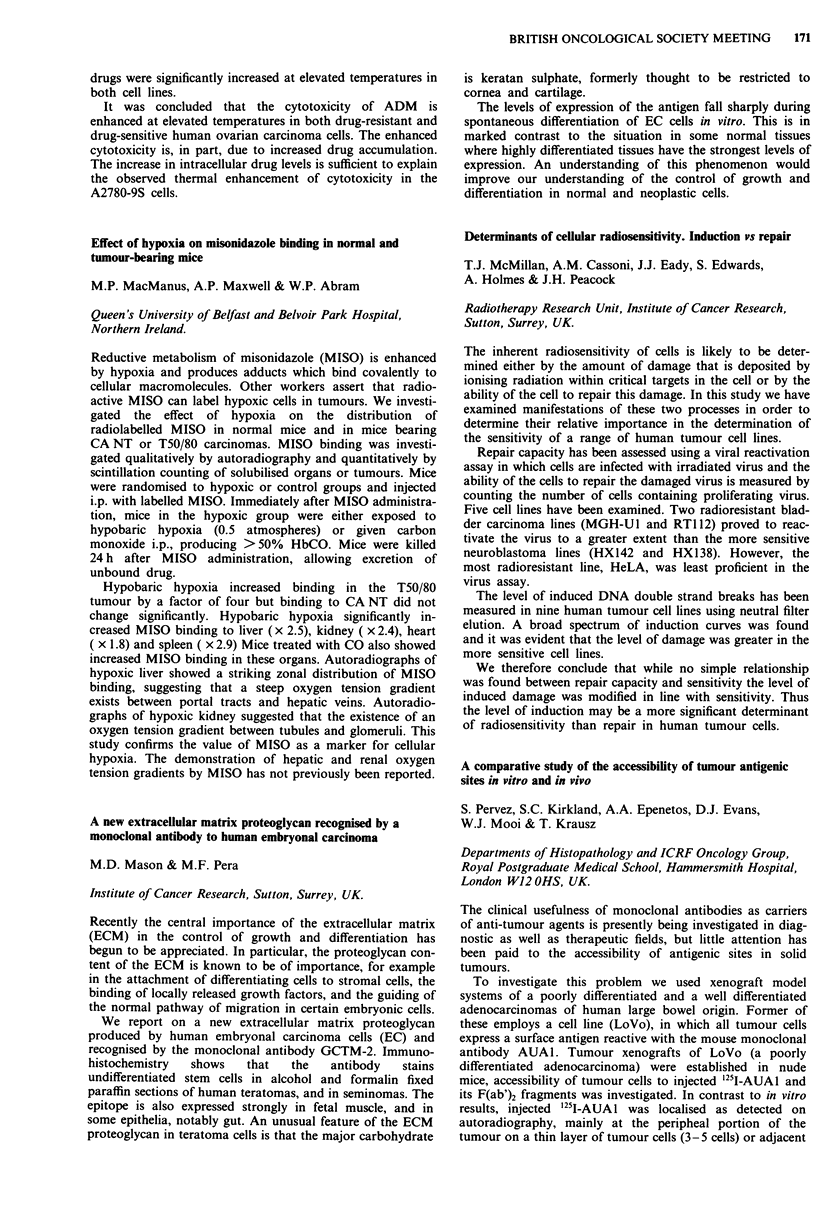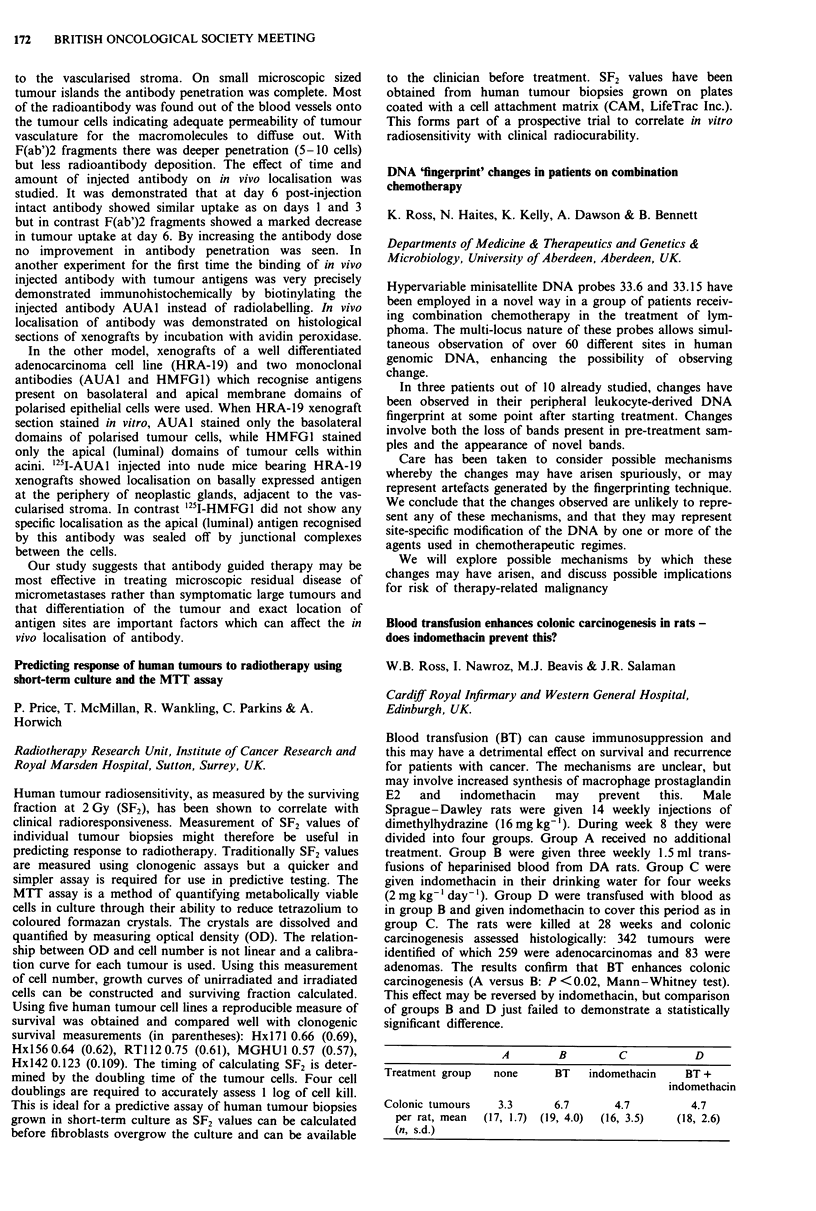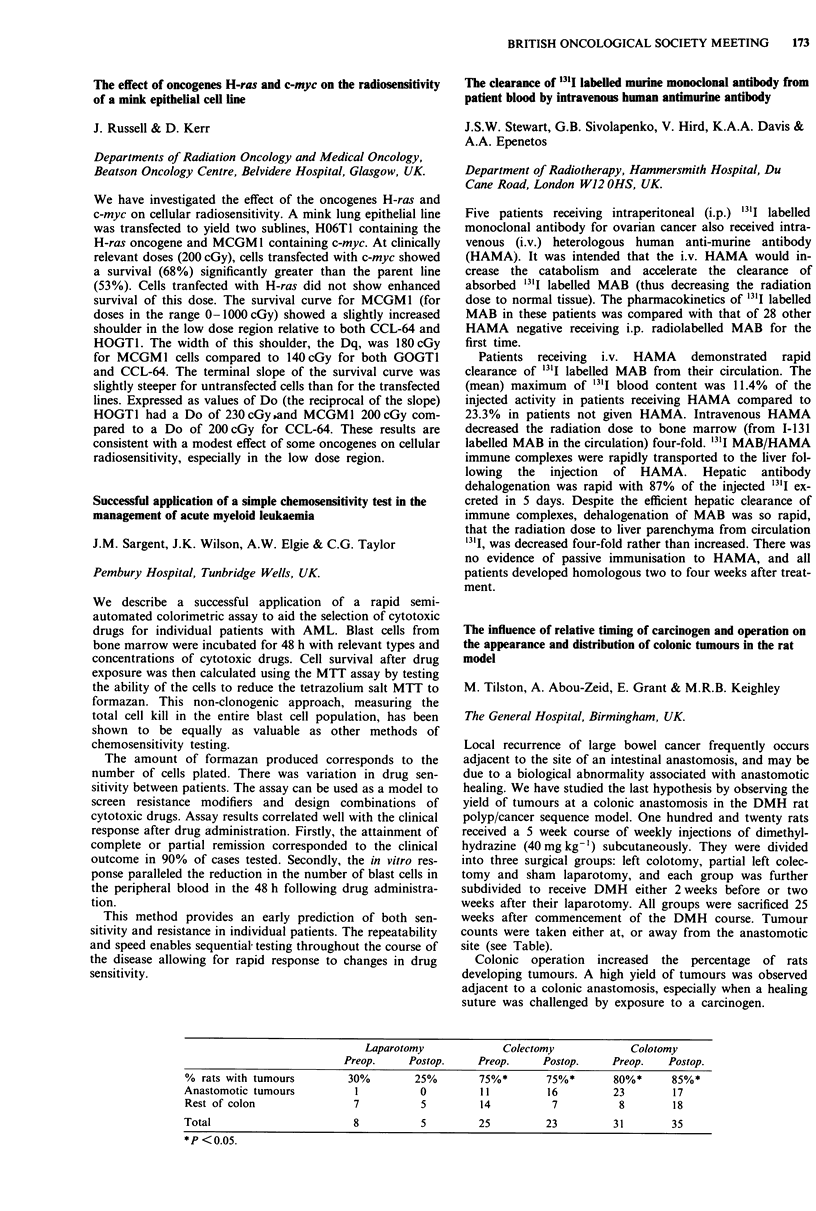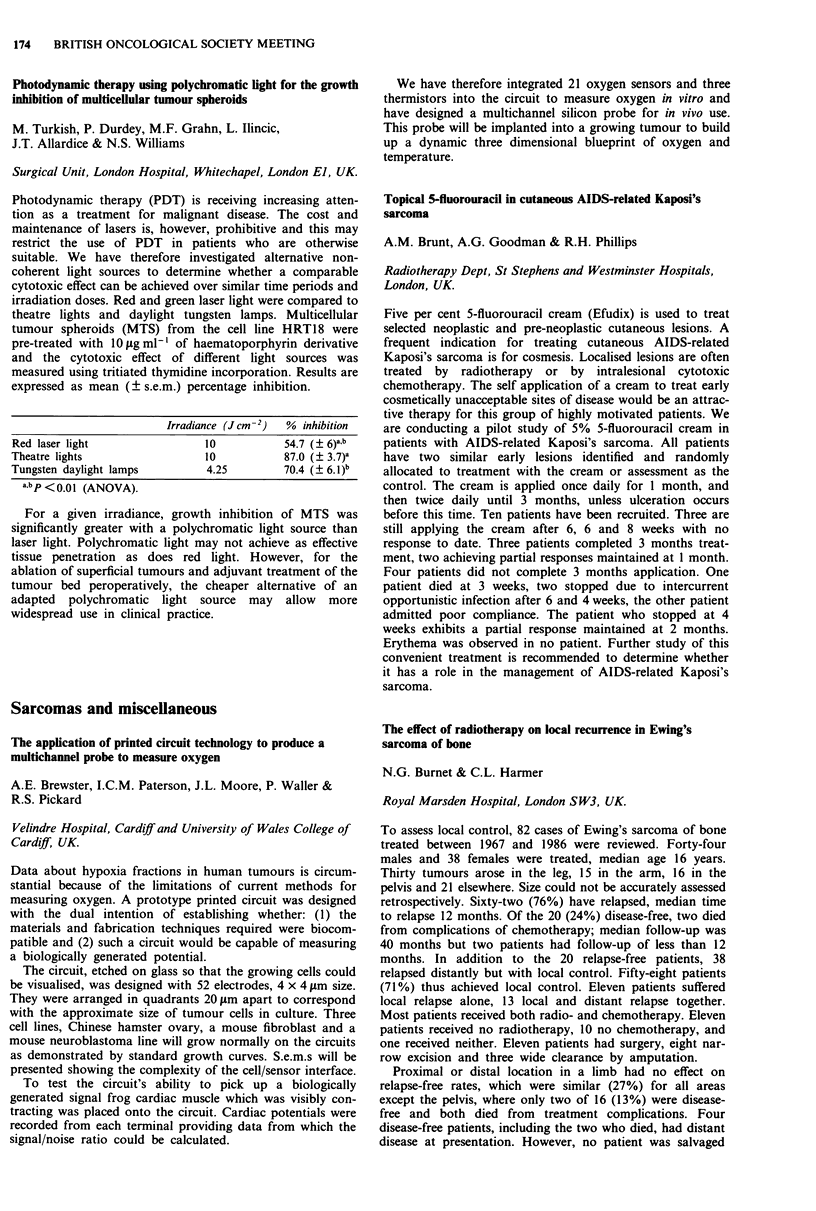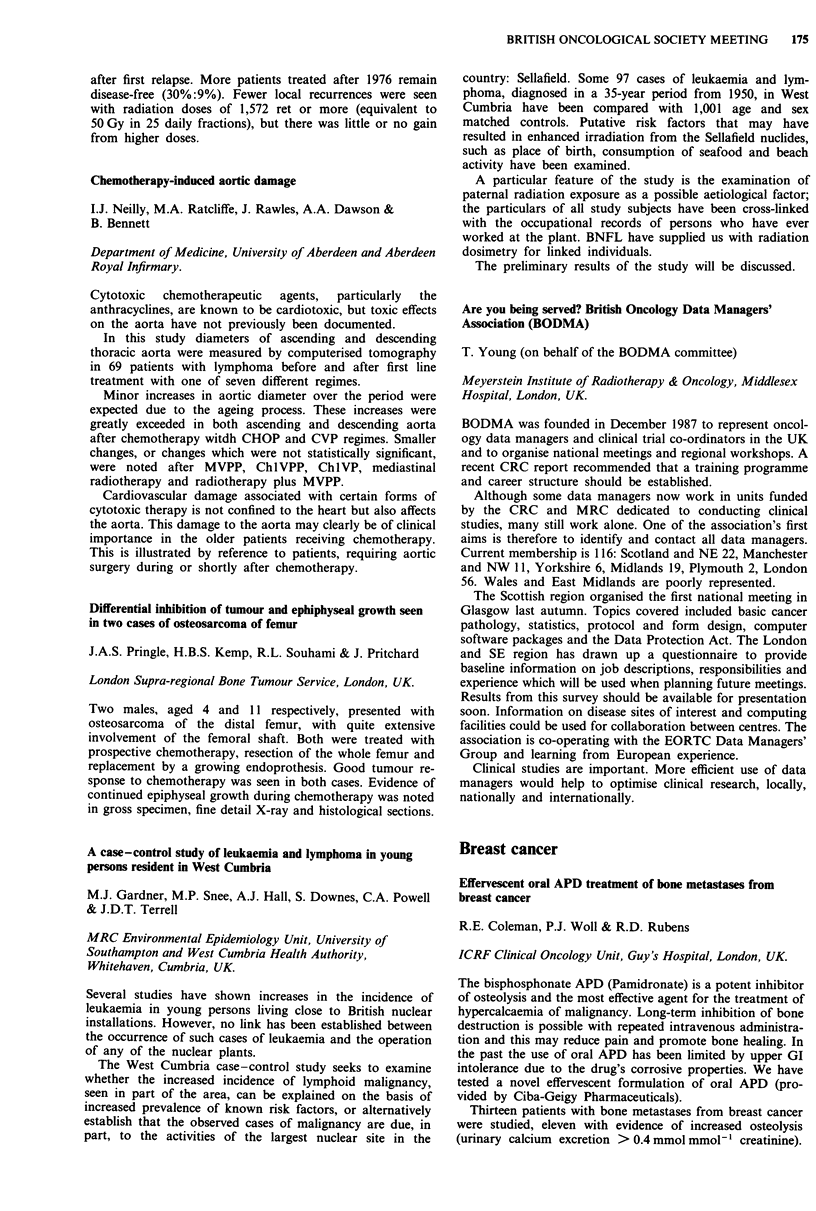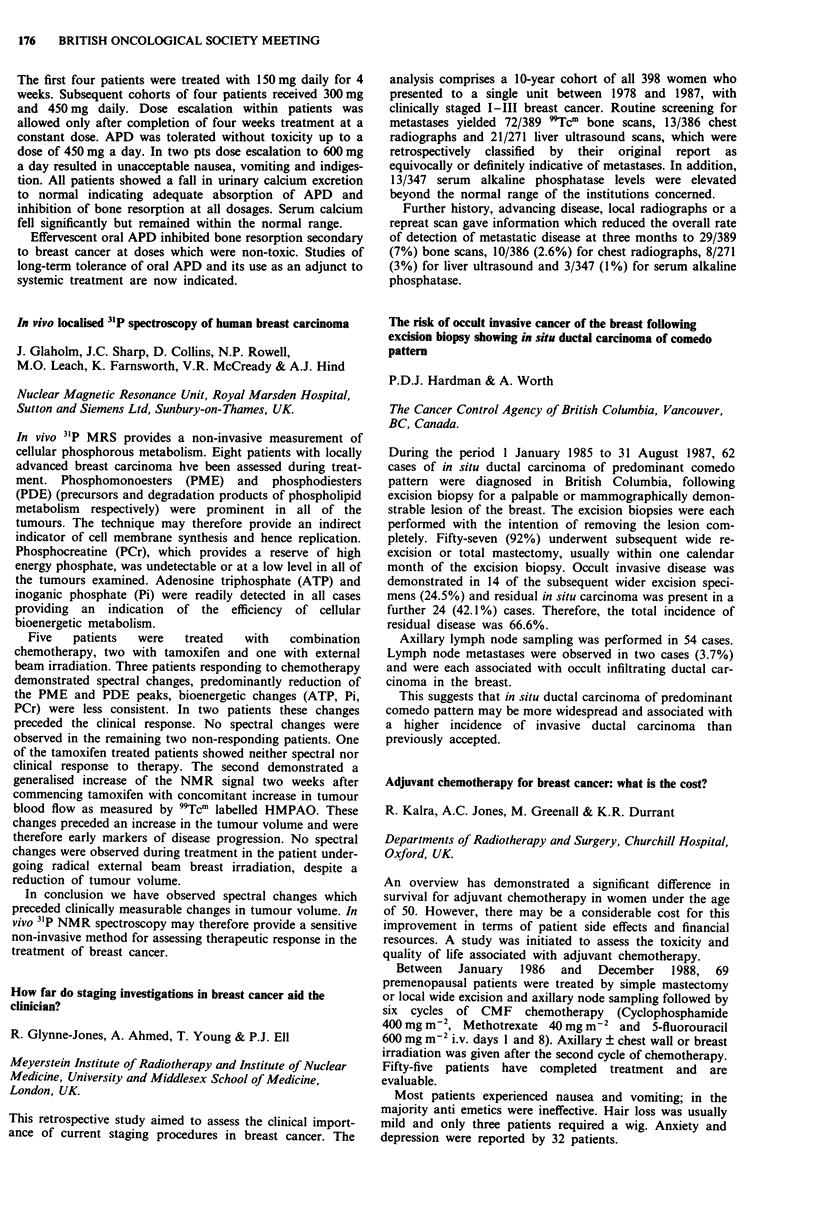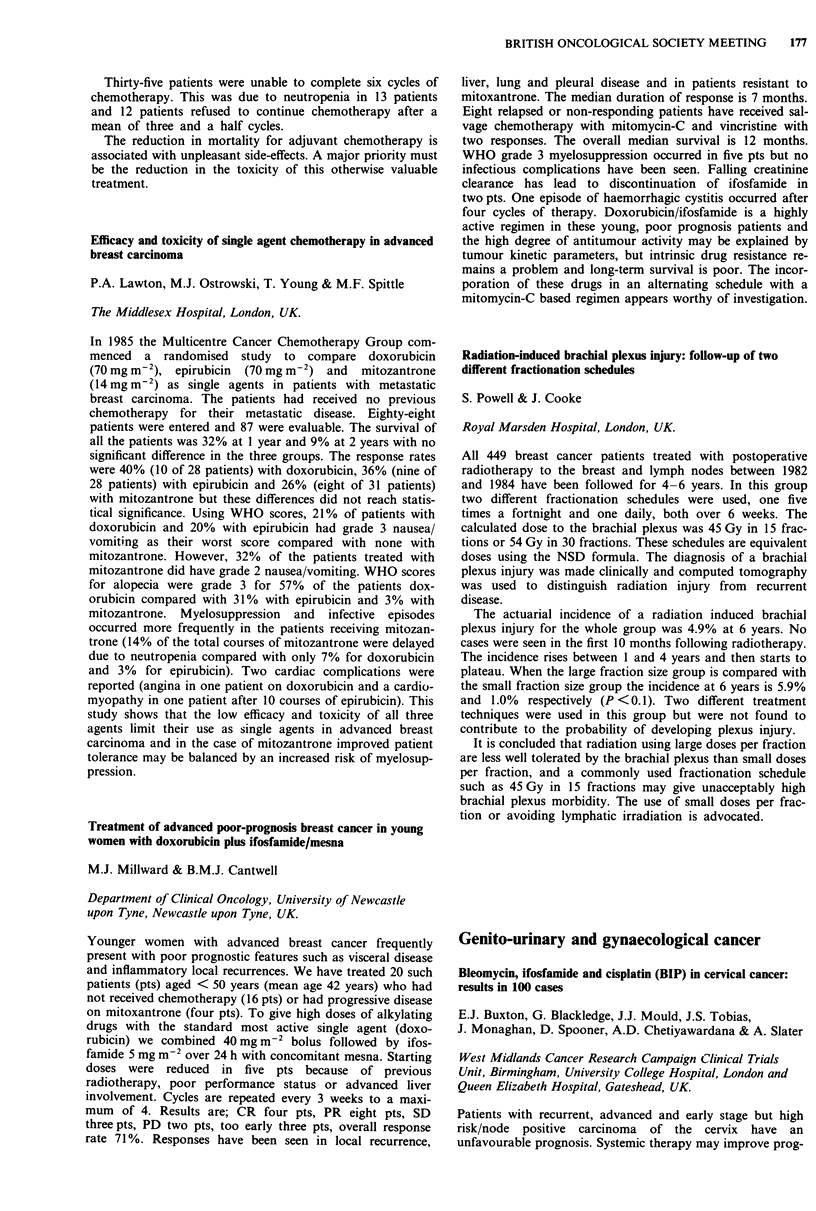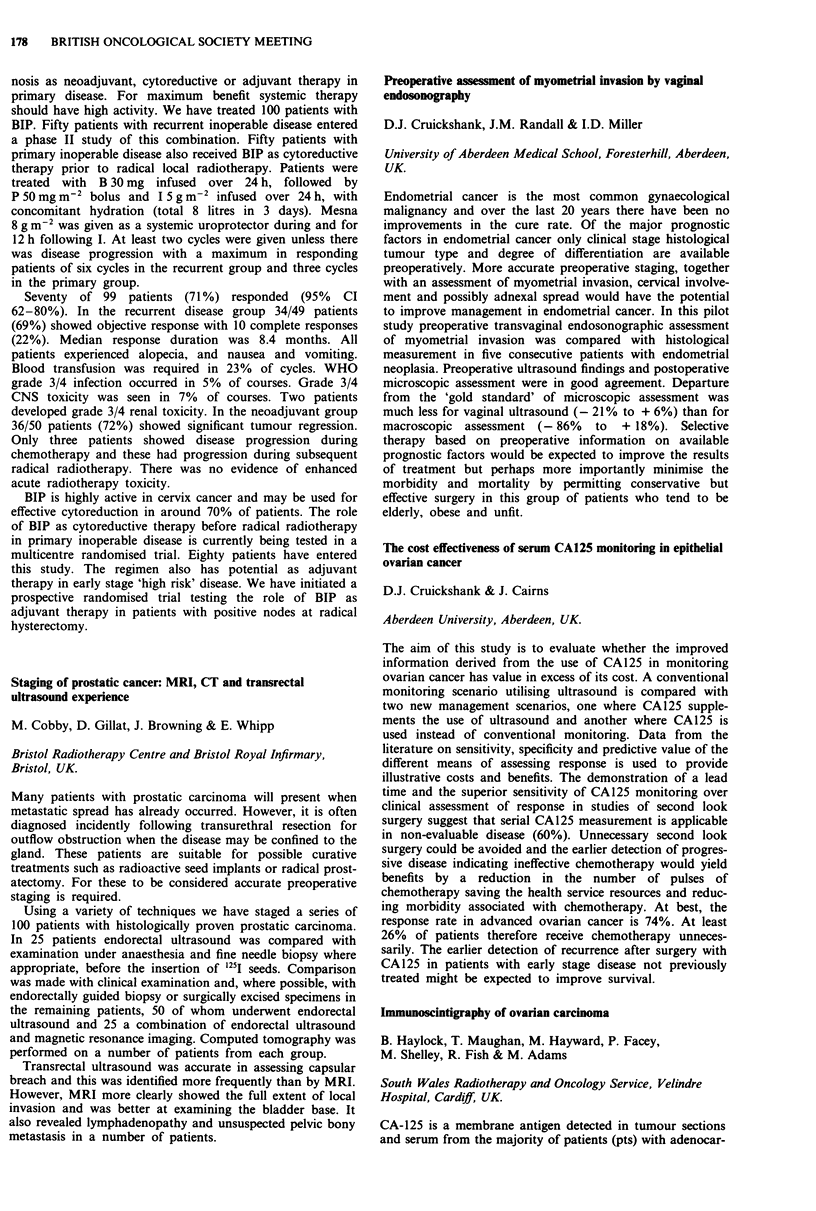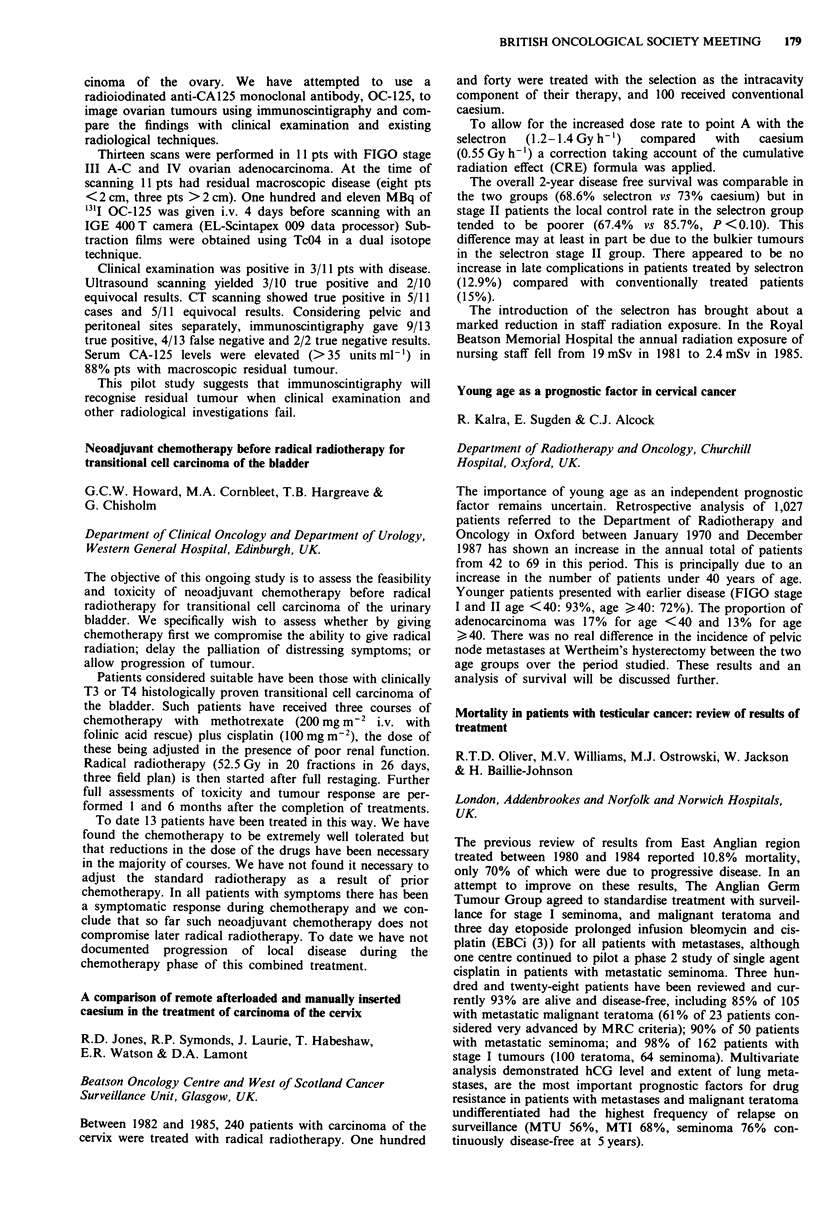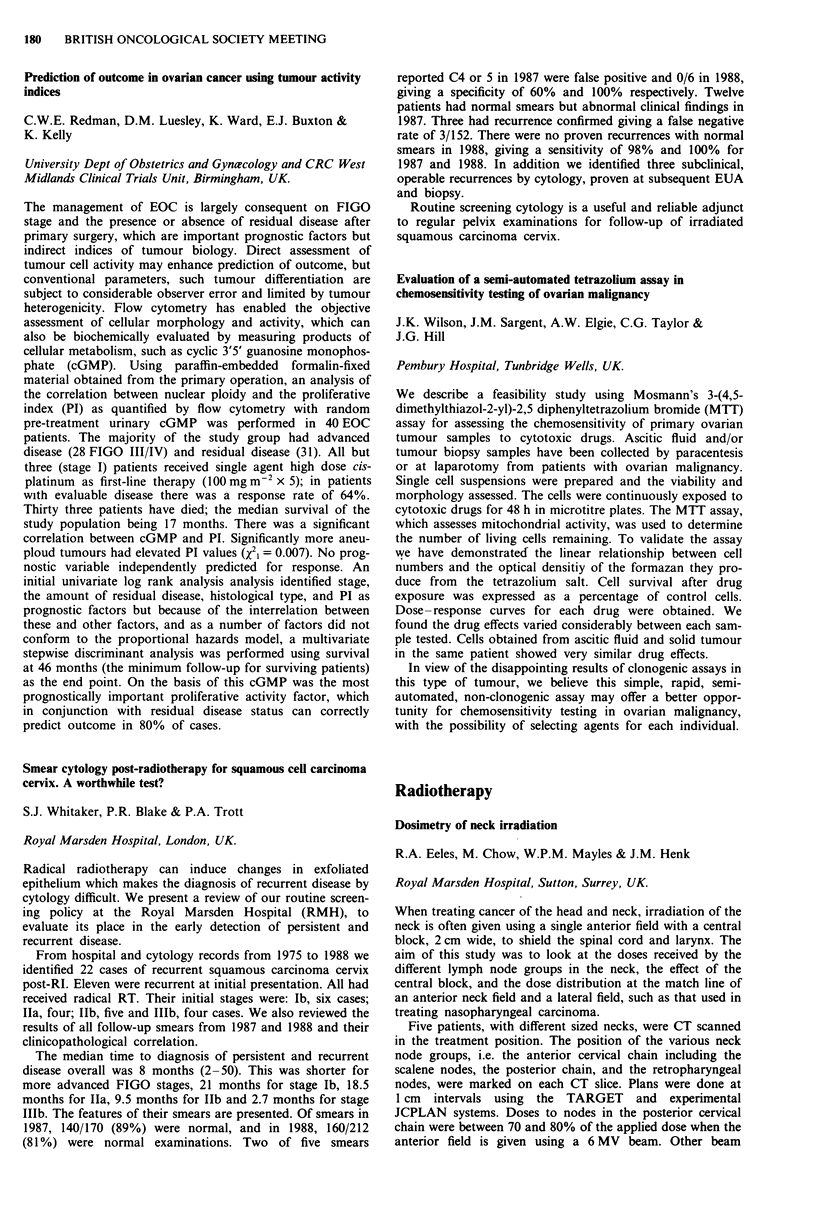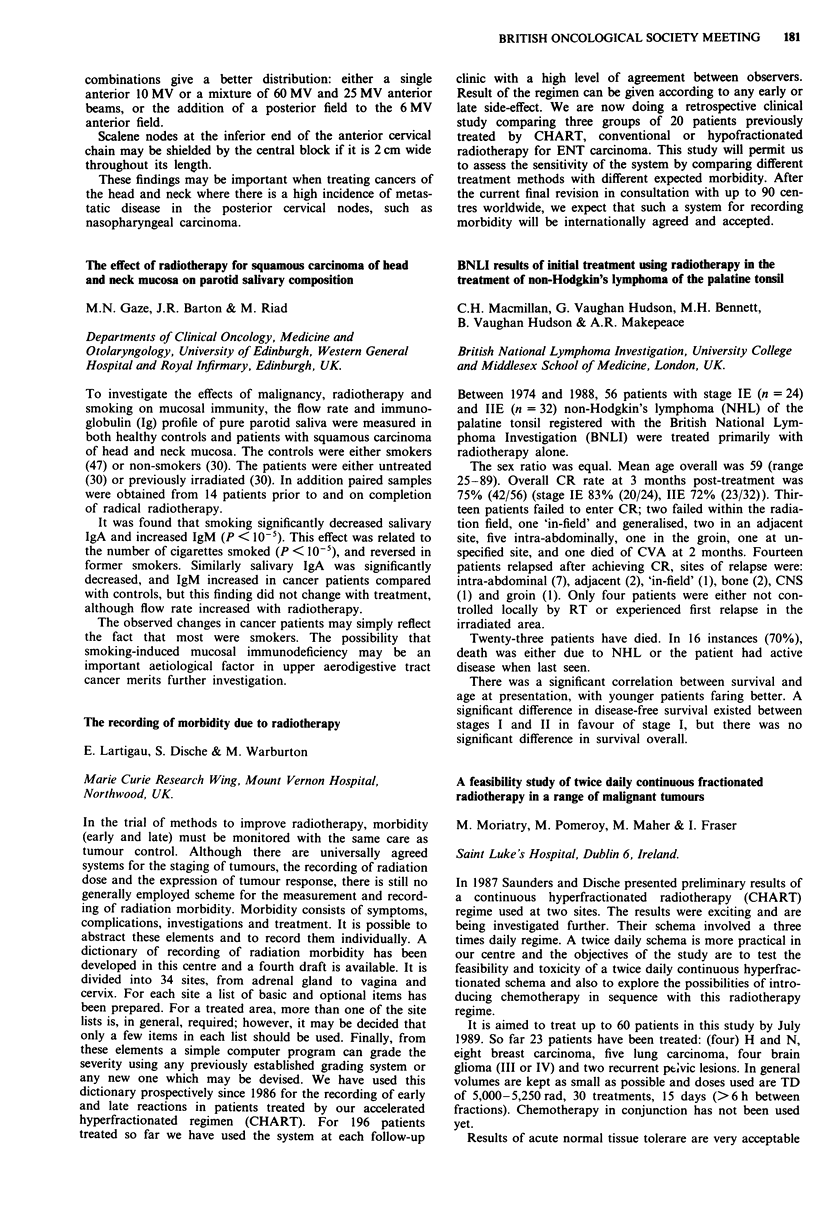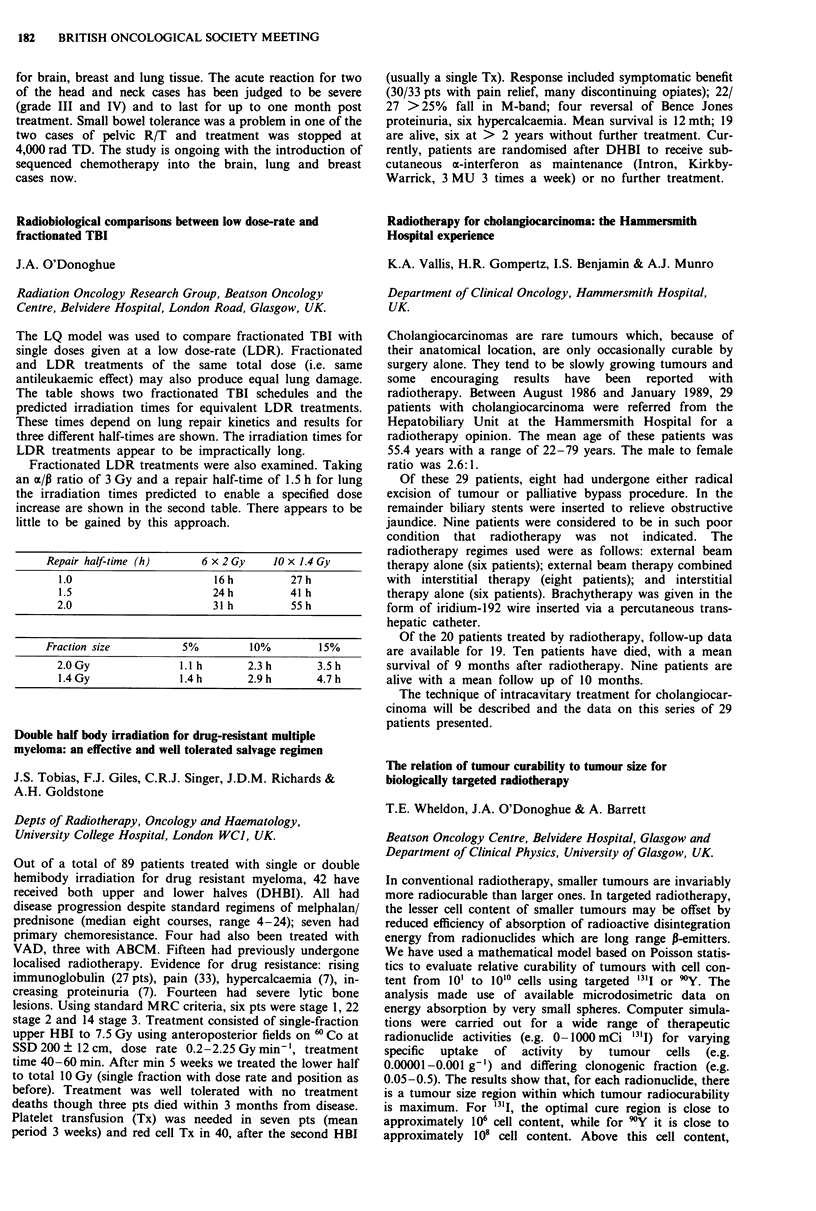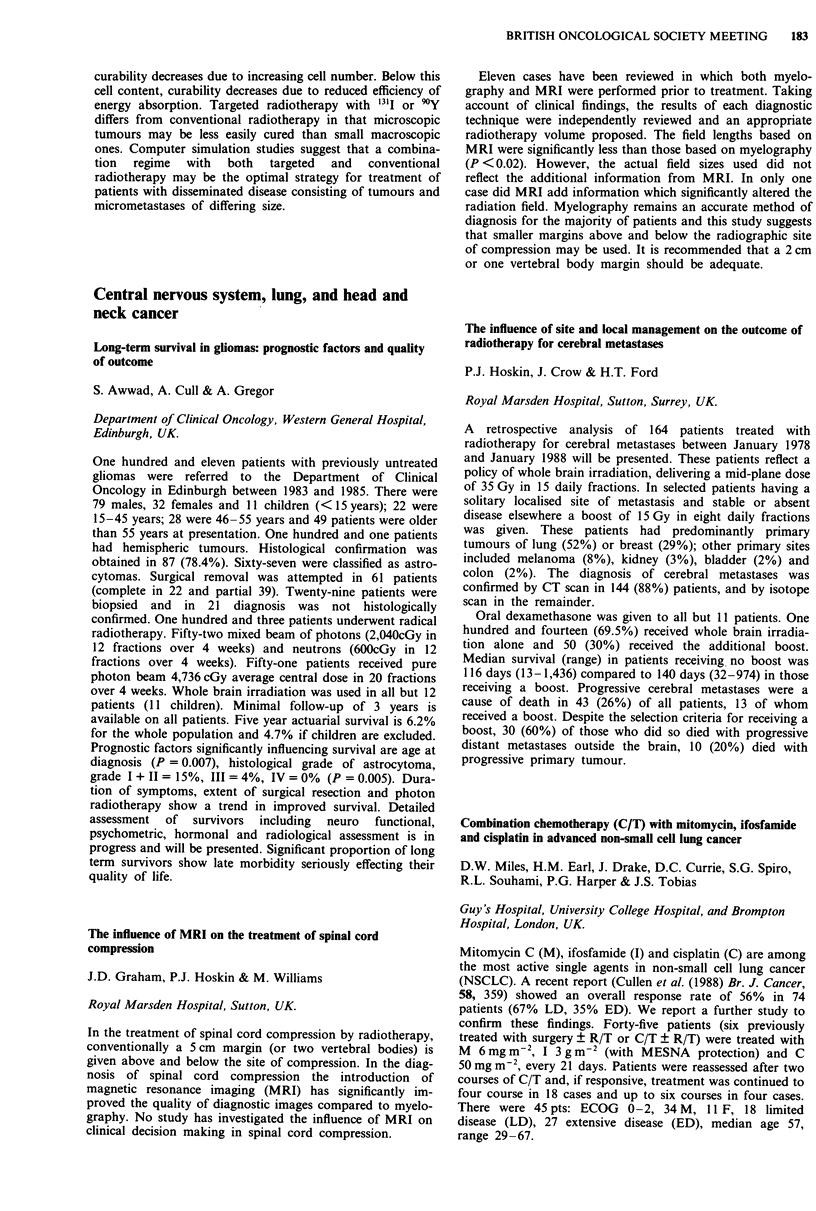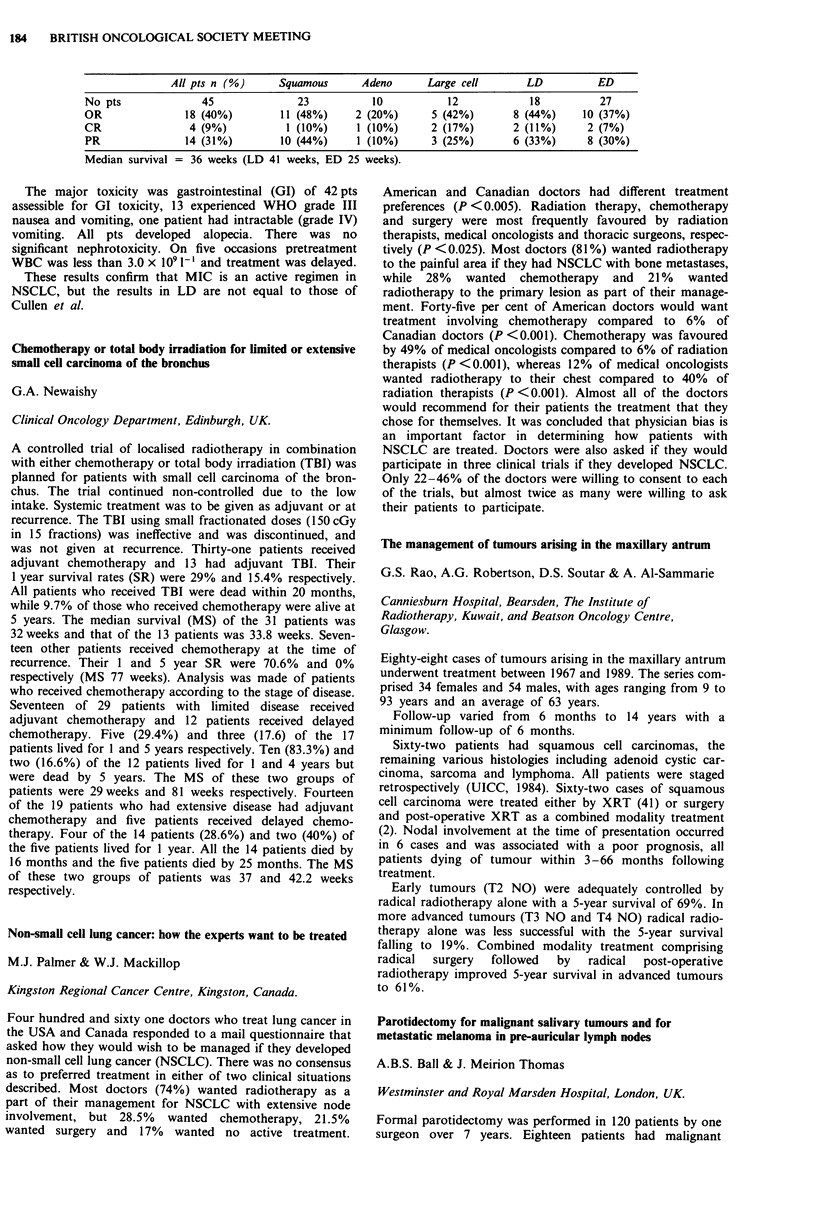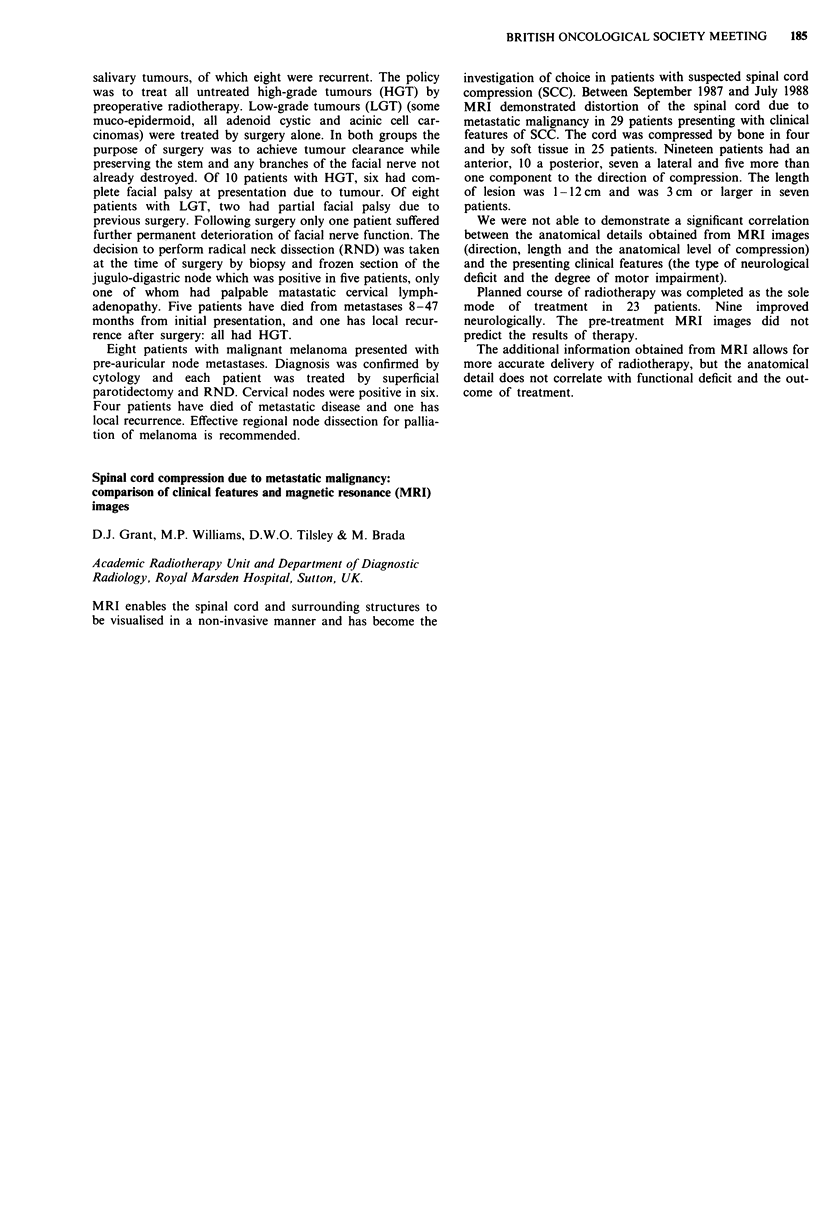# Fourth scientific meeting of the British Oncological Association. 25-27 June 1989, Edinburgh, U.K.

**DOI:** 10.1038/bjc.1990.32

**Published:** 1990-01

**Authors:** 


Br. J. Cancer (1990), 61, 156-185 © Macmillan Press Ltd., 1990

### Fourth Scientific Meeting of the British Oncological Association, 25-27 June 1989

*University of Edinburgh, Holyrood Park Road, Edinburgh, UK.*

### British Oncological Association Lecture

### Small cell lung cancer: new discoveries and future directions

R.L. Souhami

*Department of Oncology, University College and Middlesex School of Medicine, Middlesex Hospital, London W1P 7PN, UK.*

A recent UK survey has shown that with current treatments only 2.6% of patients with small cell lung cancer (SCLC) will survive 6 years. Death from SCLC does not occur after this time. This result indicates both that cure is possible and that new approaches to treatment will be necessary. For patients in the most favourable prognostic categories more intensive chemotherapy regimens (which are already capable of producing complete response rates of up to 50%) may improve results. Intensive treatments using autologous bone marrow transplantation have, so far, been disappointing. Weekly intensive regimens can be given safely and give very high response rates.

Even when chemoresistant, SCLC remains radiosensitive. Systemic radiation may be a useful means of consolidating treatment in patients responding completely to chemotherapy. As a result of international collaboration, membrane antigens have been defined on SCLC which are potential candidates for radiolabelled antibody targeting. The most immunodominant has been designated cluster 1 and is now known to be the neural cell adhesion molecule N-CAM.

The growth of SCLC can also be slowed by blocking the autocrine growth stimulatory effect of gastric-releasing peptide (GRP). GRP is one of a family of molecules which bind to SCLC surface membrane and whose actions may be interrupted by antagonists binding to specific receptors.

### Conference Lecture

### Biological and clinical implications of physical dose optimisation in radiotherapy

H. Bartelink

*The Netherlands Cancer Institute, Amsterdam, The Netherlands*

Recent developments in radiotherapy, such as multileaf collimators, three-dimensional treatment planning systems or high energy electron irradiation, have led to a new era in radiotherapy. These developments allow administration of higher doses at primary tumour sites with equal or even lower radiation doses in the surrounding normal tissues. It is hoped that these efforts of improving the treatment procedure will lead to an increase in cure rate. New forms of therapy, however, always carry with them the risk of serious complications. A large increase of the complication rate can occur due to small errors as the dose-effect curves for normal tissue complications are much steeper than those for tumour. New methods for patients' daily set-up and control procedures have to be introduced in the clinic to ensure that the intended more precise radiation therapy is carried out according to treatment plan. For selection of the optimal treatment plan new criteria have to be developed to estimate the gain and the risk of new ways of treatments. These selection criteria for choosing the optimal treatment plan should be based upon alpha/beta values for tumours and normal tissues in humans. Even proliferation rates of normal tissues and tumours have to be known and should be measured in order to design new treatment regimens. Prospective studies and prospective clinical trials with different radiation doses have to be carried out to estimate the alpha/beta values. For the proliferation rate the IUDR method is a novel approach to measure the potential doubling time. An almost complete uncertainty exists on the influence of irradiation volume on the organ tolerance. Ongoing studies on tolerance of lung and small bowel in relation to dose and volume will provide data on normal tissue toxicity. These data on normal tissue tolerance with regard to dose fractionation schedule and volume effect have to be incorporated in treatment planning systems to provide a reasonable estimate of the risk benefit ratio of new methods of treatment. The results of physical dose optimisations are to a large extent dependent on the quality control of the radiation procedure. Introduction of on-line methods such as the megavoltage imaging device and *in vivo* dosimetry are assayed to control the whole treatment procedure. Only in this way will the anticipated gain of the dose optimisation be realised.

### Louise Buchanan Memorial Lecture

### Tumour cell differentiation: an important concept in clinical oncology

N.A. Wright

*Department of Histopathology, Royal Postgraduate Medical School and Imperial Cancer Research Foundation Labs, London, UK.*

In recent years, the aspect of the malignant phenotype which has received most attention is growth and proliferation; the differentiation of tumour cells has, until recently, been relatively neglected.

Differentiation in malignancy can be approached on several different levels. As its simplest level, the behaviour of a tumour can often be predicted by its differentiation pattern; an example is that tubule formation in carcinoma of the colon is an independent variable in predicting metastatic behaviour. But now the factors which determine tubule formation in carcinoma of the colon are becoming recognised at the cell biological and molecular genetic level.

The malignant change itself can be regarded as a defect in differentiation: carcinomas contain terminally differentiated cells, and indeed these can be induced by modulating the growth conditions.

Both these considerations point to the conclusion that the *malignant phenotype is not immutable*, even in solid tumours, and that further understanding of the biological basis of differentiation in normal and malignant cell populations might suggest novel therapeutic procedures for modulating the malignant phenotype.

### Conference Lecture

### Early detection of prostate cancer: is it necessary, is it possible?

P. Scardino

*Professor of Urology, Baylor College of Medicine, Houston, Texas, USA.*

The prostate has become the leading site of cancer in men and prostate cancer is the second leading cause of death from cancer in men in the USA. The prevalence of prostate cancer found at autopsy in men suggests that adenocarcinoma of the prostate is the most common malignancy in human beings. Traditionally the early detection of prostate cancer in asymptomatic men has depended upon digital rectal examination (DRE). Recently, two new techniques capable of detecting prostate cancer in men with a normal digital rectal examination have become available: measurement of prostate specific antigen (PSA) and transrectal ultrasonography (TRUS). Yet the prospect of using these techniques in widespread screening or early detection programmes has generated considerable controversy. Both the efficacy of these techniques and the necessity for early detection of this slow-growing cancer have been seriously questioned. Because of the remarkably high prevalence of cancer in the prostate found at autopsy of men who die of other causes, the slow progression rate of the tumour, and the advanced age of men diagnosed with the disease, patients with prostate cancer are often said to be more likely to die *with* rather than *of* their disease. On the other hand, once prostate cancer is diagnosed – whether by digital rectal examination, the pathological examination of the tissue from transurethral resection of the prostate (TURP), or the development of symptoms – treatment has been generally unsuccessful in controlling the disease except in its very early stages. With 28,500 men dying of the disease in the USA this year, the need for earlier detection seems evident.

This paper will review: (1) the results of treatment trials for early stage prostate cancer; (2) the natural history of the disease; and (3) the features of 'clinical' and 'autopsy' prostate cancer, all of which together provide a rational basis for the early detection that is essential if the mortality rate from prostate cancer is to decline.

### Bob Champion Cancer Trust Lecture

### Dose effect with bone marrow transplantation in lymphomas and solid tumour

T. Philip

*Centre Leon Berard, Bone Marrow Transplant Department, Lyon Cedex 08, France.*

Autologous bone marrow transplantation is a tool to increase the dose-effect relationship in cancer therapy. In lymphomas, BMT is able to cure 70–75% of patients in partial response after induction therapy and 40% of patients still sensitive to rescue protocols at time of relapses. Primary refractory patients and resistant relapse patients are not curable yet. Addition of interleukin-2 post BMT could be a discussion to improve results for these patients.

In solid tumours encouraging results have been reported for neuroblastoma in children and testicular cancer in adults. However, ABMT is still an experimental procedure in this field and future directions will be reviewed.

### Oral presentations

### New approaches in oncology

### Neutron therapy for squamous cell carcinoma of the head and neck: the 5-year follow-up of the Edinburgh randomised clinical trial

R.H. MacDougall, J.A. Orr & G.R. Kerr

*Department of Clinical Oncology, University of Edinburgh, UK.*

One hundred and sixty-eight patients were recruited into a randomised trial of d(15) + Be fast neutron therapy compared with 4MV photons in the treatment of locally advanced squamous cell carcinoma of the head and neck. Results with a minimum of two years' follow up were reported in 1987 (Duncan *et al.* (1987), *Int. J. Radiat. Oncol. Biol. Phys.*, **13,** 171).

In May 1989 five-year follow-up became available for all patients. The five-year survival rate for neutrons is 23.5% and for photons 33.8% ($P = 0.085$). The disease-free survival for neutrons is 18.8% and for photons 30%.

Late grade 4/5 morbidity associated with neutrons exceeded that of photons.

### Excision, interstitial $^{192}$Ir and myocutaneous flap repair for the salvage of patients with head and neck cancer developing fixed nodal recurrence in the irradiated and dissected neck

M.H. Robinson, J.M. Henk, N. Stafford & P. Rhys Evans

*Head and Neck Unit, Royal Marsden Hospital, London, UK.*

Relapse of head an neck cancer with a fixed node mass following a neck dissection and radical radiotherapy is associated with a very poor prognosis. Where complete surgical clearance is unattainable these patients have been regarded as unsalvageable. We report the results of treatment of 27 such patients, median age 61.5 years. In an effort to improve these results 17/27 patients were treated with a combination of surgical resection, interstitial $^{192}$Ir implant and repair of the defect by a myocutaneous flap. Six had been treated by surgery and implant without resurfacing, and four with implant alone. Seven patients had a laryngeal primary, six oral cavity, two oropharyngeal, two hypopharyngeal and 10 other sites. The median interval between initial irradiation and the implant was 16 months and median total dose received by the 27 patients was 102.8 Gy (range 89–128 Gy). Five of six patients treated without resurfacing developed soft tissue necrosis, most healing spontaneously. The median implant dose used was 43.4 Gy (range 30–60 Gy) but since no complications have developed where a myocutaneous flap was used an implant dose of 50 Gy is now routine. The four patients not having surgery had persistent disease after treatment: one whose tumour was only debulked relapsed at the implant site and in the other neck. Of the remaining patients seven remained disease-free, four developed metastatses, two relapsed at the implant site alone, six outside the implant site and one at both sites. The median survival and disease free period of those having surgery are 11.25 months and 10.7 months respectively. The use of a myocutaneous flap and $^{192}$Ir afterloading following excision is a potentially valuable method of treating these difficult problems.

### High dose melphalan improves the prognosis in advanced neuroblastoma: results of a randomised study of 'ENSG – 1'

J. Pritchard, S. Germond, D.H. Jones & J. de Kraker

*European Neuroblastoma Study Group.*

We wished to investigate the value of high dose melphalan (HDM) as 'consolidation therapy' in children with advanced neuroblastoma. One hundred and thirty patients over 6 months of age with Evans stage III or IV tumours, consecutively referred to 16 centres, were given 3–4 weekly 'OPEC' induction therapy and, where possible, had the primary tumour resected. Ninety patients (69.2%) achieved complete response or good partial response (disappearance of all evidence of secondary deposits and shrinkage of primary by $>50\%$ in each of three dimensions), and were eligible, after stratification by stage and centre, for randomisation either to one high dose of melphalan (180 mg $m^{-2}$) with autologous bone marrow rescue or to no further treatment. Because of non-compliance by some parents and physicians only 65 patients were actually randomised. Despite serious toxicity including two treatment-related deaths, patients in the HDM group had better event-free survival (median 24 *vs* 8 months; 2-sided $P = 0.045$, log rank) than those in the 'no further treatment' group. Results were similar for children with the worst prognostic features, i.e. those with stage IV disease aged over 12 months at diagnosis (disease-free survival median 22 *vs* 5 months; two-sided $P = 0.025$, log rank). Though longer follow-up is needed to establish the pattern of long-term survival we conclude that HDM is of benefit to children with advanced neuroblastoma who have achieved complete or good partial response after OPEC and surgery.

### Hydralazine increases blood flow through human lung tumours

N.P. Rowell, B. Cronin, V.R. McCready & A. Horwich

*The Royal Marsden Hospital, Sutton and MRC Radiobiology Unit, Harwell, UK.*

Hydralazine has been shown both to reduce tumour blood flow and to potentiate the cytotoxicity of melphalan and the bifunctional nitroimidazole RSU 1069 in mice. In order to determine whether such a strategy has clinical potential, the effects of hydralazine on blood flow through human tumours were investigated in fifteen patients with carcinoma of the bronchus (mean age 67; range 52–75 years) who received a single oral dose of hydralazine in the range 0.37–2.26 mg $kg^{-1}$ according to age, acetylator status and an escalating dose schedule. Tumour blood flow was assessed by single photon emission computed tomography (SPECT) performed ten minutes following intravenous $^{99}Tc^{m}$-HMPAO on two occasions 4–8 days apart, the second being performed 60 min after hydralazine. Blood pressure and cardiac output were measured non-invasively and repeatedly throughout.

Hydralazine caused a 25% increase in tumour blood flow ($P = 0.065$), with a trend for greater increases to occur in patients sustaining greater falls in peripheral resistance. Tumour vascular resistance (calculated from blood pressure and estimates of blood flow) fell, indicating active vasodilation in arterioles supplying tumours. Common symptoms in higher doses were giddiness, palpitations and facial flushing. Doses greater than 1.5–2.0 mg $kg^{-1}$ were poorly tolerated. Allowing for extensive first-pass metabolism in the liver, these doses are equivalent to only a fraction of those used in animal studies.

### A preliminary report of treatment for in-transit metastatic melanoma with a carbon dioxide laser

J.M. Gattuso, R. Waters & J. Meirion Thomas

*Westminster Hospital, London, UK.*

Six patients (aged 60–79 years) with a total of 116 lesions on the legs were treated with a carbon dioxide laser. Three patients had recurrent disease following isolated limb perfusion and the other three were treated by laser in preference to isolated limb perfusion. Two patients were treated for the second time for new lesions. General anaesthetic was used initially as the level of anaesthetic required was unknown. Subsequently two treatments were done under local anaesthetic. Operating time varied with the size and number of the lesions treated. For example, in case 1, 40 lesions, 1.5–16 mm diameter, were treated in 30 min. Postoperative pain in all cases was absent or minimal, and if present was always relieved by simple oral analgesics. Wounds were not sutured and within 24–48 h a dry eschar developed. Therefore only simple dry dressings were necessary and minimal nursing care was required. No patient developed infection, cellulitis or needed antibiotics. Patients could be discharged home the next day and in future day-case treatment will be considered suitable. Patient follow-up is still continuing, however it does appear that the laser treatment is locally curative. Further recurrences have always occurred at new sites. In conclusion, laser treatment has several advantages over conventional treatment. It offers rapid and effective treatment for multiple recurrences, it can be used in the outpatient clinic under local anaesthetic and minimal post-operative care is required. In selected patients the technique is likely to complement or replace isolated limb perfusion.

### Decision making in oncology

### Early gastric cancer detection: an achievable aim

M.T. Hallissey, A. Jewkes, J.W.L., Fielding, D.J. Ellis & W.H. Allum

*Department of Surgery, Queen Elizabeth Hospital, Birmingham, UK.*

The Japanese have demonstrated that the detection of early and curable gastric cancer is an achievable aim. Despite the advances in the diagnostic tools avilable, the West has seen little change in the proportion of operable cases in the past 30 years. While mass radiographic screening is impractical in the UK, the identification of a high risk group may allow a suitable programme of early detection to be developed. Dyspeptic patients over the age of 40 form such a group, in whom early investigation may increase the detection of curable gastric cancer.

A study was established with six general practices in central Birmingham in 1984 and a further four in Sandwell in 1986. The aim was to provide an endoscopic diagnosis in all dyspeptic patients aged over 40 within 3 weeks of attending surgery. To the end of 1988, 2,703 patients have been entered of whom 77% had identifiable pathology. There were 107 malignancies diagnosed of which 53 were gastric carcinomas. Of the gastric cancers, 21.4% were stage I, 12.5% stage II and 28.6% stage III. Over the first 2 years in central Birmingham, there has been a significant change in stage distribution and increase in survival to 2 years in the patients from the study practices.

The study has demonstrated that the early investigation of dyspeptic patients aged over 40 can increase the rate of early gastric cancer detection to 26.8% and improve mortality from the disease.

### A survey of the management of breast cancer and attitudes to it in one health district

M. Leslie, E.J. Maher, B. Shorey & J. Ashby

*Regional Centre for Radiotherapy, Mount Vernon Hospital, Northwood, Middlesex, Hillingdon Hospital and Brunel University, UK.*

Hillingdon District contains a population of 240,000. In a year there are 100 new cases of breast cancer and 50 deaths. The number of cases at any one time is 700. During the period January 1985 to December 1986 there were 199 new cases of breast cancer. The five surgeons in the district shared the workload and the numbers seen by each were 58, 46, 37, 32 and 26. The percentage undergoing mastectomy was 44%. Of the 56% treated by conservative surgery 14.8% had an axillary staging procedure. Five per cent received adjuvant chemotherapy. The proportion receiving adjuvant hormonal therapy was not determined. No patient was in a clinical trial. There was no local consensus with regard to treatment.

Sixty-seven of the district's 121 GPs and 33 of 80 community nurses replied to a questionnaire regarding breast cancer management. With regard to the aims of local therapy 20% of GPs held the view that it was to increase survival, 45% that it was to increase disease-free survival and 30% that it decreased local recurrence. With regard to follow up a majority of both GPs (67%) and community nurses (60%) felt the aim was early detection of metastases. Views on the side-effects of radiotherapy and chemotherapy were also explored with some unexpected findings. In particular 17% of GPs and 24% of community nurses felt that hair loss was an expected side effect of radiotherapy treatment.

The results of this survey in one district show the lack of any local consensus or recognised 'specialist' surgeon and point to some misunderstandings amongst community nurses and GPs about aims and side effects of breast cancer treatment.

### Factors influencing clinical decision-making in the treatment of advanced head and neck cancer

E.J. Maher & A. Jefferis

*Regional Centre for Radiotherapy and Oncology, Mount Vernon Hospital, Northwood, Middlesex and Wexham Park Hospital, Slough, UK.*

Twenty radiotherapists and 20 surgeons were given the case histories of three patients with advanced head and neck cancer, including a man of 72 with pyriform fossa carcinoma and bilateral nodes (1), a man of 42 with carcinoma of the tongue and bilateral nodes (2) and a woman of 62 with recurrent tonsillar carcinoma and contraleral nodes (3). They were asked how they would manage them and what factors influenced them to change their approach. Radical treatment was proposed by 13 in case 1, 37 in case 2 and 23 in case 3 (39 for surgeons *vs* 34 for radiotherapists). As expected, type of radical therapy varied. Both surgeons and radiotherapists favoured radiotherapy (37 *vs* 34), while surgeons used more surgery (37 *vs* 20) and less chemotherapy (17 *vs* 9) as part of radical treatment.

Non-TNM factors influencing a change in approach included age, general condition, social support and persistent smoking or drinking, e.g. while social support was very influential for 30% of radiotherapists, 50% would not take it into account at all in selecting for radical treatment; similarly, 'absolute cut-offs' for radical treatment in case 1 ranged from 75 to 90 years of age.

Selection for radical treatment in advanced cancer of the head and neck involves more than TNM stage and Karnofsky status. Subtle differences in selection criteria may significantly bias the results of published series and clinical trials. The use of surrogate studies, together with a national data base may clarify the decision-making process.

### Lung cancer patients: perception of their illness and its treatment

L. Ginsberg, C. Quirt, A.E. Ginsberg & W.J. Mackillop

*Kingston Regional Cancer Centre, Kingston, Ontario, K7L 2V7, Canada.*

Forty-five patients with primary lung cancer were interviewed within 3 months of their diagnosis to determine how they perceived their illness and how their perceptions compared with those of their attending physicians. All of the 45 patients knew that they had lung cancer. Twenty-six of the 45 correctly recognised the extent of their disease as localised, regional or metastatic. However, 16 of 25 patients with regional disease, and two of the 15 patients with metastatic disease thought that the cancer was localised. Two of the 11 patients being treated with curative intent thought that the treatment was palliative only, and nine of the 34 patients being treated palliatively believed the treatment intent was cure. Seventeen of the 34 incurable patients thought that they had a chance of being cured and 15 of these thought that the probability of being cured was at least 50%. In every case the doctors stated that they had accurately informed the patient about the probability of cure and that they thought the patient accurately understood the prognosis. No significant association was demonstrated between the accuracy of the patient's perception of his or her situation and anxiety and depression scores measured by the Speilberger and Beck self evaluation instruments respectively.

It was concluded that while most patients with lung cancer today recognised their diagnosis, many failed to appreciate the gravity of their situation and grossly over estimated the potential benefits of treatment which they are receiving.

### How good are physicians at judging life quality and physical performance in incurable non-small cell lung cancer (NSCLC) patients?

J. Regan, P. Jones, N. Cooke & J. Yarnold

*Academic Radiotherapy Unit, The Royal Marsden Hospital, Sutton, Academic Medical Unit, St George's Hospital, Tooting and Department of Medicine, St Helier Hospital, Carshalton, UK.*

Forty patients with NSCLC were treated according to the recent MRC palliative radiotherapy protocol with 20 patients randomised to 30 Gy in 10 daily fractions and 20 patients to 17 Gy in two fractions one week apart. The physicians assessed patient general condition, activity and respiratory status according to the MRC protocol. Forced vital capacity (FVC), haemoglobin and weight were also measured. The physicians were blind to the results of these measures at the time of assessment. Patients also completed the EORTC lung cancer quality of life questionnaire.

The physician assessment of patient general condition correlated well with the FVC ($R = 0.64$, $P < 0.0001$). The patient self-assessment of their physical condition also correlated with the FVC ($R = 0.45$, $P = 0.009$). Physician score and patient self-assessment of physical condition also correlated ($R = 0.47$, $P = 0.002$). In contrast, patient global asssessment of life quality did not correlate with any of these measures ($P > 0.05$).

In conclusion, doctors and patients agree in their assessment of physical condition but the doctors appear unable to judge life quality as assessed by the patients themselves

### Can oncologists predict the course of malignant disease in their individual patients?

C.F. Quirt, W.E. Stewart, A.D. Ginsburg & W.J. Mackillop

*Kingston Regional Cancer Centre, Kingston, Ontario, Canada.*

Although information about prognosis and tumor response is readily available for *groups* and *subgroups* of patients with the common malignant diseases, there is no information about oncologists' ability to predict what will become of *individual patients*. We prospectively obtained attending physicians' estimates of probability of cure for 98 patients undergoing treatment at the Kingston Cancer Clinic. These predictions were subsequently correlated with the outcome of the disease process. The two-year survival rate of 56 patients undergoing curative treatment, and of 42 patients undergoing palliative treatment were 66.9% and 11.4% respectively ($P < 0.0001$). The two-year disease free survival of a subgroup of 28 patients, whose predicted cure rate was $> 0\% \leqslant 50\%$, was 35.8% compared to 85.6% for a subgroup of 28 patients whose predicted cure rate was $> 50\%$ ($P < 0.0001$). There was a significant correlation between predicted probability of cure and complete response (CR) rates (predicted cure rate $> 0 \leqslant 30\%$, CR = 62.5% and predicted cure rate $> 30\%$, CR = 100%; $P < 0.01$).

Doctors estimated the survival of 39 of the 42 incurable patients as 0–3 months, 4–6 months, 7–12 months, 1–2 years or greater than 2 years. Twelve died within the predicted interval, 12 died earlier and 15 lived longer than expected. Patients expected to live for less than 6 months had a median survival of 238 days compared to 414 days for those expected to live for more than 1 year. The variance of survival within each group was very large and this difference did not reach statistical significance ($P = 0.1$). The accuracy of survival predictions was independent of the doctor's age, sex or specialty training. Oncologists' predictions of curability correlate well with both complete response rates and 2-year survival rates, but estimates of duration of survival for individual incurable patients are usually inaccurate.

### Technical radiotherapy

### Clinical applications and quality control for the Clatterbridge neutron trials

S.W. Blake, S. Myint, R.D. Errington & H.M. Warenius

*Douglas Cyclotron Unit, Clatterbridge Hospital, Bebington, UK.*

Since February 1986, the Clatterbridge cyclotron has been used to conduct clinical trials in which patients are randomised to treatment with either photons or high energy neutrons. The neutron beam is produced by bombarding a beryllium target with 62 MeV protons, and the treatment unit consists of a variable collimator mounted on a fully isocentric gantry equipped with both wedges and field shaping facilities. The depth of the 50% isodose is 16 cm for a 10 × 10 cm field which is compatible with that of an 8 MV linac. Careful quality control has been applied to both the beam parameters which affect the dose distribution and also the patient dose distributions. A review of the quality assurance results showed that the random uncertainties in calibration, field flatness, field symmetry and beam penetration contributed to a total random uncertainty of 1.5% to the tumour dose. For the first 100 patients, the comparability of neutron and photon treatment plans was one of the criteria for entry to the trials. A dose variation of ± 7% throughout the target volume was the limit of acceptability, and most plans had variations of less than ± 5%. An initial comparison of the phase 1 treatment volumes for sites in the pelvis resulted in values of approximately 3,300 $cm^3$ for both neutron and photon treated patients. It is hoped that the improved dose distribution combined with advances in the localisation of disease will lower the morbidity of neutron therapy.

### The effect of varying dose schedules in response of glottic carcinoma of the larynx

A.G. Robertson, C. Robertson, P. Boyle & R.P. Symonds

*Beatson Oncology Centre, Western Infirmary, Glasgow, UK.*

It is widely accepted that different dose–time–fractionation schedules give rise to different acute and long term reactions. Three hundred and ninety-three patients with carcinoma of the larynx confined to the glottis were treated in Glasgow between 1958 and 1977. Six different dose–time–fraction schedules were used during this period to treat these patients radically. One hundred and seventy-six patients had T1 N0 M0 disease, 82 had T2 N0 M0, and 46 had T3/T4 N0 M0. Ninety-four patients were treated using a 60 Gy, 30 fraction, 42 day schedule; 104 with a 60 Gy, 25, 35 schedule; 38 with 54 Gy, 18, 42 schedule; 14 with 56.5 Gy, 25, 35 schedule; 24 with 61 Gy, 15, 36 schedule, and 29 with 60 Gy, 30, 49 + schedule. The resulting morbidity, tumour control and hence long-term survival are presented.

For all stages patients treated with the schedule 60, 25, 35 achieved the best tumour control. Where there was a gap in treatment, i.e. those treated with the 60, 30, 49 + schedule, there was a definite reduction in tumour control. There was no obvious difference in the morbidity associated with these schedules.

Consideration of the cumulative radiation effects (CRE) (see figure for all stages of the tumour) shows that tumour control increases as CRE increases from 1,700 to 1,900 rets. Above 1,900 rets the data are not sufficiently reliable to support a continuing increase in tumour control or to conclude that there is a decrease in tumour control.

### Three-dimensional (3D) treatment planning in radiotherapy of brain tumours

A.M. Bidmead, V. Kaushal, L.J. Hill & M. Brada

*Departments of Physics and Radiotherapy, The Royal Marsden Hospital, London and Sutton, UK.*

3D treatment planning enables the calculation of integral dose-volume histograms (DVH) which give the true picture of dose distribution in normal tissue. We analysed the DVH in normal brain of eight patients undergoing localised external beam radiotherapy for CNS tumours. A mean of 43% (24-57%) of normal brain volume received more than 50% and mean of 22% (9-38%) received more than 85% of the maximum tumour dose. The addition of customised shielding blocks using a 3D planning facility led to a mean reduction of 13% and 8% of the normal brain volume irradiated to more than 50% and 85% of the maximum tumour dose respectively.

Dose-volume histograms demonstrate that surprisingly large volumes of normal brain receive clinically significant doses of radiation despite localised treatment technique. Although the use of customised shielding blocks reduces the amount of normal brain irradiated, the reduction is unlikely to be sufficient for a significant improvement in therapeutic ratio. Further improvement may only be achieved with more localised delivery of radiation.

### The cellular $\alpha/\beta$ ratio: is it a constant?

J.H. Peacock, T.J. McMillan & G.G. Steel

*Institute of Cancer Research, Sutton, Surrey, UK.*

The $\alpha/\beta$ ratio has been widely used as a measure of recovery in both normal tissues and tumours. Values of $\alpha$ and $\beta$ for tumours are usually obtained by computer-aided analysis of dose-survival curves and for human tumour cells have varied from 2.1 to 92 (e.g. Fertil & Malaise (1981) *J. Radiat. Oncol. Biol. Phys.*, **7**, 621).

We have used two measures of cellular recovery in order to derive independent measures of $\alpha$ and $\beta$ in 15 human tumour cell lines of varying sensitivity. Using low dose-rate irradiation ($\sim$ 1cGy min$^{-1}$) recovery was complete during irradiation and cell survival fell exponentially with dose as a function of $\alpha$ only ($SF = e^{-\alpha D}$).

The value of $\beta$ is related to the recovery capacity of the cells. According to the linear-quadratic equation, the split-dose recovery ratio (RR) is highly dose-dependent: $RR = \exp(2\beta d^2)$.

By performing split-dose experiments over a range of doses and plotting $\log_e$ (RR) against $2d^2$ we get a reliable value for $\beta$.

Our results show that the $\alpha/\beta$ ratio calculated from the LQ fit to the acute survival curves varied from 11 to 46. In contrast, when the values were obtained as described above, we came to the startling conclusion that the $\alpha/\beta$ ratio is constant with a value of $10.3 \pm 1.7$.

### The value of ICRU 38 dosimetric parameters in assessing pelvic tolerance to intracavitary therapy in early cervical cancer

I.H. Kunkler, A.L. Gerbaulet, G.R. Kerr & D.C. Chassagne

*Institut Gustave Roussy, Paris, France and Department of Clinical Oncology, Edinburgh, UK.*

The correlation between intracavitary (IC) dose to critical pelvic organs and complications has previously been hampered by inconsistent bladder and rectal reference points. Standardised ICRU 38 dosimetric parameters (Kerma, intracavitary volume (V) and maximum doses to bladder, rectum and lymphatic trapezoid), have been correlated with pelvic complications (PC) in 453 patients treated at IGR 1975-84 with stage I and II (proximal) ca cervix. Standard treatment with IC therapy 60 Gy, or 40 Gy (if 20 Gy by external beam (EB) before intracavitary therapy) and colpophysterectomy and external iliac lymphadenectomy (CHL). Pelvic EB (20 Gy) was given in 83 patients pre IC and postoperatively (45 Gy) in 68. PC were classified by the glossary of Chassagne. The incidence of PC was compared for different Kerma, IC volume and critical organ dose levels using $\chi^2$ and Kruskal-Wallis tests (KW).

The incidence of grade I, II and III/IV complications was 45% (200), 33% (121) and 4% (21); combined complications to the rectum 1.5% and to the bladder 3.4%. There was a significant association between bladder reference dose from IC and vesico-vaginal fistula (VVF) ($\chi^2$, $P = 0.03$) and between combined rectal complications (grades I-III/IV proctitis and rectovaginal fistula) and rectal reference dose from IC (KW $P = 0.03$). There was no association between IC dose to the lymphatic trapezoid and the incidence of lymphocele. No association was found between IC volume, or Kerma and combined rectal or bladder complications. ICRU 38 dosimetric parameters may provide a useful guide to predicting pelvic morbidity from intracavitary therapy.

### AIDS and sarcomas

### AIDS-related lymphoma: experience at St Stephens and Westminster Hospital

A.G. Goodman, A.M. Brunt, R.H. Phillips, M.S. Youle & B.G. Gazzard

*Department of Radiotherapy and Oncology, Westminster Hospital and Department of AIDS Medicine, St Stephens Hospital, London, UK.*

Between 1986 and 1988, 11 patients (all homosexual or bisexual men) with HIV-related systemic lymphoma were treated at St Stephens and Westminster Hospitals. Ten patients had non-Hodgkin's lymphoma (NHL). One had Hodgkin's disease (HD) (stage IA). He was treated with radiotherapy and survived 14 months before dying, free of disease, of an opportunistic infection (OI). All those with NHL had high grade histologies, with extranodal disease in eight patients. Two had stage I disease, four had stage IE disease (one Waldeyer's ring, two stomach, one rectum), and four had stage IV disease. CNS involvement was common, with meningeal disease at presentation in two patients and at relapse in 2 more. Unusual pain appeared to be a feature of such relapses. Radiotherapy was given for localised disease (40-50 Gy in 2 Gy daily fractions). Mucosal and skin reactions were occasionally severe as may be expected in HIV positive patients. Chemotherapy (CHOP-M or PACE-BOM) was given in standard doses for generalised disease. There was one infective death (TB) on treatment. Overall survival has been short, with a median of 5 months. Three deaths were due to disease, three to OI and one to Kaposi's sarcoma (KS). There are two long-term, disease-free survivors, at 36 months (stage IV) and 12 months (stage I). Two others are alive at less than 6 months, one in complete remission (CR) and one in partial remission (PR) on chemotherapy. Further studies are required to elucidate which patients are likely to benefit from standard intensive treatment of such lymphomas.

### The CD4 molecule: its role in therapy and vaccine strategies in AIDS

A.G. Dalgleish, D. Wilks, L. Walker & J. Habeshaw

*MRC Clinical Research Centre, Harrow, Middlesex, UK.*

The CD4 molecule binds to all isolates of HIV at an affinity in excess of $10^9$ mol. Whereas the rest of the envelope is highly variable and elicits antibody responses which are isolates specific, loss of the ability to bind to the CD4 molecule results in a non-infectious non-pathogenic virus. Monoclonal antibody studies show that some antibodies bind with high affinity and block HIV fusion and infection more efficiently than other anti-CD4 antibodies. We have therefore used one of these antibodies, Leu 3a, to try and raise an anti-idiotype response which should resemble the CD4 molecule. More recently numerous soluble CD4 preparations have been made and these have been able to neutralise all HIV isolates and appear to have no detrimental effect in immunological assays either *in vitro* and *in vivo*.

Using an Allum precipitated immunisation schedule the monoclonal antibody Leu 3a has been able to raise polyclonal response which has weak antiviral activity using a fusion plaque assay system. The antiviral titres are in the region of 1–200 whereas the background is 1/50 to 1/100. However, by diluting the internal image response we have been able to show that this activity is not a specific anti-idiotypic response. Mapping of Leu 3a to CD4, as well as CD4 mutants, show that it is closest to the gp120 binding site and more closely resemble gp120 binding that other available monoclonal antibodies. A similar trial in human patients with ARC without using an adjuvant has led to the successful induction of specific anti-idiotypic responses in these patients. There has also been no shoter intermediate adverse effects from this schedule. Again, no specific antiviral activity has been detected in these patients over and above their natural immunity.

Recent construction of CD4 human immunoglobulin hybrids has shown that only the part of CD4 which is recognised by Leu 3a and similar monoclonal antibodies is required to effectively neutralised all HIV isolates. The above studies show that although close Leu 3a is not the ideal immunogen and we are now engaged in searching for an anti-CD4 immunogen which would raise the idiotype recognised by the CD4 human IgG hybrid, which could clearly be a vaccine strategy which would protect against all isolates.

### Radiation therapy for AIDS related Kaposi's sarcoma

A.M. Brunt, A.G. Goodman & R.H. Phillips

*Radiotherapy department, St Stephens and Westminster Hospitals, London, UK.*

Kaposi's sarcoma is diagnosed in almost 40% of patients with AIDS at our institution. One hundred and ninety-four cases were diagnosed by the end of 1988. These patients have a median survival of 20 months, and where the cause of death is known 38.6% died directly due to the Kaposi's sarcoma and in a further 11.4% it was considered to be an important contributing factor. We aim to palliate the disease where indicated. The indication for radiotherapy in our department has been for cosmesis in 63.5% and for pain and functional complaints in the remainder. The lower limb and the head and neck (excluding the oral cavity) comprise over two thirds of the treated sites. The majority of treatments are with superficial X-rays, though megavoltage X-rays are used when indicated. We report the largest British series to date: 74 patients received a total of 352 courses of radiation therapy up to 31 December 1988. Response is assessed on a five point scale; complete response with and without residual pigmentation, partial response, static disease and progression. The response is assessed at 1, 3 and 6 months with 51.5% achieving a complete and 15.9% a partial response at 3 months and 53.6% of lesions assessed at 6 months exhibiting a complete response. The majority of treatments have been given using either a single fraction of 800 cGy or 10 fractions of 180–200 cGy, these regimes are compared as showing no significant difference. Radiotherapy is well tolerated except for the severe mucositis often observed when treating the oropharynx.

### The results of treatment of 102 patients with locally recurrent soft tissue sarcoma

M.H. Robinson, C. Fisher, C.L. Harmer & G. Westbury

*Sarcoma Unit, Royal Marsden Hospital, UK.*

The results of treatment of 102 patients with locally recurrent non-metastatic soft tissue sarcoma (STS) are reported. Thirty-two tumours were situated in the lower limb, 24 the upper limb, 22 pelvic and shoulder girdle, eight trunk, seven abdomen, and nine head and neck. Histology comprised MFH 32 cases, liposarcoma 22, synovial sarcoma 12, others 36. Fifty-seven were high grade, 21 intermediate and 24 low. Grade increased in six and decreased in seven patients with recurrence. The median size of tumour at presentation and at first local recurrence was 5 cm. Surgical clearance of the first local recurrence was 'adequate' (Enneking) in 39% of assessable patients, including 8/12 amputations, but in only 18% of the original operations which included two amputations. Thirty-three patients were irradiated as part of the treatment of the primary disease, only two of whom had had an 'adequate' surgical clearance. Thirty-two patients were given radiotherapy following first local recurrence. For six patients this involved re-irradiation. Median follow-up was 36 months. Actuarial local control of those with 'adequate' surgical clearance of their first local recurrence was 80% at 2 years compared with 55% in those with 'inadequate' clearance ($P < 0.025$). Survival (85% *vs* 65%) was also significantly different ($P < 0.025$). Local control and survival were significantly better in those with upper or lower limb tumours compared to other sites. Local control at 2 years (70% *vs* 45%) was significantly better in those receiving radiotherapy as part of the treatment of their first recurrence ($P < 0.025$). Survival and local recurrence were significantly worsened by tumour size > 10 cm but not grade. The roles of surgery and radiotherapy in the management of recurrent STS will be discussed.

### The spectrum of histological response to preoperative chemotherapy in primary malignant bone tumours

J.A.S. Pringle & R.L. Souhami

*London Supra-regional Bone Tumour Service, London, UK.*

Recent advances in the treatment of primary malignant tumour of bone have included chemotherapy given before and after surgery and in many cases local resection and prosthetic replacement rather than amputation. The types of bone tumour treated in this way include high grade osteosarcoma (central and surface), periosteal osteosarcoma, Ewing's sarcoma, and malignant fibrous histiocytoma (MFH). The problems encountered by the pathologist vary in each tumour type. In osteosarcoma there is a wide spectrum of 'response' which falls short of necrosis and may give the appearance of 'increased differentiation'. In Ewing's sarcoma, response is often associated with much reactive bone formation and micro-deposits of viable tumour may be seen microscopically where the clinical and radiological response appears complete. Periosteal osteosarcoma responds by increasing mineralisation, but rarely by complete necrosis. While with MFH, reparative fibroblastic tissue may be difficult to distinguish from residual fibroblastic tumour. This study confirms that these four categories of primary bone tumour are chemosensitive and although response may fall short of the 90% necrosis accepted as likely to indicate good prognosis, a more modest response associated with increased mineralisation may enable the surgeon to perform limb-sparing surgery.

### Biology

### Enhancement of lung metastases by interferon-γ

S.A. Kelly & F.R. Balkwill

*Imperial Cancer Research Fund, London, UK.*

Incubation of a lung colonising variant of Colon26 (an undifferentiated murine colone adenocarcinoma cell line) *in vitro* with rMuIFN-γ, but not rIFN-αA/D, leads to a significant increase the number of lung colonies in syngeneic BALB/c mice in an experimental metastasis assay. (Median number of colonies: control, 22; rIFN-αA/D 18; rMuIFN-γ200.) A 1 hour incubation is sufficient to see this enhancement.

Following i.v. injection all cells are found in the lungs, but control and rIFN-αA/D pretreated cells are cleared at a significantly faster rate than those pretreated with rMuIFN-γ. The differences are mainly seen in the first 24 hours following injection. This implicates NK killing, and we have some evidence to support this in that in models where animals are depleted of NK cells (beige nude mice, and anti-asialo GM-1 treated mice) the enhancement of metastases by IFN-γ is not seen. However, Colon26 do not form conjugates with NK cells and are completely resistant to NK lysis *in vitro*.

Some investigators have correlated an enhancement of class 1 MHC by interferons with increased resistance to NK lysis, and suggested that this is the mechanism of enhancement of metastatic potential. This is unlikely to be the case in our model, since both interferons equally enhance class 1 MHC.

### Paradoxical effects of TNF in intraperitoneal (i.p.) human ovarian cancer xenograft models: prolongation of survival and enhancement of metastasis

S.T.A. Malik, D.B. Griffin & F.B. Balkwill

*Imperial Cancer Research Fund, PO Box 123, Lincoln's Inn Fields, London WC2A 3PX, UK.*

The effects of i.p. therapy with low dose (1 μg daily) recombinant human TNF was investigated in three intraperitoneal human ovarian cancer xenograft models (OS, LA and HU). Parameters studied included: (a) effect on survival; (b) sequential cytological immunophenotypic analysis of blood and peritoneal cell populations (PEC); (c) sequential analysis of macroscopic and microscopic tumour in the peritoneal cavity and peritoneal biopsies; and (d) variations of dose-schedule regimes. TNF prolonged survival in the HU and LA xenografts (median survival: control HU = 22 days; TNF treated HU = 52 days ($P < 0.001$); control LA = 22 days, TNF treated LA = 52 days ($P < 0.001$)). No survival benefit was seen in the OS xenograft. The major change in the peripheral blood and PEC was a neutrophil leucocytosis, which was greater in tumour mice ($P < 0.01$). Daily therapy led to dimunition of the leucocytosis, and led to a fall in haemoglobin and platelet counts ($P < 0.005$). A significant rise in peritoneal Thy 1.2 positive cells was noted at 3 weeks. Scheduling experiments show that daily therapy was not necessary and equivalent survival benefit was gained with three times a week treatment or once weekly high dose (7 μg) treatment. Contrary to these positive effects on survival, post-mortems revealed that while TNF caused necrosis of free floating peritoneal tumours, it increased the mesothelial hyperplasia induced by xenografts, and led to invasion of the peritoneum by the tumours. This was also seen in the OS xenograft which was resistant to TNF therapy. These results may suggest a role for endogenous TNF production in peritoneal metastasis.

### Proliferation in human tumours measured by Ki67 antibody labelling: its potential clinical use

P. Price, C. Bush, J. Norton, C. Jones, M. Bailey, C. Parkins, M. Robinson, P. Blake & A.H. Horwich

*Radiotherapy Research Unit, Institute of Cancer Research and Royal Marsden Hospital, London and Sutton, UK.*

The monoclonal antibody Ki67 recognises a human nuclear antigen expressed in proliferating cells. This antibody has been used to assay the proliferative fraction (PF) of primary bladder and cervical tumours. This technique carries the advantage of not requiring prior injection of thymidine anologues, only requiring small amount of fresh tissue and material can be collected at routine biopsy. Biopsies have been obtained from 27 patients with bladder cancer and 22 with cervical carcinoma. Tumour specimens were snap frozen and then cryostat sections were stained with Ki67 using an indirect immunoperoxidase method. Ki67 positive nuclei were counted under light microscopy and a Ki67 PF (the number of Ki67 positive tumour cells divided by total number of tumour cells %) was derived by counting $>500$ tumour cells per sample. Intrasample variation was $<5\%$. A wide range of Ki67 PF were detected, ranging from 7.4 to 65.8% for bladder carcinoma and from 2.4 to 46.8% for the cervical carcinomas. Ki67 PF correlated with known prognostic factors: TNM stage and grade for bladder carcinoma and Figo stage and histological grade for cervical carcinoma. Three bladder patients had metastases at presentation and two of these had the highest Ki67 values. Further follow-up is required before the Ki67 PF can be evaluated as an independent prognostic variable and as a predictor for response to radiotherapy.

### Analysis of gene regulation in teratocarcinoma stem cells with the episomal plasmid, L factor

M.J.C. Ellis & S.E.Y. Goodbourn

*Gene Expression Laboratory, Imperial Cancer Research Fund, Lincoln's Inn Field, London WC2, UK.*

Germ cell tumours are striking examples of how normal differentiation can be disrupted by malignant transformation. Instead of producing gametes, these tumours give rise to tissues exhibiting features of embryonal development. Cell lines derived from both murine and human germ cell tumours can be induced to differentiate *in vitro* by exposure to retinoic acid. In order to investigate the molecular basis of how genes are regulated during teratocarcinoma stem cell differentiation we have stably introduced test genes into undifferentiated mouse F9 teratocarcinoma cells using the episomal vestor L factor, a plasmid related to polyoma virus that replicates extrachromosomally as closed circles of DNA (Nashimori *et al*., *Mol. Cell Biol*., **8**, 2097). The activity of the test gene was followed during retinoic acid induced differentiation using a sensitive RNA mapping technique. We have demonstrated that the inducibility by double-stranded RNA of an L factor linked β-interferon gene mirrors the increasing inducibility of the endogenous mouse beta interferon gene during F9 differentiation. Viral enhancer elements linked to L factor also become activated in a manner which parallels the behaviour of these elements in intact viral genomes. By mutating key regulatory sequences and introducing them into this system we have a powerful method for investigating how differentiation specific gene expression takes place and how it may be disrupted by malignant transformation.

### Bone resorbing tumours inhibit the growth and function of osteoblast-like cells *in vitro*

C.E. Evans, C.S.B. Galasko & C. Ward

*University of Manchester, UK.*

The destruction of bone is normally accompanied by reactive new bone formation. This occurs with nearly all metastases, but not with most myelomata, which produce lytic lesions which do not concentrate bone-seeking isotopes. We have developed a bioassay using cells grown from human cancellous bone (BDC) to examine the effect of myeloma on osteoblast-like cells. Previous studies have shown that these cells exhibit a range of osteoblast-like characteristics. Two myeloma cell lines (GM1500 and Karpas) were cultured *in vitro*, plus myeloma tissue from a patient with myeloma (McA). Culture medium in which these cells had been grown (MCM) was used in the bioassay; BDC were cultured with either MCM or a control medium. Two parameters of cell growth, cell number and DNA synthesis, were assayed after the BDC were incubated with the MCM for 48 hours. MCM from GM1500 caused a mean decrease of 29% in cell number and of 47% in DNA synthesis. Control experiments demonstrated that this was not due to a general, cytotoxic effect. Incubation of BDC with medium conditioned by other BDC, or by skin fibroblasts (FB) did not show such inhibition. Similarly, no inhibition was seen of FB incubated with MCM. Also, the inhibition of BDC by MCM was density and dose-dependent. MCM from McA and from Karpas cells inhibited cell numbers by 18% and 31% respectively; DNA synthesis was inhibited by 71% and 17% respectively. Similar inhibitory effects, seen with MCM from another bone-resorbing tumour, breast carcinoma, are still under investigation. These results confirm that myeloma secretes an osteoblast-inhibiting factor(s) and explain why myeloma is different to other bone-resorbing tumours, and why scintigrams are frequently negative.

### Reconstruction and restoration of function in cancer surgery

### Reconstruction in the head and neck

N.M. Breach

*Royal Marsden Hospital, London, UK.*

Reconstruction of the head and neck has to encompass the functions of the part: respiration, mastication, swallowing, speech and cosmesis. Following the excision of tumours, restoration of function requires the replacement of that part which has been removed. The concept of reconstruction has changed in recent years as knowledge of the blood supply to the skin has reduced it from a multistaged procedure to a single operation, through the use of myocutaneous and free flap transfer.

Problems of colour match, skin quality and function bedevil the reconstructive surgeon. The introduction of distant flaps to the face results in a patch-like deformity. The thickness of the subcutaneous tissues may vary giving problems of bulk. External skin is frequently used to replace the mucosa; in this situation the flap will be non-sensory, non-secretory, possibly hairy and immobile, whereas the mucosa is sensate, does secrete, does not grow hairs and is mobile.

Oral and pharyngeal reconstruction must be considered as a three-dimensional concept. Frequently a single flap is used in the repair, which may result in the limitation of function. Examples will be shown to illustrate the modern concept of reconstruction with particular reference to the single staged compound flap repairs.

### Reconstructive surgery for primary malignant tumours of bone

H.B. Kemp

*Royal National Orthopaedic Hospital, London, UK.*

Before the introduction of specific cytotoxic therapy by Rosen *et al.* (1979), the only available surgical treatment of malignant bone tumours was amputation. Even the more refined combination of radiotherapy and interval amputation advocated by Stanford Cade (1955) only marginally improved the 5-year survival rate above 10%.

Cytotoxic therapy facilitated a more conservative approach to these forms of malignant disease. In France, Merle d'Aubigne (1958) advocated resection and reconstructive auto grafting; in the USA, Henry Mankin and others (1956) used cadaver bone as homografts, while in Germany, Winkelmann (1983) resected the tumour approximating the residual bone and with lesions of the lower limb rotated the distal part through 180 degrees so that the ankle could be employed as a knee joint (rotation turniplasty). All these methods were excellent in concept; the disadvantages were the length of time that elapsed before the limb could function. Function was necessarily of limited achievement.

In this country, Burrows *et al.* (1975) had already had experience in the treatment of chondrosarcoma and extensive benign tumours, replacing the affected bone and associated joint with an internal prosthesis. With the advent of bone cement more sophisticated fixation of the prosthesis was achieved. In consequence, over 1,000 bone tumours have now, in the short-term, been successfully treated in the two supra-regional centres.

Today, due to the use of cytotoxic therapy in conjunction with surgery, the survival rate of patients with Ewing's sarcomas and osteogenic sarcomas is in the region of 60%.

This particular method for treatment has certain disadvantages. Prosthetic loosening and prosthetic fracture are now essentially minor problems, but the risk of infection in patients who are immunologically compromised as a sequel to cytotoxic therapy must obviously remain high and, although the majority of infections respond adequately to antibiotic therapy, 2% of patients will develop extensive osteomyelitis necessitating amputation. The advantage of this form of management is that the patient can be rapidly rehabilitated to near normal function and the body image is maintained, a feature of some consequence to the adolescent and young adult.

### Reconstructive urology

C.R.J. Woodhouse

*Royal Marsden Hospital, St George's Hospital and The Institute of Urology, London, UK.*

Reconstructive urology in this context means provision of a neo-bladder which is continent and which is usually emptied by intermittent clean self-catheterisation (ICSC). Experience in children and adolescents has shown that no patient need have an external urinary diversion with a bag. The question now arises when and how the same should be applied to the cancer patient. Reconstruction should not be confined to a named operation such as the 'Kock', the 'Indiana' or the

'Mainz'. Three mutually independent components are necessary: a container for the urine, a means to conduct the urine to the surface and a continence mechanism. One or more items for each of the components may be selected from the lists in the table and used for reconstruction tailored to the patients needs.

| | |
|---|---|
| Container: | Bladder, stomach, ileum, caecum, colon |
| Conduit: | Urethra, appendix, fallopian tube, ureter, skin tube, ileum, stomach tube |
| Continence mechanism: | Urethral sphincters, detrusor tube, artificial sphincter, Kock nipple, Mitrofanoff principle, ileocaecal valve, anal sphincter |

As much as possible of the natural urinary tract should be included. Otherwise my first choices are large bowel for the pouch, appendix for the conduit and the Mitrofanoff principle (tunnelling a narrow tube such as the appendix into the wall of the pouch) for continence. The surgeon's selection of patient is the relatively fit, thin, well motivated and potentially curable – about 50% of patients. The patient may be unwilling to risk the additional surgery required for reconstruction or may not like the idea of ICSC. This eliminates a further 25%. Ten of 39 continent diversions in my series have been in cancer patients. Two died in the immediate post-operative period although in only one was the reconstruction a contributory cause. Three patients have had delayed recovery. One patient has had a revision for difficulty with catheterisation. Major series throughout the world report a complication rate of about 25% with an acceptance rate by the patients between 15 and 98%. There has been general acceptance by the patients and hospital staff of the superiority of reconstruction over an external bag but selection must be meticulous.

### Reconstruction and the surgical oncologist

J. Meirion Thomas

*Westminster and Royal Marsden Hospitals, London, UK.*

Immediate reconstruction following either curative or palliative cancer surgery offers enormous advantages. The knowledge that a safe, simple and reliable method of reconstruction is available allows the surgeon to resect tumours with a wide margin of clearance. This is particularly important in the treatment of post-irradiation recurrent tumours where the anatomical limit of the tumour may be difficult to define and where surrounding disease-free but irradiated tissues heal notoriously badly. The transfer of healthy, unirradiated and well-vascularised tissues into an irradiated wound allows wound closure without tension, accelerates and greatly improves the chances of primary healing, protects against infection and fills spaces where damaging haematomas might have collected. New reconstructive techniques which have come into common use over the past decade have enhanced the scope of surgical oncology. Multi-staged reconstructive techniques are rarely necessary and patients can be offered immediate reliable reconstruction thereby reducing morbidity and time in hospital. The purpose of this symposium is to describe the reconstructive techniques available and illustrate their place in patient management. Skin and soft tissue is the main requirement following ablative surgery and the various myocutaneous transposition techniques will be described but occasionally blood vessels, nerves, bone and joints need reconstruction. Reconstruction is most often required for post-irradiation recurrent tumours and this presentation will describe reconstructive processes which have been necessary in the surgical treatment of sarcomas, melanomas, breast cancers and cloacal tumours extensively involving perineal skin.

### Central nervous system malignancy

### Pineal and CNS germ cell tumours. Royal Marsden Hospital 1962–1987

S.J. Whitaker, R.P. A'Hern & D.P. Dearnaley

*Royal Marsden Hospital, London and Sutton, UK.*

Pineal and intracranial germ cell tumours are rare, comprising less than 1% of all primary intracranial malignancies. We present a retrospective analysis of cases presenting to the Royal Marsden Hospital between 1962 and 1987, documenting prognostic factors, patterns of relapse and survival together with an assessment of quality of life and initial results with platinum containing chemotherapy.

Sixty-eight cases were seen after initial presentation and were divided into three groups: (1) germinoma (36 cases), (2) malignant teratoma (11 cases), and (3) non-germ cell tumours (21 cases). Germ cell tumours were treated with whole CNS irradiation (tumour dose 51 Gy, whole brain and spine dose 30 Gy over 8 weeks), and radioresistant tumours with local RT fields (50 Gy over 7 weeks). Eight patients received cisplatin containing chemotherapy.

Cause specific 5 and 10 year survival was 84% in germinoma, 20% for malignant teratoma and 64% and 47% respectively for non-germ cell tumours. Neurological status of survivors was good with 86% MRC grade 1 or 2, and 66% had normal schooling and 76% obtained normal employment. Thirty eight percent required hormone replacement. Cisplatin containing chemotherapy produced two CR and six PR.

Whole CNS RT produces excellent survival and good quality of life for germinomas but the treatment of CNS teratomas is unsatisfactory. The role of platinum containing chemotherapy and aggressive surgery remains to be defined in this group.

### The late effects of cerebral irradiation

R.P. Beaney, D.J. Brooks & T. Jones

*QEH, Birmingham and Hammersmith Hospital, London, UK.*

The purpose of this study was to examine the late effects of cerebral irradiation. The aim was to establish whether particular parts of the brain showed a predilection for radiation damage; and if damage was demonstrable ascertain if chronic ischaemia played a role in its pathogenesis.

Five patients with gliomas (two grade III astrocytoma, two grade II astrocytoma and one oligodendroglioma) were studied at least 12 months after irradition (mean 18 months). The patients tumour volume received 60 Gy in 30 fractions over 42 days. The technique of positron emission tomography was used to study regional cerebral blood flow (rCBF), regional metabolic rate for oxygen (rCMRO$_2$) and fraction of oxygen extracted from the arterial blood (rOEF). There were marked regional variations.

Our results show that abnormal brain expresses late radiation damage more readily than normal brain and white matter more readily than grey matter. Ischaemia (inappropriately low blood flow for local metabolic demands) cannot be the main reason for the late radiation effects because the rOEF lay within the normal range (0.3–0.5).

| | CBF ($ml\ 100\ ml^{-1}\ min^{-1}$) | OEF | $CMRO_2$ ($ml\ of\ O_2\ 100\ ml^{-1}\ min^{-1}$) |
|---|---|---|---|
| Tumour | 9.5 ± 2.6 | 0.42 ± 0.03 | 0.64 ± 0.23 |
| Perifocal oedema | 11.8 ± 1.9 | 0.38 ± 0.05 | 0.82 ± 0.19 |
| Irradiated white | 13.9 ± 2.6 | 0.43 ± 0.03 | 1.04 ± 0.18 |
| Remote white | 20.2 ± 3.5 | 0.41 ± 0.05 | 1.42 ± 0.52 |
| Irradiated grey | 29.6 ± 4.6 | 0.43 ± 0.03 | 2.24 ± 0.32 |
| Remote grey | 31.7 ± 4.0 | 0.42 ± 0.02 | 2.31 ± 0.21 |

### Brain tumour imaging with positron emission tomography (PET) using F18 5-fluoro-2-deoxyuridine (FUdR)

S. Gill, C. Wilson, J. Heather, F. Brady & T. Jones

*MRC Cyclotron Unit, Hammersmith Hospital, London W12 0HS, UK.*

The use of 18F-fluoro-2-deoxyuridine (FUdR) and PET to outline tumour boundary and to estimate tumour proliferation is being evaluated. Fourteen patients (nine high grade (3–4 glioma, three low grade (2) glioma, one cerebral lymphoma, one cerebral metastases) have been investigated. seven patients were imaged with CT and PET in a relocatable stereotactic frame allowing precise three-dimensional measurement. A biopsy trajectory was planned using CT and PET generated co-ordinates and serial biopsies taken from the edge through the tumour. Histological examination to define the tumour extent and ki67 antibody labelling to evaluate proliferative capacity were performed. Both scans were reconstructed along the place of biopsy trajectory allowing pixel by pixel comparison with the biopsy material. In this series of patients a close correlation was found between tumour boundaries defined by PET and histology. This was not the case with CT images. There is also accurate matching of the labelling index of the tumours with tracer uptake in that highly proliferative area show the greatest signal. No signal is seen in low grade gliomas, and no specific uptake was obtained in the patient with cerebral metastases.

### Spinal cord ependymoma

S.J. Whitaker, E.M. Bessell, H.J.G. Bloom & M. Brada

*Academic Radiotherapy Unit, The Royal Marsden Hospital, Fulham Road and Sutton, UK.*

The aim was to define the natural history of spinal cord ependymoma and the criteria for treatment.

Retrospective review of 52 patients with histologically verified spinal cord ependymoma treated at the Royal Marsden Hospital and Atkinson Morley's Hospital between 1951 and 1987. Patients were aged 1.5–80 years, (median 40); six had cervical, seven thoracic, 15 conus medullaris, 20 cauda equina and four more than one region of cord involvement. Forty three were low, five high grade and four ungraded. Progression was defined as worsening of clinical signs with or without radiological confirmation. Median follow-up was 70 months (3–408).

Twelve patients had complete, 30 partial excision and 10 biopsy only; 41 patients received postoperative radiotherapy (RT): 28 received RT to the primary site alone (50 Gy), 13 had whole neuraxis RT (30–35 Gy) followed by boost (15–20 Gy). Four patients also received adjuvant chemotherapy.

Thirteen of 40 patients with residual disease progressed. The 5 and 10-year progression-free survival (PFS) were 73%. Following complete excision the progression rate was low. PFS was worse with high compared to low grade tumours. The 10-year cause specific survival was 73%.

The main problem in the management of spinal cord ependymoma is failure of local control. It is related to tumour grade and the extent of resection. RT may improve local control in incompletely excised tumours.

### Single dose prophylactic cranial irradiation (PCI) for small cell lung cancer (SCLC)

P.A. Burt, N. Thatcher & R. Stout

*Manchester Lung Tumour Group Christie Hospital, Manchester M20 9BX, UK.*

Two hundred and fourty eight patients with limited-stage (LS) SCLC have been treated since 1981 using short intensive chemotherapy and thoracic irradiation in a 3–6 month regimen. PCI was not given.

The first 129 patients had a complete remission rate (CR) of 55% with a 2-year survival of 10%. Relapse in the brain occurred in 20% and it was the only site in 9%.

Between 1984 and 1986, the CR rose to 70% and the 2-year survival to 25% with improved chemotherapy. A parallel rise in cranial relapse was noted, 36% overall and 15% as the sole site.

Conventional schedules of PCI have not demonstrated any improvement in long-term survival. In 1986 we included PCI in an unconventional form, giving a single fraction at 8 Gy (800 cGy) using a parallel opposed pair 48 h after the first course of chemotherapy. Of 33 patients (LS) treated the 2-year survival is 33%. The cranial relapse rate is 12% overall and 9% as sole site. This represents a significant reduction in cranial relapse which may have contributed to the overall improvement in survival.

### Genito-urinary and gynaecological malignancy

### Low toxicity platinum combination chemotherapy (PMO) for urothelial cancer

J. Gildersleve, D.P. Dearnaley, A. Horwich, J.W.A. Ramsey, W.F. Hendry, R.W. Shearer & C.R.J. Woodhouse

*Royal Marsden Hospital, Sutton and St Bartholomew's Hospital, London, UK.*

Metastatic transitional cell cancer (TCC) of the bladder is sensitive to chemotherapy but high response rates (Harker *et al.* (1985) *J. Clin. Oncol.*, **3**, 1463; Sternberg *et al.* (1988) *J. Urol.*, **139**, 461) have been associated with marked toxicity. We have developed a less intensive schedule and report preliminary results.

Forty-six patients with metastatic or locally advanced carcinoma of bladder (43), TCC of kidney (2) or ureter (1) were treated. Median age was 69 years. Thirty-four patients had recurrent disease, the other 12 presenting with locally advanced or metastatic disease. Chemotherapy (PMO) was given with cisplatin 60 mg $m^{-2}$, methotrexate 60 mg $m^{-2}$ and vincristine 2 mg on a 21 day schedule. Carboplatin was substituted for cisplatin if EDTA $\leqslant 55\,\mathrm{ml\,min^{-1}}$.

Subjective toxicity was mild. Of chemotherapy courses 4.7% were associated with WBC <2, with only two episodes of neutropenic sepsis. EDTA fell $13\,\mathrm{ml\,min^{-1}}$ per course of cisplatinum. CR occurred in 13% and PR in an additional 22%. Response by site of disease was lymph nodes 63%, lung 36%, bladder 33%, liver 25% and bone 19%.

PMO chemotherapy can be safely delivered to a relatively elderly and frail population of patients with advanced TCC and is presently under assessment by the Co-operative Urological Cancer Group (CUCG).

**The significance of dose, nodal irradiation and other factors upon outcome after radiotherapy for bladder cancer**

R.P. Symonds, S.E. Davidson, T. Habeshaw, A.G. Robertson, M.P. Snee & E.R. Watson

*Beatson Oncology Centre, Western Infirmary, Glasgow, UK.*

To try to improve local control and survival we have included pelvic lymph nodes within the irradiated volume for part of the treatment and gradually increased the dose to the bladder. Between 1978 and 1984, 731 patients were treated with curative intent. Three hundred and thirty-six had radiotherapy (4 MeV) to only the bladder and 395 irradiation of the pelvic lymph nodes (40–42.5 Gy in 20 fractions). We have compared retrospectively local control and survival of similar sequential groups of patients receiving 60 Gy, 62.5 Gy, 64 Gy and 72 Gy (split course) in 30 fractions. The overall actuarial 5-year survival was 30.9%, by stage as follows: T1 (86.9%), T2 (49.1%), T3 (27.4%) and T4 (2.2%). Nodal radiotherapy had no effect upon survival, stage for stage survival was identical but serious complications were increased (9.6% nodal radiotherapy *vs* 4.3% bladder only).

There is a tendency which is not statistically significant for increased local control with higher doses but survival is very similar in all groups. If a gap of 2–3 weeks is included in the treatment schedule survival is reduced from 35.3% to 21.5% ($P = 0.001$). The most important prognostic factors are not differences in radiotherapy technique but biological parameters such as tumour stage, histology, degree of differentiation and patient age.

**Stage I and II carcinoma of the cervix treated by preoperative irradiation and Wertheim's hysterectomy**

H.F. Hope-Stone

*Department of Radiotherapy and Oncology, The London Hospital, London, UK.*

Early carcinoma of the cervix can be treated by surgery or radiation alone or by a combination of both. This paper describes a large series of patients treated by preoperative intracavity irradiation followed by immediate Wertheim's hysterectomy. After initial staging, a Curietron after-loading technique is used: a plastic intra-uterine tube is placed in the cervix and is attached to a vaginal applicator. The whole apparatus is held in place by a harness attached to a body corset. X-ray pictures are taken (using dummy wires) on a treatment simulator in the radiotherapy department. The patient is then connected to the Curietron and stays in a single fully-protected room for the duration of treatment. The caesium dose rate is relatively high, and allows a dose of 2,900 cGy at point A to be given in 40 h. A second insertion is carried out one week later, giving the same dose. Twenty four hours after the second insertion is completed a Wertheim's hysterectomy is carried out, with a meticulous lymph node dissection aided by preoperative lymphangiography and radiological operative survey to remove any remaining lymph nodes. If the pathology report shows lymph node involvement by tumour, then post-operative external irradiation is given to the pelvis, with a pair of opposed fields to give a tumour dose of 6,000 cGy (from a combination of the preoperative Caesium and the external irradiation) in 20 treatments. The rectum is protected by a mid-line parametrial wedge lead shield. The 5-year survival figures for stage I and II tumours are 94% in 143 cases; 23% showed lymph node involvement. There was no major post-irradiation or surgical mortality; the fistula rate was nil.

**Prostate specific antigen (PSA) and bone scintigraphy in the diagnosis of prostatic malignancy**

R.J. Iorns, M.A. Macleod & I.L. Jenkins

*Department of Nuclear Medicine, Royal Naval Hospital, Haslar, Gosport, UK.*

Prostate specific antigen serum assay has been successfully used as an indicator of prostatic malignancy for several years. In addition it has been claimed that PSA values are raised in cases of benign prostatic hyperplasia, thereby increasing the level of false positive values for malignancy. In this study we have compared PSA assay levels with bone scintigraphy in known cases of prostatic adenocarcinoma and benign prostatic hyperplasia.

Data on 67 cases of histologically defined adenocarcinoma (ADC) and 73 cases of benign prostatic hyperplasia (BPH) are presented. In patients with ADC the PSA level was normal in six (9%), equivocal in eight (12%) and raised in 53 (79%). Of the 45 bone scans performed in this group 29 were positive for metastases and 16 negative. In the group with 29 positive scans the PSA was raised in 23 cases, equivocal in three and normal in three cases. In the 73 cases of BPH, PSA levels were normal in 30 (41%), equivocal in 25 (34%) and raised in 18 (25%) cases. Only six bone scans were performed in this group of 73 patients with BPH, five of which were positive for metastases and one negative. Of the five positive bone scans PSA was raised in four and equivocal in one case, while in the negative bone scan the PSA was normal. We conclude that PSA is a sensitive indicator for prostatic malignancy and we intend to investigate further, patients with a diagnosis of BPH as the results suggest the possibility that these cases may in fact have underlying malignancy and a raised PSA serum value cannot be dismissed as a false positive.

**An evaluation of peritoneal lavage cytology in the detection of residual epithelial ovarian cancer**

C.W.E. Redman, E. Nicholl, S.Y. Chan, E.J. Buxton, G. Blackledge & D.M. Luesley

*Dudley Road Hospital, Birmingham, UK.*

As epithelial ovarian cancer (EOC) is largely confined to the peritoneal cavity, peritoneal lavage fluid (PLF) cytology may detect otherwise occult small volume disease. The use of indwelling peritoneal catheters and intermittent percutaneous cannulæ primarily for IP treatment has enabled diagnostic peritoneal lavage to be performed repeatedly on an out-patient basis. Of 207 PLF samples obtained from 86 EOC patients, 190 (92%) were suitable for cytological analysis whereas 17 were acellular. Sixty-seven EOC patients with residual disease prior to treatment had PLF samples suitable for cytological analysis and serum CA125 estimations and 51 of these patients had end of treatment samples. Thirty patients (45%) had small volume (<2 cm) residual disease at the end of the primary laparotomy. Fifty-three patients had advanced disease (FIGO stage III or stage IV). Peritoneal cytology was positive in 38 samples (57%) and demonstrated disease in four patients who were clinically and radiologically free of disease. Clinical and cytological findings alone had a sensitivity of 72% (48/67) while serum CA125 levels were raised in 58 (87%) patients. The presence of malignant cells in pre-treatment peritoneal lavage fluid was a major adverse prognostic factor. Thirty-one patients had positive cytology (53%) of which only four patients (32%) were alive at 12 months compared to 20 out of the 28 (71%) cyto-negative patients. Twenty-two patients had malignant cells in PLF following treatment and 20 (91%) of these patients have relapsed and died. Eleven patients had positive cytological findings in the absence of positive clinical or radiological findings. Of the nine patients who have relapsed (median time to relapse = 4.5 months, range = 1.6–16.9 months), all had elevated serum CA125 levels. Peritoneal cytology can detect clinically and radiologically occult disease but confers no advantage over serum CA125.

### Lymphomas

### Long-term follow up of BNLI study of CHOP chemotherapy in patients with advanced high grade non-Hodgkin's lymphoma

G. Vaughan Hudson, D.C. Linch, B. Vaughan Hudson, M.H. Bennett, K.A. MacLennan & L. Anderson

*British National Lymphoma Investigation, University College and Middlesex School of Medicine, London, UK.*

Between February 1974 and December 1984 296 patients with stage III/IV large cell or mixed small and large cell non-Hodgkin's lymphoma (NHL) were treated initially with CHOP combination chemotherapy. Seventy-five per cent of patients were aged 50 years or above. Sixty-three per cent had stage IV disease, 47% had B symptoms, 43% had an ESR $>40\,mm\,h^{-1}$, 27% had mediastinal involvement, 26% bone marrow involvement and 36% presented with a low albumin. The complete remission rate was 49%, the DFS 27% and the overall survival 36% at 5 years. In these patients who attained a CR the survival was 67% at 5 years with little evidence as yet of a plateau. The complete remission rate was dependent on stage being 73%, 41%, 51% and 33% for stages IIIA, IIIB, IVA and IVB respectively. In a group of 86 patients with stage I/II high grade NHL treated with CHOP over this period the CR rate was 61% and overall survival at 5 years 41%. The serum albumin at presentation was an equally powerful prognostic factor for the attainment of complete remission as stage IVB patients with a low albumin having a CR rate of 29%. Age had no effect on CR rate. Once CR was attained these prognostic factors became irrelevant. The results of salvage therapy at all stages were poor. This study indicates that CHOP therapy will mainly cure one-third of patients with advanced high grade NHL and in those with IVB disease and a low albumin the prospects for cure are very low.

### Radiotherapy after intensive chemotherapy and autologous bone marrow transplantation for resistant Hodgkin's disease

A.K. McMillan, J.G. Gribben, J.S. Tobias, D.C. Linch & A.H. Goldstone

*Bloomsbury Transplant Group, University College and Middlesex Hospital School of Medicine, London, UK.*

Seventy-four patients with resistant Hodgkin's disease were treated with 'BEAM' chemotherapy and autologous bone marrow transplantation. The overall actuarial disease-free survival at 3 years is 62.5%. There were seven procedure related deaths (9.5%). Of the 65 patients evaluable for disease at 3 months there were 19 complete responders. These patients received no further treatment. There have been four relapses and one late toxic death from pneumonitis in this group. There was no response in six patients and two of these died soon after post graft reassessment. Thirty-three patients obtained a partial response to BEAM. Seventeen were given subsequent radiotherapy; a full dose mantle was planned in eleven patients, an inverted Y in four and 'top up' treatments in a further two patients. Of the 15 full courses there were two major delays and two reductions in dose because of myelosuppression. Two patients who received a mantle developed moderate pneumonitis requiring systemic steroid therapy. One patient (UPN 337) has received a sequential full mantle and para-aortic strip with no significant myelosuppression. Assessment of the response and progress of the 17 radiotherapy recipients is that four obtained a CR, although one later relapsed, eight have remained in stable PR and five have had disease progression. Twelve out of 17 seem to have responded favourably though this must be viewed with caution as two patients initially assessed as PR have obtained a later CR and a further four patients have had progressive improvement on CT scan over periods of one to three years without subsequent radiotherapy. This analysis indicates that radical radiotherapy post BEAM for Hodgkin's disease is possible in many patients but that demonstration of its efficacy will be difficult. It must be emphasised that residual masses on imaging for assessment do not invariably indicate that rapid disease progression will follow.

### The management of primary cerebral lymphoma with initial chemotherapy

M. Brada, D.P. Dearnaley, A. Horwich & H.J.G. Bloom

*Academic Radiotherapy Unit, Royal Marsden Hospital, Sutton and London, UK.*

Between 1986 and 1988 10 patients with primary cerebral lymphoma (PCL) were treated with initial MACOP-B chemotherapy followed by radiotherapy. All demonstrated radiological response to chemotherapy but this did not predict final clinical outcome. The overall median survival was 13 months. Patients with poor MRC neurological performance status (NPS) 2–4 had a median survival of 5 months. Only two of seven patients with NPS 0–1 died and the median survival is 18 months with a median follow-up of seven months (2–32 months). Quantitative sequential measurement of blood–tumour (BTB) and blood–brain barrier (BBB) with gallium EDTA and PET scanning in two patients suggests an early repair of BTB after chemotherapy.

The results were compared to 25 patients with PCL treated between 1963 and 1986 with radiotherapy as the main treatment modality. The overall median survival was 18 months. Patients presenting with poor NPS (2 and 3) had worse survival (median survival 8 months) compared to patients with good NPS (median survival 22 months; $P<0.025$). Patients diagnosed and treated from 1982 to 1986 had significantly worse prognosis when compared to earlier treated patients. None of the patients were in an 'at risk' group or had AIDS.

The results of combined modality therapy are not significantly different when compared to historical series and we have to await long-term outcome before recommending combined modality therapy as the treatment of choice. Studies of BBB and BTB should serve as a basis for future treatment strategies.

**Functional outcome following treatment of non-Hodgkin's lymphoma (NHL) presenting with spinal cord compression (SCC)**

R. Eeles, P. O'Brien & M. Brada

*Radiotherapy Unit, Royal Marsden Hospital, Sutton, Surrey, UK.*

Between 1971 and 1988, 20 patients with previously undiagnosed NHL presented with extradural SCC. All had intermediate and high grade NHL (Working Formulation). They were aged 12–75 (median 55) years. Thirteen had stage I and II and seven stage III and IV disease. The median follow-up was 42 months (15–163 months). Three patients were ambulant, nine paretic and eight paraplegic; 14 were incontinent of urine and 3 were faecally incontinent.

All patients had decompressive surgery. Mobility improved in 10 and 11 were fully ambulant. One patient deteriorated. Fifteen had normal sphincter function.

The first treatment after surgery was chemotherapy in nine and radiotherapy in 11 patients. Function remained unchanged in all regardless of the initial therapy. Median survival of 20 patients was 6 months (1–163 months). The strongest predictor of survival was mobility after surgery, even when corrected for age and stage at presentation.

Following surgical decompression, the functional outcome of patients with SCC due to NHL is not influenced by the choice of subsequent therapy. The treatment should be appropriate for prognostic factors such as histology, stage and age. Paraplegia after decompression carries a poor prognosis.

**The potential impact of estimated alpha-beta ratios on mantle technique**

D.J. Sebag-Montefiore, E.J. Maher & J. Young

*Regional Centre for Radiotherapy and Oncology, Mount Vernon Hospital, Northwood, Middlesex, UK.*

There is increasing interest in the late complications of radiotherapy together with awareness of the importance of dose per fraction, as well as dose. Dosimetry of the mantle field is complicated by the irregular treatment volume with varying distances between tumour and normal tissues across it. Published studies rarely provide sufficient information to allow estimation of dose and dose per fraction to normal tissues at risk. This study investigated the effect of variation in technique between radiotherapy departments.

Eleven superintendant radiographers were questioned about the 'mantle' technique in their departments. All aimed to deliver a minimum tumour dose of 30–35 Gy to microscopic and 35–40 Gy to macroscopic disease. None routinely recorded dose or dose per fraction to all normal tissues at risk (lung, spinal cord and heart).

There were variations in machine energy, patient position, points of prescription, shielding and field weighting between the departments. The techniques were compared in an 'ideal' patient and potential risks for normal tissue damage estimated using an alpha-beta ratio of 3 for heart and 1.5 and 3 for spinal cord. Considering the techniques used, there were variations of 5–30% in estimated biological damage to thoracic spinal cord and heart.

If useful studies are to investigate different radiotherapeutic techniques in the treatment of upper half Hodgkin's disease, dose and dose per fraction to lung, spinal cord and heart should be formally recorded in addition to minimum tumour dose.

### Poster presentations

### Laboratory research

**Intraperitoneal activation of lymphocytes by IL-2: initial haematological and immunological observations**

P.D. Allen & M.G. Macey, D.H. Johnson, N.S. Williams & A.C. Newland

*Departments of Haematology and Surgery, The London Hospital, Whitechapel, London El 1BB, UK.*

We have seen generation of lymphokine activated killer (LAK) activity after intraperitoneal infusion of blood lyphocytes, obtained by leukopheresis, incubated *in vivo* with simultaneously administered doses of recombinant IL-2 (rIL-2) increasing from 11.5 to 135,000 U $kg^{-1} h^{-1}$ for 10 days. This was followed by transfer of LAK cells from the peritoneum by Denver shunt. Mild pyrexia ($n = 7$), malaise ($n = 7$), anorexia and nausea ($n = 4$) were observed. There was no evidence of capillary leak syndrome, however, and all patients developed an early lymphopenia and anaemia which required transfusion of packed cells in five cases. Platelets and clotting activity remained normal but an eosinophilia and neutrophilia were observed. Lymphocyte subset markers and natural killer activity to K562 targets were studied serially during the 10-day infusion period by flow cytometric analysis. A significant increase in NK (CD16 +) and activated (CD25 +) cells was observed in the peritoneal cavity and NK cell activity increased in cells from both the peritoneal cavity (7–33%) and blood (10–15%). Using this new technique a LAK response may be induced within the peritoneal cavity and appears to be transfered into the systemic circulation. It is well tolerated compared to systemic immunotherapy using *in vitro* generated LAK and is effective at lower doses of rIL-2 compared to intravenous administration.

**Cisplatin invoked lead mobilisation studies**

R.P. Beaney, E.J. Buxton, A.M. El-Sharkawi, A.C. Todd, R.A. Braithwaite, L.J. Somervaille, D.R. Chettle, M.C. Scott, S.J. Jones, I.R. Hainsworth, I. Evetts, W.D. Morgan & C.J. Evans

*University College & Singleton Hospital, Swansea, and University of Birmingham and Regional Toxicology Laboratory, Dudley Road Hospital, Birmingham, UK.*

An earlier study of platinum uptake in the kidney following cisplatin chemotherapy reported, unexpectedly, very high kidney lead levels in four of 10 subjects, two of whom definitely had occupational lead exposure, one with exceptionally high bone lead, and a third with probable exposure. This gave rise to the hypothesis that cisplatin could mobilise lead from the skeleton. We report here two further, independent, investigations performed at Swansea and Birmingham, the object being to establish if lead mobilisation was a clinically significant hazard to patients treated with conventional doses of cisplatin. Each centre used a different X-ray fluorescence system to monitor renal lead levels on a total of 17 subjects (11 male and six female) undergoing cisplatin chemotherapy for a variety of solid tumours. Whole blood, serum and urine lead concentrations were also monitored before and after chemotherapy. In contrast to the earlier study, none of these subjects was known to be occupationally exposed. Pre-treatment values of whole blood and urine were all within their normal ranges for non-occupational exposed subjects ($<30\,\mu g\,dl^{-1}$ and $50\,\mu g\,24\,h^{-1}$ respectively), as were bone lead levels. Following chemotherapy, renal accumulation of lead was observed in only one subject, who showed transitory levels up to $100\,\mu g$ Pb $g^{-1}$ kidney tissue, much lower than the high levels reported previously. This is consistent with the blood, serum and urine analyses which, where above atomic absorption spectrometry detection limits, showed only minor changes in all subjects. We conclude that any lead mobilisation following cisplatin chemotherapy is unlikely to pose a major hazard to most patients, but that the possible significance for those exposed to lead and having high skeletal lead burdens remains to be determined.

**Novel epidermal growth factor receptor (EGFR) antibodies: laboratory selection procedures and clinical potential**

C.R. Hamilton, J. Styles & C. Dean

*Institute of Cancer Research and Royal Marsden Hospital, Sutton, Surrey, UK.*

Since January 1983, 409/552 patients presenting to the Royal Marsden Hospital with squamous cell carcinoma of the head and neck have been staged T any, N0, M0. 344 did not receive elective nodal irradiation (ENI) and of these 42 (12%) have suffered nodal relapse. As a preliminary to introducing a novel method of imaging head and neck cancer patients, and selecting those who might benefit from ENI a panel of rat monocolonal antibodies has been raised against the EGFR which is overexpressed in such patients.

Twelve EGFR antibodies have been raised via inoculation of the Peyer's patch of the rat with a human cell line (HNS) that over-expresses EGFR. Mesenteric node fusion with the rat Y3 myeloma cell line gave 12 hybridomas secreting appropriate antibodies.

Initial epitope mapping studies suggest at least three different binding sites are recognised including that of epidermal growth factor itself.

Scatchard analysis allows determination of antibody affinity constants ($\sim 10^8\,l\,mol^{-1}$) and ranking of affinity. Together with the epitope mapping studies appropriate antibody selection can be made for mouse xenograft work before final selection for the clinic.

**Augmentation of CEA expression in gastrointestinal cancer with theophylline and interferon**

A.J. Jewkes, B. Russell, C. Jones, D. Croom, W.H. Allum & J. Fielding

*Surgical Immunology Unit, Department of Surgery, Queen Elizabeth Hospital, Birmingham B15 2TH, UK.*

Heterogeneity of CEA expression occurs in most gastrointestinal cancers and agents which could render expression. more homogeneous are likely to increase the efficacy of antibody related procedures. Increased expression of several tumour associated antigens has been reported following treatment with theophylline or interferon.

We have therefore investigated the effects of these agents, *in vitro*, in four human colorectal cancer lines, a gastric cancer cell line and fibroblasts. The cell lines chosen cover a wide range of natural CEA expression. Cellular expression of CEA was estimated using a monoclonal anti-CEA antibody and flow cytometry. After 24 h treatment CEA levels were markedly increased ($P<0.001$) in two cell lines with interferon α and in three lines with theophylline. The effect was dose-dependent. Two cancer lines were unresponsive to treatment with either agent but both these had high levels of CEA initially. No CEA expression occurred in fibroblasts. This effect was then confirmed, *in vivo*, using xenografts of the various tumour lines grown subcutaneously in nude mice. CEA was estimated from whole tumour extracts by radioimmunoassay and the findings were comparable to *in vitro* studies.

Both agents can substantially increase CEA levels in some cancers cell lines especially those with low inherrent CEA expression. These drugs may therefore prove useful in improving the diagnosis and treatment of cancers with antibodies.

**Screening for cytotoxic-irradiation interactions in the mouse lung**

S.P. Lockhart, J.D. Down & G.G. Steel

*Radiotherapy Research Unit, Institute of Cancer Research, Sutton, UK.*

Irradiation of a 1.9 cm long mouse right hemithorax field (RHT) to 14 Gy and 18 Gy with 250 kV X-rays produced a rise in breathing rate which was dose-related and maximal at 14–18 weeks. To investigate the interaction with cytotoxic drugs, the maximally tolerated dose of cytotoxic drug was administered intraperitoneally 45 min before 14 Gy RHT irradiation. Cyclophosphamide $100\,mg\,kg^{-1}$ accentuated and accelerated the rise in breathing rate. Ifosfamide $300\,mg\,kg^{-1}$ had no such effect. BCNU delayed the rise in breathing rate to after 26 weeks. Busulphan $30\,mg\,kg^{-1}$ appeared to abolish the effect of irradiation, but this was due to the DMSO vehicle. The following cytotoxics had no effect; doxorubicin $6\,mg\,kg^{-1}$, carboplatin $100\,mg\,kg^{-1}$, vindesine $4\,mg\,kg^{-1}$, vinblastine $4\,mg\,kg^{-1}$.

In conclusion, this is a simple and convenient screening test for cytotoxic irradiation lung interactions, yielding qualitative information on the presence of an interaction, and further information on the time course of the response.

**The effect of hyperthermia on doxorubicin accumulation and cytotoxicity in a human ovarian carcinoma cell line and its multidrug resistant variant**

E.J. Osborne, S.P.C. Cole & W.J. Mackillop

*Department of Oncology, Queen's University, Kingston, Canada.*

The effect of temperature on doxorubicin (ADM) accumulation and cytotoxocity was studied *in vitro* in the human ovarian carcinoma cell line A2780-9S and in its multidrug-resistant variant A2780-AD645. Intracellular ADM levels were measured by flow cytometry and ADM cytotoxicity was measured using a clonogenic assay.

ADM cytotoxicity was markedly enhanced in both cell lines when cells were exposed to the drug at elevated temperatures. Initial rates of ADM influx were similar in the two cell lines and both lines showed a similar large increase in initial rate of influx with increasing temperature. Little efflux of ADM occurred in the sensitive cell line, but in the resistant cell line intracellular ADM levels decreased to approximately 50% of control at 37°C by 20 min. Efflux rates did not vary with temperature. Studies of the time course of ADM uptake showed that by 20 min the resistant cells had markedly lower intracellular ADM levels than the sensitive cells. Intracellular ADM levels after prolonged exposure to drugs were significantly increased at elevated temperatures in both cell lines.

It was concluded that the cytotoxicity of ADM is enhanced at elevated temperatures in both drug-resistant and drug-sensitive human ovarian carcinoma cells. The enhanced cytotoxicity is, in part, due to increased drug accumulation. The increase in intracellular drug levels is sufficient to explain the observed thermal enhancement of cytotoxicity in the A2780-9S cells.

**Effect of hypoxia on misonidazole binding in normal and tumour-bearing mice**

M.P. MacManus, A.P. Maxwell & W.P. Abram

*Queen's University of Belfast and Belvoir Park Hospital, Northern Ireland.*

Reductive metabolism of misonidazole (MISO) is enhanced by hypoxia and produces adducts which bind covalently to cellular macromolecules. Other workers assert that radioactive MISO can label hypoxic cells in tumours. We investigated the effect of hypoxia on the distribution of radiolabelled MISO in normal mice and in mice bearing CA NT or T50/80 carcinomas. MISO binding was investigated qualitatively by autoradiography and quantitatively by scintillation counting of solubilised organs or tumours. Mice were randomised to hypoxic or control groups and injected i.p. with labelled MISO. Immediately after MISO administration, mice in the hypoxic group were either exposed to hypobaric hypoxia (0.5 atmospheres) or given carbon monoxide i.p., producing $>50\%$ HbCO. Mice were killed 24 h after MISO administration, allowing excretion of unbound drug.

Hypobaric hypoxia increased binding in the T50/80 tumour by a factor of four but binding to CA NT did not change significantly. Hypobaric hypoxia significantly increased MISO binding to liver ($\times$ 2.5), kidney ($\times$2.4), heart ($\times$1.8) and spleen ($\times$2.9) Mice treated with CO also showed increased MISO binding in these organs. Autoradiographs of hypoxic liver showed a striking zonal distribution of MISO binding, suggesting that a steep oxygen tension gradient exists between portal tracts and hepatic veins. Autoradiographs of hypoxic kidney suggested that the existence of an oxygen tension gradient between tubules and glomeruli. This study confirms the value of MISO as a marker for cellular hypoxia. The demonstration of hepatic and renal oxygen tension gradients by MISO has not previously been reported.

**A new extracellular matrix proteoglycan recognised by a monoclonal antibody to human embryonal carcinoma**

M.D. Mason & M.F. Pera

*Institute of Cancer Research, Sutton, Surrey, UK.*

Recently the central importance of the extracellular matrix (ECM) in the control of growth and differentiation has begun to be appreciated. In particular, the proteoglycan content of the ECM is known to be of importance, for example in the attachment of differentiating cells to stromal cells, the binding of locally released growth factors, and the guiding of the normal pathway of migration in certain embryonic cells.

We report on a new extracellular matrix proteoglycan produced by human embryonal carcinoma cells (EC) and recognised by the monoclonal antibody GCTM-2. Immunohistochemistry shows that the antibody stains undifferentiated stem cells in alcohol and formalin fixed paraffin sections of human teratomas, and in seminomas. The epitope is also expressed strongly in fetal muscle, and in some epithelia, notably gut. An unusual feature of the ECM proteoglycan in teratoma cells is that the major carbohydrate is keratan sulphate, formerly thought to be restricted to cornea and cartilage.

The levels of expression of the antigen fall sharply during spontaneous differentiation of EC cells *in vitro*. This is in marked contrast to the situation in some normal tissues where highly differentiated tissues have the strongest levels of expression. An understanding of this phenomenon would improve our understanding of the control of growth and differentiation in normal and neoplastic cells.

**Determinants of cellular radiosensitivity. Induction *vs* repair**

T.J. McMillan, A.M. Cassoni, J.J. Eady, S. Edwards, A. Holmes & J.H. Peacock

*Radiotherapy Research Unit, Institute of Cancer Research, Sutton, Surrey, UK.*

The inherent radiosensitivity of cells is likely to be determined either by the amount of damage that is deposited by ionising radiation within critical targets in the cell or by the ability of the cell to repair this damage. In this study we have examined manifestations of these two processes in order to determine their relative importance in the determination of the sensitivity of a range of human tumour cell lines.

Repair capacity has been assessed using a viral reactivation assay in which cells are infected with irradiated virus and the ability of the cells to repair the damaged virus is measured by counting the number of cells containing proliferating virus. Five cell lines have been examined. Two radioresistant bladder carcinoma lines (MGH-U1 and RT112) proved to reactivate the virus to a greater extent than the more sensitive neuroblastoma lines (HX142 and HX138). However, the most radioresistant line, HeLA, was least proficient in the virus assay.

The level of induced DNA double strand breaks has been measured in nine human tumour cell lines using neutral filter elution. A broad spectrum of induction curves was found and it was evident that the level of damage was greater in the more sensitive cell lines.

We therefore conclude that while no simple relationship was found between repair capacity and sensitivity the level of induced damage was modified in line with sensitivity. Thus the level of induction may be a more significant determinant of radiosensitivity than repair in human tumour cells.

**A comparative study of the accessibility of tumour antigenic sites *in vitro* and *in vivo***

S. Pervez, S.C. Kirkland, A.A. Epenetos, D.J. Evans, W.J. Mooi & T. Krausz

*Departments of Histopathology and ICRF Oncology Group, Royal Postgraduate Medical School, Hammersmith Hospital, London W12 0HS, UK.*

The clinical usefulness of monoclonal antibodies as carriers of anti-tumour agents is presently being investigated in diagnostic as well as therapeutic fields, but little attention has been paid to the accessibility of antigenic sites in solid tumours.

To investigate this problem we used xenograft model systems of a poorly differentiated and a well differentiated adenocarcinomas of human large bowel origin. Former of these employs a cell line (LoVo), in which all tumour cells express a surface antigen reactive with the mouse monoclonal antibody AUA1. Tumour xenografts of LoVo (a poorly differentiated adenocarcinoma) were established in nude mice, accessibility of tumour cells to injected $^{125}$I-AUA1 and its F(ab')$_2$ fragments was investigated. In contrast to *in vitro* results, injected $^{125}$I-AUA1 was localised as detected on autoradiography, mainly at the peripheal portion of the tumour on a thin layer of tumour cells (3–5 cells) or adjacent to the vascularised stroma. On small microscopic sized tumour islands the antibody penetration was complete. Most of the radioantibody was found out of the blood vessels onto the tumour cells indicating adequate permeability of tumour vasculature for the macromolecules to diffuse out. With F(ab')2 fragments there was deeper penetration (5–10 cells) but less radioantibody deposition. The effect of time and amount of injected antibody on *in vivo* localisation was studied. It was demonstrated that at day 6 post-injection intact antibody showed similar uptake as on days 1 and 3 but in contrast F(ab')2 fragments showed a marked decrease in tumour uptake at day 6. By increasing the antibody dose no improvement in antibody penetration was seen. In another experiment for the first time the binding of *in vivo* injected antibody with tumour antigens was very precisely demonstrated immunohistochemically by biotinylating the injected antibody AUA1 instead of radiolabelling. *In vivo* localisation of antibody was demonstrated on histological sections of xenografts by incubation with avidin peroxidase.

In the other model, xenografts of a well differentiated adenocarcinoma cell line (HRA-19) and two monoclonal antibodies (AUA1 and HMFG1) which recognise antigens present on basolateral and apical membrane domains of polarised epithelial cells were used. When HRA-19 xenograft section stained *in vitro*, AUA1 stained only the basolateral domains of polarised tumour cells, while HMFG1 stained only the apical (luminal) domains of tumour cells within acini. $^{125}$I-AUA1 injected into nude mice bearing HRA-19 xenografts showed localisation on basally expressed antigen at the periphery of neoplastic glands, adjacent to the vascularised stroma. In contrast $^{125}$I-HMFG1 did not show any specific localisation as the apical (luminal) antigen recognised by this antibody was sealed off by junctional complexes between the cells.

Our study suggests that antibody guided therapy may be most effective in treating microscopic residual disease of micrometastases rather than symptomatic large tumours and that differentiation of the tumour and exact location of antigen sites are important factors which can affect the *in vivo* localisation of antibody.

**Predicting response of human tumours to radiotherapy using short-term culture and the MTT assay**

P. Price, T. McMillan, R. Wankling, C. Parkins & A. Horwich

*Radiotherapy Research Unit, Institute of Cancer Research and Royal Marsden Hospital, Sutton, Surrey, UK.*

Human tumour radiosensitivity, as measured by the surviving fraction at 2 Gy ($SF_2$), has been shown to correlate with clinical radioresponsiveness. Measurement of $SF_2$ values of individual tumour biopsies might therefore be useful in predicting response to radiotherapy. Traditionally $SF_2$ values are measured using clonogenic assays but a quicker and simpler assay is required for use in predictive testing. The MTT assay is a method of quantifying metabolically viable cells in culture through their ability to reduce tetrazolium to coloured formazan crystals. The crystals are dissolved and quantified by measuring optical density (OD). The relationship between OD and cell number is not linear and a calibration curve for each tumour is used. Using this measurement of cell number, growth curves of unirradiated and irradiated cells can be constructed and surviving fraction calculated. Using five human tumour cell lines a reproducible measure of survival was obtained and compared well with clonogenic survival measurements (in parentheses): Hx171 0.66 (0.69), Hx156 0.64 (0.62), RT112 0.75 (0.61), MGHU1 0.57 (0.57), Hx142 0.123 (0.109). The timing of calculating $SF_2$ is determined by the doubling time of the tumour cells. Four cell doublings are required to accurately assess 1 log of cell kill. This is ideal for a predictive assay of human tumour biopsies grown in short-term culture as $SF_2$ values can be calculated before fibroblasts overgrow the culture and can be available to the clinician before treatment. $SF_2$ values have been obtained from human tumour biopsies grown on plates coated with a cell attachment matrix (CAM, LifeTrac Inc.). This forms part of a prospective trial to correlate *in vitro* radiosensitivity with clinical radiocurability.

**DNA 'fingerprint' changes in patients on combination chemotherapy**

K. Ross, N. Haites, K. Kelly, A. Dawson & B. Bennett

*Departments of Medicine & Therapeutics and Genetics & Microbiology, University of Aberdeen, Aberdeen, UK.*

Hypervariable minisatellite DNA probes 33.6 and 33.15 have been employed in a novel way in a group of patients receiving combination chemotherapy in the treatment of lymphoma. The multi-locus nature of these probes allows simultaneous observation of over 60 different sites in human genomic DNA, enhancing the possibility of observing change.

In three patients out of 10 already studied, changes have been observed in their peripheral leukocyte-derived DNA fingerprint at some point after starting treatment. Changes involve both the loss of bands present in pre-treatment samples and the appearance of novel bands.

Care has been taken to consider possible mechanisms whereby the changes may have arisen spuriously, or may represent artefacts generated by the fingerprinting technique. We conclude that the changes observed are unlikely to represent any of these mechanisms, and that they may represent site-specific modification of the DNA by one or more of the agents used in chemotherapeutic regimes.

We will explore possible mechanisms by which these changes may have arisen, and discuss possible implications for risk of therapy-related malignancy

**Blood transfusion enhances colonic carcinogenesis in rats – does indomethacin prevent this?**

W.B. Ross, I. Nawroz, M.J. Beavis & J.R. Salaman

*Cardiff Royal Infirmary and Western General Hospital, Edinburgh, UK.*

Blood transfusion (BT) can cause immunosuppression and this may have a detrimental effect on survival and recurrence for patients with cancer. The mechanisms are unclear, but may involve increased synthesis of macrophage prostaglandin E2 and indomethacin may prevent this. Male Sprague–Dawley rats were given 14 weekly injections of dimethylhydrazine (16 mg $kg^{-1}$). During week 8 they were divided into four groups. Group A received no additional treatment. Group B were given three weekly 1.5 ml transfusions of heparinised blood from DA rats. Group C were given indomethacin in their drinking water for four weeks (2 mg $kg^{-1}$ $day^{-1}$). Group D were transfused with blood as in group B and given indomethacin to cover this period as in group C. The rats were killed at 28 weeks and colonic carcinogenesis assessed histologically: 342 tumours were identified of which 259 were adenocarcinomas and 83 were adenomas. The results confirm that BT enhances colonic carcinogenesis (A versus B: $P < 0.02$, Mann–Whitney test). This effect may be reversed by indomethacin, but comparison of groups B and D just failed to demonstrate a statistically significant difference.

| | *A* | *B* | *C* | *D* |
|---|---|---|---|---|
| Treatment group | none | BT | indomethacin | BT + indomethacin |
| Colonic tumours per rat, mean (*n*, s.d.) | 3.3 (17, 1.7) | 6.7 (19, 4.0) | 4.7 (16, 3.5) | 4.7 (18, 2.6) |

**The effect of oncogenes H-*ras* and c-*myc* on the radiosensitivity of a mink epithelial cell line**

J. Russell & D. Kerr

*Departments of Radiation Oncology and Medical Oncology, Beatson Oncology Centre, Belvidere Hospital, Glasgow, UK.*

We have investigated the effect of the oncogenes H-*ras* and c-*myc* on cellular radiosensitivity. A mink lung epithelial line was transfected to yield two sublines, H06T1 containing the H-*ras* oncogene and MCGM1 containing c-*myc*. At clinically relevant doses (200 cGy), cells transfected with c-*myc* showed a survival (68%) significantly greater than the parent line (53%). Cells tranfected with H-*ras* did not show enhanced survival of this dose. The survival curve for MCGM1 (for doses in the range 0–1000 cGy) showed a slightly increased shoulder in the low dose region relative to both CCL-64 and HOGT1. The width of this shoulder, the Dq, was 180 cGy for MCGM1 cells compared to 140 cGy for both GOGT1 and CCL-64. The terminal slope of the survival curve was slightly steeper for untransfected cells than for the transfected lines. Expressed as values of Do (the reciprocal of the slope) HOGT1 had a Do of 230 cGy,and MCGM1 200 cGy compared to a Do of 200 cGy for CCL-64. These results are consistent with a modest effect of some oncogenes on cellular radiosensitivity, especially in the low dose region.

**Successful application of a simple chemosensitivity test in the management of acute myeloid leukaemia**

J.M. Sargent, J.K. Wilson, A.W. Elgie & C.G. Taylor

*Pembury Hospital, Tunbridge Wells, UK.*

We describe a successful application of a rapid semi-automated colorimetric assay to aid the selection of cytotoxic drugs for individual patients with AML. Blast cells from bone marrow were incubated for 48 h with relevant types and concentrations of cytotoxic drugs. Cell survival after drug exposure was then calculated using the MTT assay by testing the ability of the cells to reduce the tetrazolium salt MTT to formazan. This non-clonogenic approach, measuring the total cell kill in the entire blast cell population, has been shown to be equally as valuable as other methods of chemosensitivity testing.

The amount of formazan produced corresponds to the number of cells plated. There was variation in drug sensitivity between patients. The assay can be used as a model to screen resistance modifiers and design combinations of cytotoxic drugs. Assay results correlated well with the clinical response after drug administration. Firstly, the attainment of complete or partial remission corresponded to the clinical outcome in 90% of cases tested. Secondly, the *in vitro* response paralleled the reduction in the number of blast cells in the peripheral blood in the 48 h following drug administration.

This method provides an early prediction of both sensitivity and resistance in individual patients. The repeatability and speed enables sequential testing throughout the course of the disease allowing for rapid response to changes in drug sensitivity.

**The clearance of $^{131}$I labelled murine monoclonal antibody from patient blood by intravenous human antimurine antibody**

J.S.W. Stewart, G.B. Sivolapenko, V. Hird, K.A.A. Davis & A.A. Epenetos

*Department of Radiotherapy, Hammersmith Hospital, Du Cane Road, London W12 0HS, UK.*

Five patients receiving intraperitoneal (i.p.) $^{131}$I labelled monoclonal antibody for ovarian cancer also received intravenous (i.v.) heterologous human anti-murine antibody (HAMA). It was intended that the i.v. HAMA would increase the catabolism and accelerate the clearance of absorbed $^{131}$I labelled MAB (thus decreasing the radiation dose to normal tissue). The pharmacokinetics of $^{131}$I labelled MAB in these patients was compared with that of 28 other HAMA negative receiving i.p. radiolabelled MAB for the first time.

Patients receiving i.v. HAMA demonstrated rapid clearance of $^{131}$I labelled MAB from their circulation. The (mean) maximum of $^{131}$I blood content was 11.4% of the injected activity in patients receiving HAMA compared to 23.3% in patients not given HAMA. Intravenous HAMA decreased the radiation dose to bone marrow (from I-131 labelled MAB in the circulation) four-fold. $^{131}$I MAB/HAMA immune complexes were rapidly transported to the liver following the injection of HAMA. Hepatic antibody dehalogenation was rapid with 87% of the injected $^{131}$I excreted in 5 days. Despite the efficient hepatic clearance of immune complexes, dehalogenation of MAB was so rapid, that the radiation dose to liver parenchyma from circulation $^{131}$I, was decreased four-fold rather than increased. There was no evidence of passive immunisation to HAMA, and all patients developed homologous two to four weeks after treatment.

**The influence of relative timing of carcinogen and operation on the appearance and distribution of colonic tumours in the rat model**

M. Tilston, A. Abou-Zeid, E. Grant & M.R.B. Keighley

*The General Hospital, Birmingham, UK.*

Local recurrence of large bowel cancer frequently occurs adjacent to the site of an intestinal anastomosis, and may be due to a biological abnormality associated with anastomotic healing. We have studied the last hypothesis by observing the yield of tumours at a colonic anastomosis in the DMH rat polyp/cancer sequence model. One hundred and twenty rats received a 5 week course of weekly injections of dimethylhydrazine (40 mg $kg^{-1}$) subcutaneously. They were divided into three surgical groups: left colotomy, partial left colectomy and sham laparotomy, and each group was further subdivided to receive DMH either 2 weeks before or two weeks after their laparotomy. All groups were sacrificed 25 weeks after commencement of the DMH course. Tumour counts were taken either at, or away from the anastomotic site (see Table).

Colonic operation increased the percentage of rats developing tumours. A high yield of tumours was observed adjacent to a colonic anastomosis, especially when a healing suture was challenged by exposure to a carcinogen.

| | *Laparotomy* | | *Colectomy* | | *Colotomy* | |
|---|---|---|---|---|---|---|
| | *Preop.* | *Postop.* | *Preop.* | *Postop.* | *Preop.* | *Postop.* |
| % rats with tumours | 30% | 25% | 75%* | 75%* | 80%* | 85%* |
| Anastomotic tumours | 1 | 0 | 11 | 16 | 23 | 17 |
| Rest of colon | 7 | 5 | 14 | 7 | 8 | 18 |
| Total | 8 | 5 | 25 | 23 | 31 | 35 |

*$P < 0.05$.

### Photodynamic therapy using polychromatic light for the growth inhibition of multicellular tumour spheroids

M. Turkish, P. Durdey, M.F. Grahn, L. Ilincic, J.T. Allardice & N.S. Williams

*Surgical Unit, London Hospital, Whitechapel, London E1, UK.*

Photodynamic therapy (PDT) is receiving increasing attention as a treatment for malignant disease. The cost and maintenance of lasers is, however, prohibitive and this may restrict the use of PDT in patients who are otherwise suitable. We have therefore investigated alternative non-coherent light sources to determine whether a comparable cytotoxic effect can be achieved over similar time periods and irradiation doses. Red and green laser light were compared to theatre lights and daylight tungsten lamps. Multicellular tumour spheroids (MTS) from the cell line HRT18 were pre-treated with $10\,\mu g\,ml^{-1}$ of haematoporphyrin derivative and the cytotoxic effect of different light sources was measured using tritiated thymidine incorporation. Results are expressed as mean ($\pm$ s.e.m.) percentage inhibition.

| | *Irradiance ($J\,cm^{-2}$)* | *% inhibition* |
|---|---|---|
| Red laser light | 10 | 54.7 ($\pm$ 6)$^{a,b}$ |
| Theatre lights | 10 | 87.0 ($\pm$ 3.7)$^{a}$ |
| Tungsten daylight lamps | 4.25 | 70.4 ($\pm$ 6.1)$^{b}$ |

$^{a,b}P < 0.01$ (ANOVA).

For a given irradiance, growth inhibition of MTS was significantly greater with a polychromatic light source than laser light. Polychromatic light may not achieve as effective tissue penetration as does red light. However, for the ablation of superficial tumours and adjuvant treatment of the tumour bed peroperatively, the cheaper alternative of an adapted polychromatic light source may allow more widespread use in clinical practice.

### Sarcomas and miscellaneous

### The application of printed circuit technology to produce a multichannel probe to measure oxygen

A.E. Brewster, I.C.M. Paterson, J.L. Moore, P. Waller & R.S. Pickard

*Velindre Hospital, Cardiff and University of Wales College of Cardiff, UK.*

Data about hypoxia fractions in human tumours is circumstantial because of the limitations of current methods for measuring oxygen. A prototype printed circuit was designed with the dual intention of establishing whether: (1) the materials and fabrication techniques required were biocompatible and (2) such a circuit would be capable of measuring a biologically generated potential.

The circuit, etched on glass so that the growing cells could be visualised, was designed with 52 electrodes, $4 \times 4\,\mu m$ size. They were arranged in quadrants $20\,\mu m$ apart to correspond with the approximate size of tumour cells in culture. Three cell lines, Chinese hamster ovary, a mouse fibroblast and a mouse neuroblastoma line will grow normally on the circuits as demonstrated by standard growth curves. S.e.m.s will be presented showing the complexity of the cell/sensor interface.

To test the circuit's ability to pick up a biologically generated signal frog cardiac muscle which was visibly contracting was placed onto the circuit. Cardiac potentials were recorded from each terminal providing data from which the signal/noise ratio could be calculated.

We have therefore integrated 21 oxygen sensors and three thermistors into the circuit to measure oxygen *in vitro* and have designed a multichannel silicon probe for *in vivo* use. This probe will be implanted into a growing tumour to build up a dynamic three dimensional blueprint of oxygen and temperature.

### Topical 5-fluorouracil in cutaneous AIDS-related Kaposi's sarcoma

A.M. Brunt, A.G. Goodman & R.H. Phillips

*Radiotherapy Dept, St Stephens and Westminster Hospitals, London, UK.*

Five per cent 5-fluorouracil cream (Efudix) is used to treat selected neoplastic and pre-neoplastic cutaneous lesions. A frequent indication for treating cutaneous AIDS-related Kaposi's sarcoma is for cosmesis. Localised lesions are often treated by radiotherapy or by intralesional cytotoxic chemotherapy. The self application of a cream to treat early cosmetically unacceptable sites of disease would be an attractive therapy for this group of highly motivated patients. We are conducting a pilot study of 5% 5-fluorouracil cream in patients with AIDS-related Kaposi's sarcoma. All patients have two similar early lesions identified and randomly allocated to treatment with the cream or assessment as the control. The cream is applied once daily for 1 month, and then twice daily until 3 months, unless ulceration occurs before this time. Ten patients have been recruited. Three are still applying the cream after 6, 6 and 8 weeks with no response to date. Three patients completed 3 months treatment, two achieving partial responses maintained at 1 month. Four patients did not complete 3 months application. One patient died at 3 weeks, two stopped due to intercurrent opportunistic infection after 6 and 4 weeks, the other patient admitted poor compliance. The patient who stopped at 4 weeks exhibits a partial response maintained at 2 months. Erythema was observed in no patient. Further study of this convenient treatment is recommended to determine whether it has a role in the management of AIDS-related Kaposi's sarcoma.

### The effect of radiotherapy on local recurrence in Ewing's sarcoma of bone

N.G. Burnet & C.L. Harmer

*Royal Marsden Hospital, London SW3, UK.*

To assess local control, 82 cases of Ewing's sarcoma of bone treated between 1967 and 1986 were reviewed. Forty-four males and 38 females were treated, median age 16 years. Thirty tumours arose in the leg, 15 in the arm, 16 in the pelvis and 21 elsewhere. Size could not be accurately assessed retrospectively. Sixty-two (76%) have relapsed, median time to relapse 12 months. Of the 20 (24%) disease-free, two died from complications of chemotherapy; median follow-up was 40 months but two patients had follow-up of less than 12 months. In addition to the 20 relapse-free patients, 38 relapsed distantly but with local control. Fifty-eight patients (71%) thus achieved local control. Eleven patients suffered local relapse alone, 13 local and distant relapse together. Most patients received both radio- and chemotherapy. Eleven patients received no radiotherapy, 10 no chemotherapy, and one received neither. Eleven patients had surgery, eight narrow excision and three wide clearance by amputation.

Proximal or distal location in a limb had no effect on relapse-free rates, which were similar (27%) for all areas except the pelvis, where only two of 16 (13%) were disease-free and both died from treatment complications. Four disease-free patients, including the two who died, had distant disease at presentation. However, no patient was salvaged after first relapse. More patients treated after 1976 remain disease-free (30%:9%). Fewer local recurrences were seen with radiation doses of 1,572 ret or more (equivalent to 50 Gy in 25 daily fractions), but there was little or no gain from higher doses.

### Chemotherapy-induced aortic damage

I.J. Neilly, M.A. Ratcliffe, J. Rawles, A.A. Dawson & B. Bennett

*Department of Medicine, University of Aberdeen and Aberdeen Royal Infirmary.*

Cytotoxic chemotherapeutic agents, particularly the anthracyclines, are known to be cardiotoxic, but toxic effects on the aorta have not previously been documented.

In this study diameters of ascending and descending thoracic aorta were measured by computerised tomography in 69 patients with lymphoma before and after first line treatment with one of seven different regimes.

Minor increases in aortic diameter over the period were expected due to the ageing process. These increases were greatly exceeded in both ascending and descending aorta after chemotherapy witdh CHOP and CVP regimes. Smaller changes, or changes which were not statistically significant, were noted after MVPP, ChlVPP, ChlVP, mediastinal radiotherapy and radiotherapy plus MVPP.

Cardiovascular damage associated with certain forms of cytotoxic therapy is not confined to the heart but also affects the aorta. This damage to the aorta may clearly be of clinical importance in the older patients receiving chemotherapy. This is illustrated by reference to patients, requiring aortic surgery during or shortly after chemotherapy.

### Differential inhibition of tumour and ephiphyseal growth seen in two cases of osteosarcoma of femur

J.A.S. Pringle, H.B.S. Kemp, R.L. Souhami & J. Pritchard

*London Supra-regional Bone Tumour Service, London, UK.*

Two males, aged 4 and 11 respectively, presented with osteosarcoma of the distal femur, with quite extensive involvement of the femoral shaft. Both were treated with prospective chemotherapy, resection of the whole femur and replacement by a growing endoprothesis. Good tumour response to chemotherapy was seen in both cases. Evidence of continued epiphyseal growth during chemotherapy was noted in gross specimen, fine detail X-ray and histological sections.

### A case–control study of leukaemia and lymphoma in young persons resident in West Cumbria

M.J. Gardner, M.P. Snee, A.J. Hall, S. Downes, C.A. Powell & J.D.T. Terrell

*MRC Environmental Epidemiology Unit, University of Southampton and West Cumbria Health Authority, Whitehaven, Cumbria, UK.*

Several studies have shown increases in the incidence of leukaemia in young persons living close to British nuclear installations. However, no link has been established between the occurrence of such cases of leukaemia and the operation of any of the nuclear plants.

The West Cumbria case–control study seeks to examine whether the increased incidence of lymphoid malignancy, seen in part of the area, can be explained on the basis of increased prevalence of known risk factors, or alternatively establish that the observed cases of malignancy are due, in part, to the activities of the largest nuclear site in the country: Sellafield. Some 97 cases of leukaemia and lymphoma, diagnosed in a 35-year period from 1950, in West Cumbria have been compared with 1,001 age and sex matched controls. Putative risk factors that may have resulted in enhanced irradiation from the Sellafield nuclides, such as place of birth, consumption of seafood and beach activity have been examined.

A particular feature of the study is the examination of paternal radiation exposure as a possible aetiological factor; the particulars of all study subjects have been cross-linked with the occupational records of persons who have ever worked at the plant. BNFL have supplied us with radiation dosimetry for linked individuals.

The preliminary results of the study will be discussed.

### Are you being served? British Oncology Data Managers' Association (BODMA)

T. Young (on behalf of the BODMA committee)

*Meyerstein Institute of Radiotherapy & Oncology, Middlesex Hospital, London, UK.*

BODMA was founded in December 1987 to represent oncology data managers and clinical trial co-ordinators in the UK and to organise national meetings and regional workshops. A recent CRC report recommended that a training programme and career structure should be established.

Although some data managers now work in units funded by the CRC and MRC dedicated to conducting clinical studies, many still work alone. One of the association's first aims is therefore to identify and contact all data managers. Current membership is 116: Scotland and NE 22, Manchester and NW 11, Yorkshire 6, Midlands 19, Plymouth 2, London 56. Wales and East Midlands are poorly represented.

The Scottish region organised the first national meeting in Glasgow last autumn. Topics covered included basic cancer pathology, statistics, protocol and form design, computer software packages and the Data Protection Act. The London and SE region has drawn up a questionnaire to provide baseline information on job descriptions, responsibilities and experience which will be used when planning future meetings. Results from this survey should be available for presentation soon. Information on disease sites of interest and computing facilities could be used for collaboration between centres. The association is co-operating with the EORTC Data Managers' Group and learning from European experience.

Clinical studies are important. More efficient use of data managers would help to optimise clinical research, locally, nationally and internationally.

### Breast cancer

### Effervescent oral APD treatment of bone metastases from breast cancer

R.E. Coleman, P.J. Woll & R.D. Rubens

*ICRF Clinical Oncology Unit, Guy's Hospital, London, UK.*

The bisphosphonate APD (Pamidronate) is a potent inhibitor of osteolysis and the most effective agent for the treatment of hypercalcaemia of malignancy. Long-term inhibition of bone destruction is possible with repeated intravenous administration and this may reduce pain and promote bone healing. In the past the use of oral APD has been limited by upper GI intolerance due to the drug's corrosive properties. We have tested a novel effervescent formulation of oral APD (provided by Ciba-Geigy Pharmaceuticals).

Thirteen patients with bone metastases from breast cancer were studied, eleven with evidence of increased osteolysis (urinary calcium excretion $> 0.4\,\mathrm{mmol\,mmol^{-1}}$ creatinine).

The first four patients were treated with 150 mg daily for 4 weeks. Subsequent cohorts of four patients received 300 mg and 450 mg daily. Dose escalation within patients was allowed only after completion of four weeks treatment at a constant dose. APD was tolerated without toxicity up to a dose of 450 mg a day. In two pts dose escalation to 600 mg a day resulted in unacceptable nausea, vomiting and indigestion. All patients showed a fall in urinary calcium excretion to normal indicating adequate absorption of APD and inhibition of bone resorption at all dosages. Serum calcium fell significantly but remained within the normal range.

Effervescent oral APD inhibited bone resorption secondary to breast cancer at doses which were non-toxic. Studies of long-term tolerance of oral APD and its use as an adjunct to systemic treatment are now indicated.

### *In vivo* localised $^{31}$P spectroscopy of human breast carcinoma

J. Glaholm, J.C. Sharp, D. Collins, N.P. Rowell, M.O. Leach, K. Farnsworth, V.R. McCready & A.J. Hind

*Nuclear Magnetic Resonance Unit, Royal Marsden Hospital, Sutton and Siemens Ltd, Sunbury-on-Thames, UK.*

*In vivo* $^{31}$P MRS provides a non-invasive measurement of cellular phosphorous metabolism. Eight patients with locally advanced breast carcinoma hve been assessed during treatment. Phosphomonoesters (PME) and phosphodiesters (PDE) (precursors and degradation products of phospholipid metabolism respectively) were prominent in all of the tumours. The technique may therefore provide an indirect indicator of cell membrane synthesis and hence replication. Phosphocreatine (PCr), which provides a reserve of high energy phosphate, was undetectable or at a low level in all of the tumours examined. Adenosine triphosphate (ATP) and inoganic phosphate (Pi) were readily detected in all cases providing an indication of the efficiency of cellular bioenergetic metabolism.

Five patients were treated with combination chemotherapy, two with tamoxifen and one with external beam irradiation. Three patients responding to chemotherapy demonstrated spectral changes, predominantly reduction of the PME and PDE peaks, bioenergetic changes (ATP, Pi, PCr) were less consistent. In two patients these changes preceded the clinical response. No spectral changes were observed in the remaining two non-responding patients. One of the tamoxifen treated patients showed neither spectral nor clinical response to therapy. The second demonstrated a generalised increase of the NMR signal two weeks after commencing tamoxifen with concomitant increase in tumour blood flow as measured by $^{99}$Tc$^{m}$ labelled HMPAO. These changes preceded an increase in the tumour volume and were therefore early markers of disease progression. No spectral changes were observed during treatment in the patient undergoing radical external beam breast irradiation, despite a reduction of tumour volume.

In conclusion we have observed spectral changes which preceded clinically measurable changes in tumour volume. *In vivo* $^{31}$P NMR spectroscopy may therefore provide a sensitive non-invasive method for assessing therapeutic response in the treatment of breast cancer.

### How far do staging investigations in breast cancer aid the clinician?

R. Glynne-Jones, A. Ahmed, T. Young & P.J. Ell

*Meyerstein Institute of Radiotherapy and Institute of Nuclear Medicine, University and Middlesex School of Medicine, London, UK.*

This retrospective study aimed to assess the clinical importance of current staging procedures in breast cancer. The analysis comprises a 10-year cohort of all 398 women who presented to a single unit between 1978 and 1987, with clinically staged I–III breast cancer. Routine screening for metastases yielded 72/389 $^{99}$Tc$^{m}$ bone scans, 13/386 chest radiographs and 21/271 liver ultrasound scans, which were retrospectively classified by their original report as equivocally or definitely indicative of metastases. In addition, 13/347 serum alkaline phosphatase levels were elevated beyond the normal range of the institutions concerned.

Further history, advancing disease, local radiographs or a repreat scan gave information which reduced the overall rate of detection of metastatic disease at three months to 29/389 (7%) bone scans, 10/386 (2.6%) for chest radiographs, 8/271 (3%) for liver ultrasound and 3/347 (1%) for serum alkaline phosphatase.

### The risk of occult invasive cancer of the breast following excision biopsy showing *in situ* ductal carcinoma of comedo pattern

P.D.J. Hardman & A. Worth

*The Cancer Control Agency of British Columbia, Vancouver, BC, Canada.*

During the period 1 January 1985 to 31 August 1987, 62 cases of *in situ* ductal carcinoma of predominant comedo pattern were diagnosed in British Columbia, following excision biopsy for a palpable or mammographically demonstrable lesion of the breast. The excision biopsies were each performed with the intention of removing the lesion completely. Fifty-seven (92%) underwent subsequent wide re-excision or total mastectomy, usually within one calendar month of the excision biopsy. Occult invasive disease was demonstrated in 14 of the subsequent wider excision specimens (24.5%) and residual *in situ* carcinoma was present in a further 24 (42.1%) cases. Therefore, the total incidence of residual disease was 66.6%.

Axillary lymph node sampling was performed in 54 cases. Lymph node metastases were observed in two cases (3.7%) and were each associated with occult infiltrating ductal carcinoma in the breast.

This suggests that *in situ* ductal carcinoma of predominant comedo pattern may be more widespread and associated with a higher incidence of invasive ductal carcinoma than previously accepted.

### Adjuvant chemotherapy for breast cancer: what is the cost?

R. Kalra, A.C. Jones, M. Greenall & K.R. Durrant

*Departments of Radiotherapy and Surgery, Churchill Hospital, Oxford, UK.*

An overview has demonstrated a significant difference in survival for adjuvant chemotherapy in women under the age of 50. However, there may be a considerable cost for this improvement in terms of patient side effects and financial resources. A study was initiated to assess the toxicity and quality of life associated with adjuvant chemotherapy.

Between January 1986 and December 1988, 69 premenopausal patients were treated by simple mastectomy or local wide excision and axillary node sampling followed by six cycles of CMF chemotherapy (Cyclophosphamide 400 mg m$^{-2}$, Methotrexate 40 mg m$^{-2}$ and 5-fluorouracil 600 mg m$^{-2}$ i.v. days 1 and 8). Axillary ± chest wall or breast irradiation was given after the second cycle of chemotherapy. Fifty-five patients have completed treatment and are evaluable.

Most patients experienced nausea and vomiting; in the majority anti emetics were ineffective. Hair loss was usually mild and only three patients required a wig. Anxiety and depression were reported by 32 patients.

Thirty-five patients were unable to complete six cycles of chemotherapy. This was due to neutropenia in 13 patients and 12 patients refused to continue chemotherapy after a mean of three and a half cycles.

The reduction in mortality for adjuvant chemotherapy is associated with unpleasant side-effects. A major priority must be the reduction in the toxicity of this otherwise valuable treatment.

**Efficacy and toxicity of single agent chemotherapy in advanced breast carcinoma**

P.A. Lawton, M.J. Ostrowski, T. Young & M.F. Spittle

*The Middlesex Hospital, London, UK.*

In 1985 the Multicentre Cancer Chemotherapy Group commenced a randomised study to compare doxorubicin ($70\,mg\,m^{-2}$), epirubicin ($70\,mg\,m^{-2}$) and mitozantrone ($14\,mg\,m^{-2}$) as single agents in patients with metastatic breast carcinoma. The patients had received no previous chemotherapy for their metastatic disease. Eighty-eight patients were entered and 87 were evaluable. The survival of all the patients was 32% at 1 year and 9% at 2 years with no significant difference in the three groups. The response rates were 40% (10 of 28 patients) with doxorubicin, 36% (nine of 28 patients) with epirubicin and 26% (eight of 31 patients) with mitozantrone but these differences did not reach statistical significance. Using WHO scores, 21% of patients with doxorubicin and 20% with epirubicin had grade 3 nausea/vomiting as their worst score compared with none with mitozantrone. However, 32% of the patients treated with mitozantrone did have grade 2 nausea/vomiting. WHO scores for alopecia were grade 3 for 57% of the patients doxorubicin compared with 31% with epirubicin and 3% with mitozantrone. Myelosuppression and infective episodes occurred more frequently in the patients receiving mitozantrone (14% of the total courses of mitozantrone were delayed due to neutropenia compared with only 7% for doxorubicin and 3% for epirubicin). Two cardiac complications were reported (angina in one patient on doxorubicin and a cardiomyopathy in one patient after 10 courses of epirubicin). This study shows that the low efficacy and toxicity of all three agents limit their use as single agents in advanced breast carcinoma and in the case of mitozantrone improved patient tolerance may be balanced by an increased risk of myelosuppression.

**Treatment of advanced poor-prognosis breast cancer in young women with doxorubicin plus ifosfamide/mesna**

M.J. Millward & B.M.J. Cantwell

*Department of Clinical Oncology, University of Newcastle upon Tyne, Newcastle upon Tyne, UK.*

Younger women with advanced breast cancer frequently present with poor prognostic features such as visceral disease and inflammatory local recurrences. We have treated 20 such patients (pts) aged $< 50$ years (mean age 42 years) who had not received chemotherapy (16 pts) or had progressive disease on mitoxantrone (four pts). To give high doses of alkylating drugs with the standard most active single agent (doxorubicin) we combined $40\,mg\,m^{-2}$ bolus followed by ifosfamide $5\,mg\,m^{-2}$ over 24 h with concomitant mesna. Starting doses were reduced in five pts because of previous radiotherapy, poor performance status or advanced liver involvement. Cycles are repeated every 3 weeks to a maximum of 4. Results are; CR four pts, PR eight pts, SD three pts, PD two pts, too early three pts, overall response rate 71%. Responses have been seen in local recurrence, liver, lung and pleural disease and in patients resistant to mitoxantrone. The median duration of response is 7 months. Eight relapsed or non-responding patients have received salvage chemotherapy with mitomycin-C and vincristine with two responses. The overall median survival is 12 months. WHO grade 3 myelosuppression occurred in five pts but no infectious complications have been seen. Falling creatinine clearance has lead to discontinuation of ifosfamide in two pts. One episode of haemorrhagic cystitis occurred after four cycles of therapy. Doxorubicin/ifosfamide is a highly active regimen in these young, poor prognosis patients and the high degree of antitumour activity may be explained by tumour kinetic parameters, but intrinsic drug resistance remains a problem and long-term survival is poor. The incorporation of these drugs in an alternating schedule with a mitomycin-C based regimen appears worthy of investigation.

**Radiation-induced brachial plexus injury: follow-up of two different fractionation schedules**

S. Powell & J. Cooke

*Royal Marsden Hospital, London, UK.*

All 449 breast cancer patients treated with postoperative radiotherapy to the breast and lymph nodes between 1982 and 1984 have been followed for 4–6 years. In this group two different fractionation schedules were used, one five times a fortnight and one daily, both over 6 weeks. The calculated dose to the brachial plexus was 45 Gy in 15 fractions or 54 Gy in 30 fractions. These schedules are equivalent doses using the NSD formula. The diagnosis of a brachial plexus injury was made clinically and computed tomography was used to distinguish radiation injury from recurrent disease.

The actuarial incidence of a radiation induced brachial plexus injury for the whole group was 4.9% at 6 years. No cases were seen in the first 10 months following radiotherapy. The incidence rises between 1 and 4 years and then starts to plateau. When the large fraction size group is compared with the small fraction size group the incidence at 6 years is 5.9% and 1.0% respectively ($P < 0.1$). Two different treatment techniques were used in this group but were not found to contribute to the probability of developing plexus injury.

It is concluded that radiation using large doses per fraction are less well tolerated by the brachial plexus than small doses per fraction, and a commonly used fractionation schedule such as 45 Gy in 15 fractions may give unacceptably high brachial plexus morbidity. The use of small doses per fraction or avoiding lymphatic irradiation is advocated.

### Genito-urinary and gynaecological cancer

**Bleomycin, ifosfamide and cisplatin (BIP) in cervical cancer: results in 100 cases**

E.J. Buxton, G. Blackledge, J.J. Mould, J.S. Tobias, J. Monaghan, D. Spooner, A.D. Chetiyawardana & A. Slater

*West Midlands Cancer Research Campaign Clinical Trials Unit, Birmingham, University College Hospital, London and Queen Elizabeth Hospital, Gateshead, UK.*

Patients with recurrent, advanced and early stage but high risk/node positive carcinoma of the cervix have an unfavourable prognosis. Systemic therapy may improve prognosis as neoadjuvant, cytoreductive or adjuvant therapy in primary disease. For maximum benefit systemic therapy should have high activity. We have treated 100 patients with BIP. Fifty patients with recurrent inoperable disease entered a phase II study of this combination. Fifty patients with primary inoperable disease also received BIP as cytoreductive therapy prior to radical local radiotherapy. Patients were treated with B 30 mg infused over 24 h, followed by P 50 mg $m^{-2}$ bolus and I 5 g $m^{-2}$ infused over 24 h, with concomitant hydration (total 8 litres in 3 days). Mesna 8 g $m^{-2}$ was given as a systemic uroprotector during and for 12 h following I. At least two cycles were given unless there was disease progression with a maximum in responding patients of six cycles in the recurrent group and three cycles in the primary group.

Seventy of 99 patients (71%) responded (95% CI 62–80%). In the recurrent disease group 34/49 patients (69%) showed objective response with 10 complete responses (22%). Median response duration was 8.4 months. All patients experienced alopecia, and nausea and vomiting. Blood transfusion was required in 23% of cycles. WHO grade 3/4 infection occurred in 5% of courses. Grade 3/4 CNS toxicity was seen in 7% of courses. Two patients developed grade 3/4 renal toxicity. In the neoadjuvant group 36/50 patients (72%) showed significant tumour regression. Only three patients showed disease progression during chemotherapy and these had progression during subsequent radical radiotherapy. There was no evidence of enhanced acute radiotherapy toxicity.

BIP is highly active in cervix cancer and may be used for effective cytoreduction in around 70% of patients. The role of BIP as cytoreductive therapy before radical radiotherapy in primary inoperable disease is currently being tested in a multicentre randomised trial. Eighty patients have entered this study. The regimen also has potential as adjuvant therapy in early stage 'high risk' disease. We have initiated a prospective randomised trial testing the role of BIP as adjuvant therapy in patients with positive nodes at radical hysterectomy.

### Staging of prostatic cancer: MRI, CT and transrectal ultrasound experience

M. Cobby, D. Gillat, J. Browning & E. Whipp

*Bristol Radiotherapy Centre and Bristol Royal Infirmary, Bristol, UK.*

Many patients with prostatic carcinoma will present when metastatic spread has already occurred. However, it is often diagnosed incidently following transurethral resection for outflow obstruction when the disease may be confined to the gland. These patients are suitable for possible curative treatments such as radioactive seed implants or radical prostatectomy. For these to be considered accurate preoperative staging is required.

Using a variety of techniques we have staged a series of 100 patients with histologically proven prostatic carcinoma. In 25 patients endorectal ultrasound was compared with examination under anaesthesia and fine needle biopsy where appropriate, before the insertion of $^{125}I$ seeds. Comparison was made with clinical examination and, where possible, with endorectally guided biopsy or surgically excised specimens in the remaining patients, 50 of whom underwent endorectal ultrasound and 25 a combination of endorectal ultrasound and magnetic resonance imaging. Computed tomography was performed on a number of patients from each group.

Transrectal ultrasound was accurate in assessing capsular breach and this was identified more frequently than by MRI. However, MRI more clearly showed the full extent of local invasion and was better at examining the bladder base. It also revealed lymphadenopathy and unsuspected pelvic bony metastasis in a number of patients.

### Preoperative assessment of myometrial invasion by vaginal endosonography

D.J. Cruickshank, J.M. Randall & I.D. Miller

*University of Aberdeen Medical School, Foresterhill, Aberdeen, UK.*

Endometrial cancer is the most common gynaecological malignancy and over the last 20 years there have been no improvements in the cure rate. Of the major prognostic factors in endometrial cancer only clinical stage histological tumour type and degree of differentiation are available preoperatively. More accurate preoperative staging, together with an assessment of myometrial invasion, cervical involvement and possibly adnexal spread would have the potential to improve management in endometrial cancer. In this pilot study preoperative transvaginal endosonographic assessment of myometrial invasion was compared with histological measurement in five consecutive patients with endometrial neoplasia. Preoperative ultrasound findings and postoperative microscopic assessment were in good agreement. Departure from the 'gold standard' of microscopic assessment was much less for vaginal ultrasound (− 21% to + 6%) than for macroscopic assessment (− 86% to + 18%). Selective therapy based on preoperative information on available prognostic factors would be expected to improve the results of treatment but perhaps more importantly minimise the morbidity and mortality by permitting conservative but effective surgery in this group of patients who tend to be elderly, obese and unfit.

### The cost effectiveness of serum CA125 monitoring in epithelial ovarian cancer

D.J. Cruickshank & J. Cairns

*Aberdeen University, Aberdeen, UK.*

The aim of this study is to evaluate whether the improved information derived from the use of CA125 in monitoring ovarian cancer has value in excess of its cost. A conventional monitoring scenario utilising ultrasound is compared with two new management scenarios, one where CA125 supplements the use of ultrasound and another where CA125 is used instead of conventional monitoring. Data from the literature on sensitivity, specificity and predictive value of the different means of assessing response is used to provide illustrative costs and benefits. The demonstration of a lead time and the superior sensitivity of CA125 monitoring over clinical assessment of response in studies of second look surgery suggest that serial CA125 measurement is applicable in non-evaluable disease (60%). Unnecessary second look surgery could be avoided and the earlier detection of progressive disease indicating ineffective chemotherapy would yield benefits by a reduction in the number of pulses of chemotherapy saving the health service resources and reducing morbidity associated with chemotherapy. At best, the response rate in advanced ovarian cancer is 74%. At least 26% of patients therefore receive chemotherapy unnecessarily. The earlier detection of recurrence after surgery with CA125 in patients with early stage disease not previously treated might be expected to improve survival.

### Immunoscintigraphy of ovarian carcinoma

B. Haylock, T. Maughan, M. Hayward, P. Facey, M. Shelley, R. Fish & M. Adams

*South Wales Radiotherapy and Oncology Service, Velindre Hospital, Cardiff, UK.*

CA-125 is a membrane antigen detected in tumour sections and serum from the majority of patients (pts) with adenocarcinoma of the ovary. We have attempted to use a radioiodinated anti-CA125 monoclonal antibody, OC-125, to image ovarian tumours using immunoscintigraphy and compare the findings with clinical examination and existing radiological techniques.

Thirteen scans were performed in 11 pts with FIGO stage III A-C and IV ovarian adenocarcinoma. At the time of scanning 11 pts had residual macroscopic disease (eight pts $<2$ cm, three pts $>2$ cm). One hundred and eleven MBq of $^{131}$I OC-125 was given i.v. 4 days before scanning with an IGE 400 T camera (EL-Scintapex 009 data processor) Subtraction films were obtained using Tc04 in a dual isotope technique.

Clinical examination was positive in 3/11 pts with disease. Ultrasound scanning yielded 3/10 true positive and 2/10 equivocal results. CT scanning showed true positive in 5/11 cases and 5/11 equivocal results. Considering pelvic and peritoneal sites separately, immunoscintigraphy gave 9/13 true positive, 4/13 false negative and 2/2 true negative results. Serum CA-125 levels were elevated ($>35$ units ml$^{-1}$) in 88% pts with macroscopic residual tumour.

This pilot study suggests that immunoscintigraphy will recognise residual tumour when clinical examination and other radiological investigations fail.

**Neoadjuvant chemotherapy before radical radiotherapy for transitional cell carcinoma of the bladder**

G.C.W. Howard, M.A. Cornbleet, T.B. Hargreave & G. Chisholm

*Department of Clinical Oncology and Department of Urology, Western General Hospital, Edinburgh, UK.*

The objective of this ongoing study is to assess the feasibility and toxicity of neoadjuvant chemotherapy before radical radiotherapy for transitional cell carcinoma of the urinary bladder. We specifically wish to assess whether by giving chemotherapy first we compromise the ability to give radical radiation; delay the palliation of distressing symptoms; or allow progression of tumour.

Patients considered suitable have been those with clinically T3 or T4 histologically proven transitional cell carcinoma of the bladder. Such patients have received three courses of chemotherapy with methotrexate (200 mg m$^{-2}$ i.v. with folinic acid rescue) plus cisplatin (100 mg m$^{-2}$), the dose of these being adjusted in the presence of poor renal function. Radical radiotherapy (52.5 Gy in 20 fractions in 26 days, three field plan) is then started after full restaging. Further full assessments of toxicity and tumour response are performed 1 and 6 months after the completion of treatments.

To date 13 patients have been treated in this way. We have found the chemotherapy to be extremely well tolerated but that reductions in the dose of the drugs have been necessary in the majority of courses. We have not found it necessary to adjust the standard radiotherapy as a result of prior chemotherapy. In all patients with symptoms there has been a symptomatic response during chemotherapy and we conclude that so far such neoadjuvant chemotherapy does not compromise later radical radiotherapy. To date we have not documented progression of local disease during the chemotherapy phase of this combined treatment.

**A comparison of remote afterloaded and manually inserted caesium in the treatment of carcinoma of the cervix**

R.D. Jones, R.P. Symonds, J. Laurie, T. Habeshaw, E.R. Watson & D.A. Lamont

*Beatson Oncology Centre and West of Scotland Cancer Surveillance Unit, Glasgow, UK.*

Between 1982 and 1985, 240 patients with carcinoma of the cervix were treated with radical radiotherapy. One hundred and forty were treated with the selection as the intracavity component of their therapy, and 100 received conventional caesium.

To allow for the increased dose rate to point A with the selectron (1.2–1.4 Gy h$^{-1}$) compared with caesium (0.55 Gy h$^{-1}$) a correction taking account of the cumulative radiation effect (CRE) formula was applied.

The overall 2-year disease free survival was comparable in the two groups (68.6% selectron *vs* 73% caesium) but in stage II patients the local control rate in the selectron group tended to be poorer (67.4% *vs* 85.7%, $P<0.10$). This difference may at least in part be due to the bulkier tumours in the selectron stage II group. There appeared to be no increase in late complications in patients treated by selectron (12.9%) compared with conventionally treated patients (15%).

The introduction of the selectron has brought about a marked reduction in staff radiation exposure. In the Royal Beatson Memorial Hospital the annual radiation exposure of nursing staff fell from 19 mSv in 1981 to 2.4 mSv in 1985.

**Young age as a prognostic factor in cervical cancer**

R. Kalra, E. Sugden & C.J. Alcock

*Department of Radiotherapy and Oncology, Churchill Hospital, Oxford, UK.*

The importance of young age as an independent prognostic factor remains uncertain. Retrospective analysis of 1,027 patients referred to the Department of Radiotherapy and Oncology in Oxford between January 1970 and December 1987 has shown an increase in the annual total of patients from 42 to 69 in this period. This is principally due to an increase in the number of patients under 40 years of age. Younger patients presented with earlier disease (FIGO stage I and II age $<40$: 93%, age $\geqslant 40$: 72%). The proportion of adenocarcinoma was 17% for age $<40$ and 13% for age $\geqslant 40$. There was no real difference in the incidence of pelvic node metastases at Wertheim's hysterectomy between the two age groups over the period studied. These results and an analysis of survival will be discussed further.

**Mortality in patients with testicular cancer: review of results of treatment**

R.T.D. Oliver, M.V. Williams, M.J. Ostrowski, W. Jackson & H. Baillie-Johnson

*London, Addenbrookes and Norfolk and Norwich Hospitals, UK.*

The previous review of results from East Anglian region treated between 1980 and 1984 reported 10.8% mortality, only 70% of which were due to progressive disease. In an attempt to improve on these results, The Anglian Germ Tumour Group agreed to standardise treatment with surveillance for stage I seminoma, and malignant teratoma and three day etoposide prolonged infusion bleomycin and cisplatin (EBCi (3)) for all patients with metastases, although one centre continued to pilot a phase 2 study of single agent cisplatin in patients with metastatic seminoma. Three hundred and twenty-eight patients have been reviewed and currently 93% are alive and disease-free, including 85% of 105 with metastatic malignant teratoma (61% of 23 patients considered very advanced by MRC criteria); 90% of 50 patients with metastatic seminoma; and 98% of 162 patients with stage I tumours (100 teratoma, 64 seminoma). Multivariate analysis demonstrated hCG level and extent of lung metastases, are the most important prognostic factors for drug resistance in patients with metastases and malignant teratoma undifferentiated had the highest frequency of relapse on surveillance (MTU 56%, MTI 68%, seminoma 76% continuously disease-free at 5 years).

**Prediction of outcome in ovarian cancer using tumour activity indices**

C.W.E. Redman, D.M. Luesley, K. Ward, E.J. Buxton & K. Kelly

*University Dept of Obstetrics and Gynæcology and CRC West Midlands Clinical Trials Unit, Birmingham, UK.*

The management of EOC is largely consequent on FIGO stage and the presence or absence of residual disease after primary surgery, which are important prognostic factors but indirect indices of tumour biology. Direct assessment of tumour cell activity may enhance prediction of outcome, but conventional parameters, such tumour differentiation are subject to considerable observer error and limited by tumour heterogenicity. Flow cytometry has enabled the objective assessment of cellular morphology and activity, which can also be biochemically evaluated by measuring products of cellular metabolism, such as cyclic 3′5′ guanosine monophosphate (cGMP). Using paraffin-embedded formalin-fixed material obtained from the primary operation, an analysis of the correlation between nuclear ploidy and the proliferative index (PI) as quantified by flow cytometry with random pre-treatment urinary cGMP was performed in 40 EOC patients. The majority of the study group had advanced disease (28 FIGO III/IV) and residual disease (31). All but three (stage I) patients received single agent high dose *cis*-platinum as first-line therapy ($100\ \mathrm{mg\ m^{-2}} \times 5$); in patients with evaluable disease there was a response rate of 64%. Thirty three patients have died; the median survival of the study population being 17 months. There was a significant correlation between cGMP and PI. Significantly more aneuploud tumours had elevated PI values ($\chi^2_1 = 0.007$). No prognostic variable independently predicted for response. An initial univariate log rank analysis analysis identified stage, the amount of residual disease, histological type, and PI as prognostic factors but because of the interrelation between these and other factors, and as a number of factors did not conform to the proportional hazards model, a multivariate stepwise discriminant analysis was performed using survival at 46 months (the minimum follow-up for surviving patients) as the end point. On the basis of this cGMP was the most prognostically important proliferative activity factor, which in conjunction with residual disease status can correctly predict outcome in 80% of cases.

**Smear cytology post-radiotherapy for squamous cell carcinoma cervix. A worthwhile test?**

S.J. Whitaker, P.R. Blake & P.A. Trott

*Royal Marsden Hospital, London, UK.*

Radical radiotherapy can induce changes in exfoliated epithelium which makes the diagnosis of recurrent disease by cytology difficult. We present a review of our routine screening policy at the Royal Marsden Hospital (RMH), to evaluate its place in the early detection of persistent and recurrent disease.

From hospital and cytology records from 1975 to 1988 we identified 22 cases of recurrent squamous carcinoma cervix post-RI. Eleven were recurrent at initial presentation. All had received radical RT. Their initial stages were: Ib, six cases; IIa, four; IIb, five and IIIb, four cases. We also reviewed the results of all follow-up smears from 1987 and 1988 and their clinicopathological correlation.

The median time to diagnosis of persistent and recurrent disease overall was 8 months (2–50). This was shorter for more advanced FIGO stages, 21 months for stage Ib, 18.5 months for IIa, 9.5 months for IIb and 2.7 months for stage IIIb. The features of their smears are presented. Of smears in 1987, 140/170 (89%) were normal, and in 1988, 160/212 (81%) were normal examinations. Two of five smears reported C4 or 5 in 1987 were false positive and 0/6 in 1988, giving a specificity of 60% and 100% respectively. Twelve patients had normal smears but abnormal clinical findings in 1987. Three had recurrence confirmed giving a false negative rate of 3/152. There were no proven recurrences with normal smears in 1988, giving a sensitivity of 98% and 100% for 1987 and 1988. In addition we identified three subclinical, operable recurrences by cytology, proven at subsequent EUA and biopsy.

Routine screening cytology is a useful and reliable adjunct to regular pelvix examinations for follow-up of irradiated squamous carcinoma cervix.

**Evaluation of a semi-automated tetrazolium assay in chemosensitivity testing of ovarian malignancy**

J.K. Wilson, J.M. Sargent, A.W. Elgie, C.G. Taylor & J.G. Hill

*Pembury Hospital, Tunbridge Wells, UK.*

We describe a feasibility study using Mosmann's 3-(4,5-dimethylthiazol-2-yl)-2,5 diphenyltetrazolium bromide (MTT) assay for assessing the chemosensitivity of primary ovarian tumour samples to cytotoxic drugs. Ascitic fluid and/or tumour biopsy samples have been collected by paracentesis or at laparotomy from patients with ovarian malignancy. Single cell suspensions were prepared and the viability and morphology assessed. The cells were continuously exposed to cytotoxic drugs for 48 h in microtitre plates. The MTT assay, which assesses mitochondrial activity, was used to determine the number of living cells remaining. To validate the assay we have demonstrated the linear relationship between cell numbers and the optical densitiy of the formazan they produce from the tetrazolium salt. Cell survival after drug exposure was expressed as a percentage of control cells. Dose–response curves for each drug were obtained. We found the drug effects varied considerably between each sample tested. Cells obtained from ascitic fluid and solid tumour in the same patient showed very similar drug effects.

In view of the disappointing results of clonogenic assays in this type of tumour, we believe this simple, rapid, semi-automated, non-clonogenic assay may offer a better opportunity for chemosensitivity testing in ovarian malignancy, with the possibility of selecting agents for each individual.

### Radiotherapy

**Dosimetry of neck irradiation**

R.A. Eeles, M. Chow, W.P.M. Mayles & J.M. Henk

*Royal Marsden Hospital, Sutton, Surrey, UK.*

When treating cancer of the head and neck, irradiation of the neck is often given using a single anterior field with a central block, 2 cm wide, to shield the spinal cord and larynx. The aim of this study was to look at the doses received by the different lymph node groups in the neck, the effect of the central block, and the dose distribution at the match line of an anterior neck field and a lateral field, such as that used in treating nasopharyngeal carcinoma.

Five patients, with different sized necks, were CT scanned in the treatment position. The position of the various neck node groups, i.e. the anterior cervical chain including the scalene nodes, the posterior chain, and the retropharyngeal nodes, were marked on each CT slice. Plans were done at 1 cm intervals using the TARGET and experimental JCPLAN systems. Doses to nodes in the posterior cervical chain were between 70 and 80% of the applied dose when the anterior field is given using a 6 MV beam. Other beam combinations give a better distribution: either a single anterior 10 MV or a mixture of 60 MV and 25 MV anterior beams, or the addition of a posterior field to the 6 MV anterior field.

Scalene nodes at the inferior end of the anterior cervical chain may be shielded by the central block if it is 2 cm wide throughout its length.

These findings may be important when treating cancers of the head and neck where there is a high incidence of metastatic disease in the posterior cervical nodes, such as nasopharyngeal carcinoma.

### The effect of radiotherapy for squamous carcinoma of head and neck mucosa on parotid salivary composition

M.N. Gaze, J.R. Barton & M. Riad

*Departments of Clinical Oncology, Medicine and Otolaryngology, University of Edinburgh, Western General Hospital and Royal Infirmary, Edinburgh, UK.*

To investigate the effects of malignancy, radiotherapy and smoking on mucosal immunity, the flow rate and immunoglobulin (Ig) profile of pure parotid saliva were measured in both healthy controls and patients with squamous carcinoma of head and neck mucosa. The controls were either smokers (47) or non-smokers (30). The patients were either untreated (30) or previously irradiated (30). In addition paired samples were obtained from 14 patients prior to and on completion of radical radiotherapy.

It was found that smoking significantly decreased salivary IgA and increased IgM ($P < 10^{-5}$). This effect was related to the number of cigarettes smoked ($P < 10^{-5}$), and reversed in former smokers. Similarly salivary IgA was significantly decreased, and IgM increased in cancer patients compared with controls, but this finding did not change with treatment, although flow rate increased with radiotherapy.

The observed changes in cancer patients may simply reflect the fact that most were smokers. The possibility that smoking-induced mucosal immunodeficiency may be an important aetiological factor in upper aerodigestive tract cancer merits further investigation.

### The recording of morbidity due to radiotherapy

E. Lartigau, S. Dische & M. Warburton

*Marie Curie Research Wing, Mount Vernon Hospital, Northwood, UK.*

In the trial of methods to improve radiotherapy, morbidity (early and late) must be monitored with the same care as tumour control. Although there are universally agreed systems for the staging of tumours, the recording of radiation dose and the expression of tumour response, there is still no generally employed scheme for the measurement and recording of radiation morbidity. Morbidity consists of symptoms, complications, investigations and treatment. It is possible to abstract these elements and to record them individually. A dictionary of recording of radiation morbidity has been developed in this centre and a fourth draft is available. It is divided into 34 sites, from adrenal gland to vagina and cervix. For each site a list of basic and optional items has been prepared. For a treated area, more than one of the site lists is, in general, required; however, it may be decided that only a few items in each list should be used. Finally, from these elements a simple computer program can grade the severity using any previously established grading system or any new one which may be devised. We have used this dictionary prospectively since 1986 for the recording of early and late reactions in patients treated by our accelerated hyperfractionated regimen (CHART). For 196 patients treated so far we have used the system at each follow-up clinic with a high level of agreement between observers. Result of the regimen can be given according to any early or late side-effect. We are now doing a retrospective clinical study comparing three groups of 20 patients previously treated by CHART, conventional or hypofractionated radiotherapy for ENT carcinoma. This study will permit us to assess the sensitivity of the system by comparing different treatment methods with different expected morbidity. After the current final revision in consultation with up to 90 centres worldwide, we expect that such a system for recording morbidity will be internationally agreed and accepted.

### BNLI results of initial treatment using radiotherapy in the treatment of non-Hodgkin's lymphoma of the palatine tonsil

C.H. Macmillan, G. Vaughan Hudson, M.H. Bennett, B. Vaughan Hudson & A.R. Makepeace

*British National Lymphoma Investigation, University College and Middlesex School of Medicine, London, UK.*

Between 1974 and 1988, 56 patients with stage IE ($n = 24$) and IIE ($n = 32$) non-Hodgkin's lymphoma (NHL) of the palatine tonsil registered with the British National Lymphoma Investigation (BNLI) were treated primarily with radiotherapy alone.

The sex ratio was equal. Mean age overall was 59 (range 25–89). Overall CR rate at 3 months post-treatment was 75% (42/56) (stage IE 83% (20/24), IIE 72% (23/32)). Thirteen patients failed to enter CR; two failed within the radiation field, one 'in-field' and generalised, two in an adjacent site, five intra-abdominally, one in the groin, one at unspecified site, and one died of CVA at 2 months. Fourteen patients relapsed after achieving CR, sites of relapse were: intra-abdominal (7), adjacent (2), 'in-field' (1), bone (2), CNS (1) and groin (1). Only four patients were either not controlled locally by RT or experienced first relapse in the irradiated area.

Twenty-three patients have died. In 16 instances (70%), death was either due to NHL or the patient had active disease when last seen.

There was a significant correlation between survival and age at presentation, with younger patients faring better. A significant difference in disease-free survival existed between stages I and II in favour of stage I, but there was no significant difference in survival overall.

### A feasibility study of twice daily continuous fractionated radiotherapy in a range of malignant tumours

M. Moriatry, M. Pomeroy, M. Maher & I. Fraser

*Saint Luke's Hospital, Dublin 6, Ireland.*

In 1987 Saunders and Dische presented preliminary results of a continuous hyperfractionated radiotherapy (CHART) regime used at two sites. The results were exciting and are being investigated further. Their schema involved a three times daily regime. A twice daily schema is more practical in our centre and the objectives of the study are to test the feasibility and toxicity of a twice daily continuous hyperfractionated schema and also to explore the possibilities of introducing chemotherapy in sequence with this radiotherapy regime.

It is aimed to treat up to 60 patients in this study by July 1989. So far 23 patients have been treated: (four) H and N, eight breast carcinoma, five lung carcinoma, four brain glioma (III or IV) and two recurrent pelvic lesions. In general volumes are kept as small as possible and doses used are TD of 5,000–5,250 rad, 30 treatments, 15 days (>6 h between fractions). Chemotherapy in conjunction has not been used yet.

Results of acute normal tissue tolerare are very acceptable for brain, breast and lung tissue. The acute reaction for two of the head and neck cases has been judged to be severe (grade III and IV) and to last for up to one month post treatment. Small bowel tolerance was a problem in one of the two cases of pelvic R/T and treatment was stopped at 4,000 rad TD. The study is ongoing with the introduction of sequenced chemotherapy into the brain, lung and breast cases now.

**Radiobiological comparisons between low dose-rate and fractionated TBI**

J.A. O'Donoghue

*Radiation Oncology Research Group, Beatson Oncology Centre, Belvidere Hospital, London Road, Glasgow, UK.*

The LQ model was used to compare fractionated TBI with single doses given at a low dose-rate (LDR). Fractionated and LDR treatments of the same total dose (i.e. same antileukaemic effect) may also produce equal lung damage. The table shows two fractionated TBI schedules and the predicted irradiation times for equivalent LDR treatments. These times depend on lung repair kinetics and results for three different half-times are shown. The irradiation times for LDR treatments appear to be impractically long.

Fractionated LDR treatments were also examined. Taking an $\alpha/\beta$ ratio of 3 Gy and a repair half-time of 1.5 h for lung the irradiation times predicted to enable a specified dose increase are shown in the second table. There appears to be little to be gained by this approach.

| *Repair half-time (h)* | *6 × 2 Gy* | *10 × 1.4 Gy* |
|---|---|---|
| 1.0 | 16 h | 27 h |
| 1.5 | 24 h | 41 h |
| 2.0 | 31 h | 55 h |

| *Fraction size* | 5% | 10% | 15% |
|---|---|---|---|
| 2.0 Gy | 1.1 h | 2.3 h | 3.5 h |
| 1.4 Gy | 1.4 h | 2.9 h | 4.7 h |

**Double half body irradiation for drug-resistant multiple myeloma: an effective and well tolerated salvage regimen**

J.S. Tobias, F.J. Giles, C.R.J. Singer, J.D.M. Richards & A.H. Goldstone

*Depts of Radiotherapy, Oncology and Haematology, University College Hospital, London WC1, UK.*

Out of a total of 89 patients treated with single or double hemibody irradiation for drug resistant myeloma, 42 have received both upper and lower halves (DHBI). All had disease progression despite standard regimens of melphalan/ prednisone (median eight courses, range 4–24); seven had primary chemoresistance. Four had also been treated with VAD, three with ABCM. Fifteen had previously undergone localised radiotherapy. Evidence for drug resistance: rising immunoglobulin (27 pts), pain (33), hypercalcaemia (7), increasing proteinuria (7). Fourteen had severe lytic bone lesions. Using standard MRC criteria, six pts were stage 1, 22 stage 2 and 14 stage 3. Treatment consisted of single-fraction upper HBI to 7.5 Gy using anteroposterior fields on $^{60}$Co at SSD 200 ± 12 cm, dose rate 0.2–2.25 Gy min$^{-1}$, treatment time 40–60 min. After min 5 weeks we treated the lower half to total 10 Gy (single fraction with dose rate and position as before). Treatment was well tolerated with no treatment deaths though three pts died within 3 months from disease. Platelet transfusion (Tx) was needed in seven pts (mean period 3 weeks) and red cell Tx in 40, after the second HBI (usually a single Tx). Response included symptomatic benefit (30/33 pts with pain relief, many discontinuing opiates); 22/ 27 >25% fall in M-band; four reversal of Bence Jones proteinuria, six hypercalcaemia. Mean survival is 12 mth; 19 are alive, six at > 2 years without further treatment. Currently, patients are randomised after DHBI to receive subcutaneous $\alpha$-interferon as maintenance (Intron, Kirkby-Warrick, 3 MU 3 times a week) or no further treatment.

**Radiotherapy for cholangiocarcinoma: the Hammersmith Hospital experience**

K.A. Vallis, H.R. Gompertz, I.S. Benjamin & A.J. Munro

*Department of Clinical Oncology, Hammersmith Hospital, UK.*

Cholangiocarcinomas are rare tumours which, because of their anatomical location, are only occasionally curable by surgery alone. They tend to be slowly growing tumours and some encouraging results have been reported with radiotherapy. Between August 1986 and January 1989, 29 patients with cholangiocarcinoma were referred from the Hepatobiliary Unit at the Hammersmith Hospital for a radiotherapy opinion. The mean age of these patients was 55.4 years with a range of 22–79 years. The male to female ratio was 2.6:1.

Of these 29 patients, eight had undergone either radical excision of tumour or palliative bypass procedure. In the remainder biliary stents were inserted to relieve obstructive jaundice. Nine patients were considered to be in such poor condition that radiotherapy was not indicated. The radiotherapy regimes used were as follows: external beam therapy alone (six patients); external beam therapy combined with interstitial therapy (eight patients); and interstitial therapy alone (six patients). Brachytherapy was given in the form of iridium-192 wire inserted via a percutaneous trans-hepatic catheter.

Of the 20 patients treated by radiotherapy, follow-up data are available for 19. Ten patients have died, with a mean survival of 9 months after radiotherapy. Nine patients are alive with a mean follow up of 10 months.

The technique of intracavitary treatment for cholangiocarcinoma will be described and the data on this series of 29 patients presented.

**The relation of tumour curability to tumour size for biologically targeted radiotherapy**

T.E. Wheldon, J.A. O'Donoghue & A. Barrett

*Beatson Oncology Centre, Belvidere Hospital, Glasgow and Department of Clinical Physics, University of Glasgow, UK.*

In conventional radiotherapy, smaller tumours are invariably more radiocurable than larger ones. In targeted radiotherapy, the lesser cell content of smaller tumours may be offset by reduced efficiency of absorption of radioactive disintegration energy from radionuclides which are long range $\beta$-emitters. We have used a mathematical model based on Poisson statistics to evaluate relative curability of tumours with cell content from $10^1$ to $10^{10}$ cells using targeted $^{131}$I or $^{90}$Y. The analysis made use of available microdosimetric data on energy absorption by very small spheres. Computer simulations were carried out for a wide range of therapeutic radionuclide activities (e.g. 0–1000 mCi $^{131}$I) for varying specific uptake of activity by tumour cells (e.g. 0.00001–0.001 g$^{-1}$) and differing clonogenic fraction (e.g. 0.05–0.5). The results show that, for each radionuclide, there is a tumour size region within which tumour radiocurability is maximum. For $^{131}$I, the optimal cure region is close to approximately $10^6$ cell content, while for $^{90}$Y it is close to approximately $10^8$ cell content. Above this cell content, curability decreases due to increasing cell number. Below this cell content, curability decreases due to reduced efficiency of energy absorption. Targeted radiotherapy with $^{131}I$ or $^{90}Y$ differs from conventional radiotherapy in that microscopic tumours may be less easily cured than small macroscopic ones. Computer simulation studies suggest that a combination regime with both targeted and conventional radiotherapy may be the optimal strategy for treatment of patients with disseminated disease consisting of tumours and micrometastases of differing size.

### Central nervous system, lung, and head and neck cancer

### Long-term survival in gliomas: prognostic factors and quality of outcome

S. Awwad, A. Cull & A. Gregor

*Department of Clinical Oncology, Western General Hospital, Edinburgh, UK.*

One hundred and eleven patients with previously untreated gliomas were referred to the Department of Clinical Oncology in Edinburgh between 1983 and 1985. There were 79 males, 32 females and 11 children (<15 years); 22 were 15–45 years; 28 were 46–55 years and 49 patients were older than 55 years at presentation. One hundred and one patients had hemispheric tumours. Histological confirmation was obtained in 87 (78.4%). Sixty-seven were classified as astrocytomas. Surgical removal was attempted in 61 patients (complete in 22 and partial 39). Twenty-nine patients were biopsied and in 21 diagnosis was not histologically confirmed. One hundred and three patients underwent radical radiotherapy. Fifty-two mixed beam of photons (2,040cGy in 12 fractions over 4 weeks) and neutrons (600cGy in 12 fractions over 4 weeks). Fifty-one patients received pure photon beam 4,736 cGy average central dose in 20 fractions over 4 weeks. Whole brain irradiation was used in all but 12 patients (11 children). Minimal follow-up of 3 years is available on all patients. Five year actuarial survival is 6.2% for the whole population and 4.7% if children are excluded. Prognostic factors significantly influencing survival are age at diagnosis ($P = 0.007$), histological grade of astrocytoma, grade I + II = 15%, III = 4%, IV = 0% ($P = 0.005$). Duration of symptoms, extent of surgical resection and photon radiotherapy show a trend in improved survival. Detailed assessment of survivors including neuro functional, psychometric, hormonal and radiological assessment is in progress and will be presented. Significant proportion of long term survivors show late morbidity seriously effecting their quality of life.

### The influence of MRI on the treatment of spinal cord compression

J.D. Graham, P.J. Hoskin & M. Williams

*Royal Marsden Hospital, Sutton, UK.*

In the treatment of spinal cord compression by radiotherapy, conventionally a 5 cm margin (or two vertebral bodies) is given above and below the site of compression. In the diagnosis of spinal cord compression the introduction of magnetic resonance imaging (MRI) has significantly improved the quality of diagnostic images compared to myelography. No study has investigated the influence of MRI on clinical decision making in spinal cord compression.

Eleven cases have been reviewed in which both myelography and MRI were performed prior to treatment. Taking account of clinical findings, the results of each diagnostic technique were independently reviewed and an appropriate radiotherapy volume proposed. The field lengths based on MRI were significantly less than those based on myelography ($P < 0.02$). However, the actual field sizes used did not reflect the additional information from MRI. In only one case did MRI add information which significantly altered the radiation field. Myelography remains an accurate method of diagnosis for the majority of patients and this study suggests that smaller margins above and below the radiographic site of compression may be used. It is recommended that a 2 cm or one vertebral body margin should be adequate.

### The influence of site and local management on the outcome of radiotherapy for cerebral metastases

P.J. Hoskin, J. Crow & H.T. Ford

*Royal Marsden Hospital, Sutton, Surrey, UK.*

A retrospective analysis of 164 patients treated with radiotherapy for cerebral metastases between January 1978 and January 1988 will be presented. These patients reflect a policy of whole brain irradiation, delivering a mid-plane dose of 35 Gy in 15 daily fractions. In selected patients having a solitary localised site of metastasis and stable or absent disease elsewhere a boost of 15 Gy in eight daily fractions was given. These patients had predominantly primary tumours of lung (52%) or breast (29%); other primary sites included melanoma (8%), kidney (3%), bladder (2%) and colon (2%). The diagnosis of cerebral metastases was confirmed by CT scan in 144 (88%) patients, and by isotope scan in the remainder.

Oral dexamethasone was given to all but 11 patients. One hundred and fourteen (69.5%) received whole brain irradiation alone and 50 (30%) received the additional boost. Median survival (range) in patients receiving no boost was 116 days (13–1,436) compared to 140 days (32–974) in those receiving a boost. Progressive cerebral metastases were a cause of death in 43 (26%) of all patients, 13 of whom received a boost. Despite the selection criteria for receiving a boost, 30 (60%) of those who did so died with progressive distant metastases outside the brain, 10 (20%) died with progressive primary tumour.

### Combination chemotherapy (C/T) with mitomycin, ifosfamide and cisplatin in advanced non-small cell lung cancer

D.W. Miles, H.M. Earl, J. Drake, D.C. Currie, S.G. Spiro, R.L. Souhami, P.G. Harper & J.S. Tobias

*Guy's Hospital, University College Hospital, and Brompton Hospital, London, UK.*

Mitomycin C (M), ifosfamide (I) and cisplatin (C) are among the most active single agents in non-small cell lung cancer (NSCLC). A recent report (Cullen *et al.* (1988) *Br. J. Cancer*, **58**, 359) showed an overall response rate of 56% in 74 patients (67% LD, 35% ED). We report a further study to confirm these findings. Forty-five patients (six previously treated with surgery ± R/T or C/T ± R/T) were treated with M 6 mg $m^{-2}$, I 3 g $m^{-2}$ (with MESNA protection) and C 50 mg $m^{-2}$, every 21 days. Patients were reassessed after two courses of C/T and, if responsive, treatment was continued to four course in 18 cases and up to six courses in four cases. There were 45 pts: ECOG 0–2, 34 M, 11 F, 18 limited disease (LD), 27 extensive disease (ED), median age 57, range 29–67.

| | All pts n (%) | Squamous | Adeno | Large cell | LD | ED |
|---|---|---|---|---|---|---|
| No pts | 45 | 23 | 10 | 12 | 18 | 27 |
| OR | 18 (40%) | 11 (48%) | 2 (20%) | 5 (42%) | 8 (44%) | 10 (37%) |
| CR | 4 (9%) | 1 (10%) | 1 (10%) | 2 (17%) | 2 (11%) | 2 (7%) |
| PR | 14 (31%) | 10 (44%) | 1 (10%) | 3 (25%) | 6 (33%) | 8 (30%) |

Median survival = 36 weeks (LD 41 weeks, ED 25 weeks).

The major toxicity was gastrointestinal (GI) of 42 pts assessible for GI toxicity, 13 experienced WHO grade III nausea and vomiting, one patient had intractable (grade IV) vomiting. All pts developed alopecia. There was no significant nephrotoxicity. On five occasions pretreatment WBC was less than $3.0 \times 10^9 l^{-1}$ and treatment was delayed.

These results confirm that MIC is an active regimen in NSCLC, but the results in LD are not equal to those of Cullen *et al.*

### Chemotherapy or total body irradiation for limited or extensive small cell carcinoma of the bronchus

G.A. Newaishy

*Clinical Oncology Department, Edinburgh, UK.*

A controlled trial of localised radiotherapy in combination with either chemotherapy or total body irradiation (TBI) was planned for patients with small cell carcinoma of the bronchus. The trial continued non-controlled due to the low intake. Systemic treatment was to be given as adjuvant or at recurrence. The TBI using small fractionated doses (150 cGy in 15 fractions) was ineffective and was discontinued, and was not given at recurrence. Thirty-one patients received adjuvant chemotherapy and 13 had adjuvant TBI. Their 1 year survival rates (SR) were 29% and 15.4% respectively. All patients who received TBI were dead within 20 months, while 9.7% of those who received chemotherapy were alive at 5 years. The median survival (MS) of the 31 patients was 32 weeks and that of the 13 patients was 33.8 weeks. Seventeen other patients received chemotherapy at the time of recurrence. Their 1 and 5 year SR were 70.6% and 0% respectively (MS 77 weeks). Analysis was made of patients who received chemotherapy according to the stage of disease. Seventeen of 29 patients with limited disease received adjuvant chemotherapy and 12 patients received delayed chemotherapy. Five (29.4%) and three (17.6) of the 17 patients lived for 1 and 5 years respectively. Ten (83.3%) and two (16.6%) of the 12 patients lived for 1 and 4 years but were dead by 5 years. The MS of these two groups of patients were 29 weeks and 81 weeks respectively. Fourteen of the 19 patients who had extensive disease had adjuvant chemotherapy and five patients received delayed chemotherapy. Four of the 14 patients (28.6%) and two (40%) of the five patients lived for 1 year. All the 14 patients died by 16 months and the five patients died by 25 months. The MS of these two groups of patients was 37 and 42.2 weeks respectively.

### Non-small cell lung cancer: how the experts want to be treated

M.J. Palmer & W.J. Mackillop

*Kingston Regional Cancer Centre, Kingston, Canada.*

Four hundred and sixty one doctors who treat lung cancer in the USA and Canada responded to a mail questionnaire that asked how they would wish to be managed if they developed non-small cell lung cancer (NSCLC). There was no consensus as to preferred treatment in either of two clinical situations described. Most doctors (74%) wanted radiotherapy as a part of their management for NSCLC with extensive node involvement, but 28.5% wanted chemotherapy, 21.5% wanted surgery and 17% wanted no active treatment. American and Canadian doctors had different treatment preferences ($P < 0.005$). Radiation therapy, chemotherapy and surgery were most frequently favoured by radiation therapists, medical oncologists and thoracic surgeons, respectively ($P < 0.025$). Most doctors (81%) wanted radiotherapy to the painful area if they had NSCLC with bone metastases, while 28% wanted chemotherapy and 21% wanted radiotherapy to the primary lesion as part of their management. Forty-five per cent of American doctors would want treatment involving chemotherapy compared to 6% of Canadian doctors ($P < 0.001$). Chemotherapy was favoured by 49% of medical oncologists compared to 6% of radiation therapists ($P < 0.001$), whereas 12% of medical oncologists wanted radiotherapy to their chest compared to 40% of radiation therapists ($P < 0.001$). Almost all of the doctors would recommend for their patients the treatment that they chose for themselves. It was concluded that physician bias is an important factor in determining how patients with NSCLC are treated. Doctors were also asked if they would participate in three clinical trials if they developed NSCLC. Only 22–46% of the doctors were willing to consent to each of the trials, but almost twice as many were willing to ask their patients to participate.

### The management of tumours arising in the maxillary antrum

G.S. Rao, A.G. Robertson, D.S. Soutar & A. Al-Sammarie

*Canniesburn Hospital, Bearsden, The Institute of Radiotherapy, Kuwait, and Beatson Oncology Centre, Glasgow.*

Eighty-eight cases of tumours arising in the maxillary antrum underwent treatment between 1967 and 1989. The series comprised 34 females and 54 males, with ages ranging from 9 to 93 years and an average of 63 years.

Follow-up varied from 6 months to 14 years with a minimum follow-up of 6 months.

Sixty-two patients had squamous cell carcinomas, the remaining various histologies including adenoid cystic carcinoma, sarcoma and lymphoma. All patients were staged retrospectively (UICC, 1984). Sixty-two cases of squamous cell carcinoma were treated either by XRT (41) or surgery and post-operative XRT as a combined modality treatment (2). Nodal involvement at the time of presentation occurred in 6 cases and was associated with a poor prognosis, all patients dying of tumour within 3–66 months following treatment.

Early tumours (T2 NO) were adequately controlled by radical radiotherapy alone with a 5-year survival of 69%. In more advanced tumours (T3 NO and T4 NO) radical radiotherapy alone was less successful with the 5-year survival falling to 19%. Combined modality treatment comprising radical surgery followed by radical post-operative radiotherapy improved 5-year survival in advanced tumours to 61%.

### Parotidectomy for malignant salivary tumours and for metastatic melanoma in pre-auricular lymph nodes

A.B.S. Ball & J. Meirion Thomas

*Westminster and Royal Marsden Hospital, London, UK.*

Formal parotidectomy was performed in 120 patients by one surgeon over 7 years. Eighteen patients had malignant salivary tumours, of which eight were recurrent. The policy was to treat all untreated high-grade tumours (HGT) by preoperative radiotherapy. Low-grade tumours (LGT) (some muco-epidermoid, all adenoid cystic and acinic cell carcinomas) were treated by surgery alone. In both groups the purpose of surgery was to achieve tumour clearance while preserving the stem and any branches of the facial nerve not already destroyed. Of 10 patients with HGT, six had complete facial palsy at presentation due to tumour. Of eight patients with LGT, two had partial facial palsy due to previous surgery. Following surgery only one patient suffered further permanent deterioration of facial nerve function. The decision to perform radical neck dissection (RND) was taken at the time of surgery by biopsy and frozen section of the jugulo-digastric node which was positive in five patients, only one of whom had palpable matastatic cervical lymphadenopathy. Five patients have died from metastases 8–47 months from initial presentation, and one has local recurrence after surgery: all had HGT.

Eight patients with malignant melanoma presented with pre-auricular node metastases. Diagnosis was confirmed by cytology and each patient was treated by superficial parotidectomy and RND. Cervical nodes were positive in six. Four patients have died of metastatic disease and one has local recurrence. Effective regional node dissection for palliation of melanoma is recommended.

### Spinal cord compression due to metastatic malignancy: comparison of clinical features and magnetic resonance (MRI) images

D.J. Grant, M.P. Williams, D.W.O. Tilsley & M. Brada

*Academic Radiotherapy Unit and Department of Diagnostic Radiology, Royal Marsden Hospital, Sutton, UK.*

MRI enables the spinal cord and surrounding structures to be visualised in a non-invasive manner and has become the investigation of choice in patients with suspected spinal cord compression (SCC). Between September 1987 and July 1988 MRI demonstrated distortion of the spinal cord due to metastatic malignancy in 29 patients presenting with clinical features of SCC. The cord was compressed by bone in four and by soft tissue in 25 patients. Nineteen patients had an anterior, 10 a posterior, seven a lateral and five more than one component to the direction of compression. The length of lesion was 1–12 cm and was 3 cm or larger in seven patients.

We were not able to demonstrate a significant correlation between the anatomical details obtained from MRI images (direction, length and the anatomical level of compression) and the presenting clinical features (the type of neurological deficit and the degree of motor impairment).

Planned course of radiotherapy was completed as the sole mode of treatment in 23 patients. Nine improved neurologically. The pre-treatment MRI images did not predict the results of therapy.

The additional information obtained from MRI allows for more accurate delivery of radiotherapy, but the anatomical detail does not correlate with functional deficit and the outcome of treatment.